# Community-based group physical activity and/or nutrition interventions to promote mobility in older adults: an umbrella review

**DOI:** 10.1186/s12877-022-03170-9

**Published:** 2022-06-29

**Authors:** Sarah E. Neil-Sztramko, Kylie Teggart, Caroline Moore, Diana Sherifali, Donna Fitzpatrick-Lewis, Giulia Coletta, Stuart M. Phillips, K. Bruce Newbold, Elizabeth Alvarez, Ayse Kuspinar, Courtney C. Kennedy, Pasqualina L. Santaguida, Rebecca Ganann

**Affiliations:** 1 Department of Health Research Methods, Evidence and Impact, Faculty of Health Sciences, McMaster University, 175 Longwood Rd S, Suite 210a, Hamilton, ON L8S 4K1 Canada; 2 School of Nursing, Faculty of Health Sciences, McMaster University, Hamilton, ON L8S 4K1 Canada; 3 Department of Kinesiology, Faculty of Science, McMaster University, Hamilton, ON L8S 4K1 Canada; 4 School of Earth, Environment & Society, Faculty of Science, McMaster University, Hamilton, ON L8S 4K1 Canada; 5 School of Rehabilitation Science, Faculty of Health Sciences, McMaster University, Hamilton, ON L8S 4K1 Canada; 6 Department of Medicine, Division of Geriatrics, McMaster University, Hamilton, ON L8S 4K1 Canada

**Keywords:** Mobility, Nutrition, Older adults, Physical activity, Umbrella review

## Abstract

**Background:**

Physical activity and a healthy diet are important in helping to maintain mobility with aging. This umbrella review aims to identify group-based physical activity and/or nutrition interventions for community-dwelling older adults that improve mobility-related outcomes.

**Methods:**

Five electronic databases (MEDLINE, Embase, CINAHL, Cochrane CENTRAL, Sociological Abstracts) were searched from inception to December 2021. Eligibility criteria included systematic reviews exploring the effectiveness of physical activity or structured exercise, alone or combined with nutrition interventions on mobility-related outcomes (aerobic capacity, physical function, balance, falls/safety, muscle strength, health-related quality of life/wellbeing). Interventions must have been delivered in a group setting to community-dwelling older adults aged 55+. Two reviewers independently performed eligibility screening, critical appraisal (using AMSTAR 2) and data extraction. The GRADE approach was used to reflect the certainty of evidence based on the size of the effect within each mobility-related outcome category. Older adult/provider research partners informed data synthesis and results presentation.

**Results:**

In total, 62 systematic reviews (1 high, 21 moderate, 40 low/critically low quality) were identified; 53 included physical activity only, and nine included both physical activity and nutritional supplements. No reviews included nutrition interventions alone. Combined aerobic/resistance, general physical activity, and mind-body exercise all improved physical function and balance (moderate-high certainty). Aerobic/resistance training improved aerobic capacity (high certainty). Resistance training and general physical activity improved muscle strength (moderate certainty). Aerobic/resistance training and general physical activity are likely to reduce falls among older adults (moderate certainty). There was no evidence of benefit for nutritional supplementation with physical activity.

**Conclusions:**

Group-based physical activity interventions that combine aerobic and resistance, general PA and mind-body exercise can improve measures of mobility in community-dwelling older adults. We found no reviews focused on nutrition only, highlighting a gap in the literature.

**Supplementary Information:**

The online version contains supplementary material available at 10.1186/s12877-022-03170-9.

## Background

Mobility is a multifaceted construct, influenced by a range of modifiable (e.g., physical, cognitive, psychosocial, financial) and non-modifiable (e.g., environmental, gender, cultural, and biographical) factors [1]. A comprehensive view of mobility reflects one’s ability to move within their immediate home environment and the broader community [1]. Reductions in mobility and the ability to carry out activities of daily living are common with aging and are recognized precursors to frailty, falls, hospitalization, and death [2–4]. Although some factors influencing mobility among older adults are non-modifiable, several modifiable risk factors have been identified, including physical function, balance, muscular strength, aerobic endurance, and psychosocial wellbeing [5, 6]. Nutritional risk factors (e.g., inadequate food/fluid intake to support optimal physical functioning) are also predictive of reduced mobility in older adults [5, 7].

The beneficial effect of physical activity (PA) and improved diet quality on modifiable mobility-related outcomes has been widely demonstrated; however, inactivity and malnutrition continue to affect the well-being and mobility of older adults [8–12]. Community-based physical activity and nutrition programs delivered in group settings can address both the physical and psychosocial aspects of mobility, promoting a sense of belonging which aids in long-term adherence [13, 14]. The Enhancing physical and community MoBility in OLDEr adults with health inequities using commuNity co-design (EMBOLDEN) trial is a multi-year program of research from XX University in [City, Country] (Trial ID: NCT05008159) [15]. The transdisciplinary team of EMBOLDEN researchers, older adults and community partners have used community-based co-design to integrate local community needs, preferences, and resources with high-quality scientific evidence to develop a mobility-enhancing program that supports physical activity, healthy eating, and social participation among older adults.

Several systematic reviews have been published exploring a broad range of PA and/or nutrition interventions for older adults, making it challenging to bring together the best scientific evidence to inform program design. Umbrella reviews provide a rigorous methodology for synthesizing evidence from multiple existing systematic reviews [16], and may be particularly useful for a phenomenon such as mobility given the wide variety of interventions and uncertainty as to which interventions are more effective when delivered individually or in combination and within different populations and/or settings. To date, two umbrella reviews have reported the effectiveness of exercise interventions in pre-frail, frail, or sarcopenic older adults [17, 18], and one umbrella review has described the impact of nutritional interventions for community-dwelling older adults on body composition [19]. Given the lack of recent, relevant synthesized evidence to meet our needs, our team undertook this umbrella review to help inform intervention design and provide a foundation for the EMBOLDEN research program. This umbrella review aims to synthesize evidence from existing systematic reviews regarding the effectiveness of group-based PA and/or nutrition interventions to improve measures of mobility in community-dwelling older adults.

## Methods

This review was conducted following the *Joanna Briggs Institute* (JBI) guidance for umbrella reviews [16], and was registered with PROSPERO (CRD42020141352). Although originally conceptualized as a systematic review, upon initiation of screening it was determined that many systematic reviews existed, and an umbrella review was most appropriate.

### Search strategy

A trained librarian conducted a search of MEDLINE, Embase, CINAHL, Cochrane CENTRAL, and Sociological Abstracts from inception to December 2021 (Additional file 1). Searches were limited to systematic reviews/meta-analyses and randomized controlled trials (RCTs) published in English.

### Study selection

Citations were imported into DistillerSR (Evidence Partners, Ottawa, Canada) and duplicates were removed. Citations were reviewed by two independent reviewers using pre-determined criteria. At the title/abstract level, a study must have been selected by one reviewer for inclusion, while exclusion required two reviewers to agree. At the full-text level, disagreements were resolved through discussion by two reviewers, with input from a third team member as needed.

### Eligibility criteria

#### Types of studies

Systematic reviews (narrative summary, meta-analysis, or network meta-analysis) of interventions were eligible. Scoping or narrative reviews that did not include critical appraisal of primary studies were excluded. Eligible reviews could include RCTs and non-randomized intervention studies, however, at least 80% of single studies included must have been interventions (i.e., not descriptive, qualitative, or observational). To balance feasibility, while ensuring we captured the most recent and relevant intervention data, we chose to include only reviews that were published in 2010 or later; although eligible reviews did include single studies that were conducted prior to 2010.

#### Types of participants

Eligible systematic reviews included studies involving community-dwelling older adults. Reviews were included if the pooled mean age or inclusion criteria identified an age of ≥ 55 years. If this information was not available, at least 70% of included studies must have reported a mean sample age of ≥ 55 years. Reviews in which studies were selected based on a specific health or disease status (e.g., cancer, sarcopenia) were excluded. In reviews that did not restrict by disease status, 70% of included studies must have been conducted in a general sample of older adults. The choice of 70% was intended to include reviews in which the majority of included studies were relevant to the general population; most studies were either well above or well below this threshold.

#### Interventions

Eligible reviews must have included single studies of any PA (any movement resulting in energy expenditure), structured exercise (planned and repetitive movements), and/or nutrition intervention (e.g., education, counselling, dietary changes and/or supplementation) that could reasonably be delivered in a group-based setting [20]. Exercise or PA interventions were categorized as: aerobic exercise, resistance exercise, combined aerobic and resistance exercise, general physical activity (reviews in which a variety of types of physical activity and/or exercise were synthesized together), mind-body exercise (e.g., Tai Chi, yoga, Pilates), and dance.

#### Context

Single studies within eligible reviews must have been delivered in a community setting. Reviews that focused exclusively on interventions delivered in hospitals, rehabilitation centers, long-term care homes, or clinics were excluded. When the reviews did not set inclusion criteria by setting, at least 70% of included single studies were required to be community-based.

#### Outcomes

Reviews must have synthesized (narratively or via meta-analysis) outcomes related to physical or community mobility. These outcomes were broadly classified into six domains based on modifiable risk factors related to mobility that could be reasonably addressed through PA and/or nutrition interventions, as described above. The mobility-related outcomes explored include aerobic capacity, physical function, balance, falls/safety, muscular strength, and self-reported mental wellbeing/quality of life. Reviews that focused exclusively on cognitive function or body composition were excluded. These criteria were not part of our original protocol as registered in PROSPERO but added at the full text screening level as the goals of these interventions and associated outcomes were quite distinct.

### Assessment of methodological quality

Eligible reviews were critically appraised using A MeaSurement Tool to Assess systematic Reviews (AMSTAR 2) [21]. AMSTAR 2 was completed independently by two reviewers, with conflicts resolved through discussion or the input of a third reviewer, as needed. Following consensus, results were entered into the online AMSTAR checklist, which provides an assessment of overall quality as critically low, low, moderate, or high based on seven critical domains [22].

### Data extraction

Data were extracted by two independent reviewers using a standardized form. Disagreements were resolved through discussion or by a third reviewer. Data were extracted related to review methodology (e.g., sources searched, publication date range, methodological quality of included studies, noted limitations) and details of included studies (e.g., study designs, participant characteristics, intervention descriptions, setting). To explore issues of equity, diversity and inclusion, any data regarding material deprivation, and the percentage of low-income and/or immigrant populations were also extracted. Results from both narrative syntheses and meta-analyses were extracted within the six outcome categories described above. Any outcomes within these categories or composite outcomes in these areas (e.g., when multiple outcome measures were grouped and reported as standardized mean difference in a meta-analysis) were extracted, as reported. Data collection forms and full extracted data are available upon request.

### Data synthesis and certainty of evidence

The Grading of Recommendations Assessment, Development and Evaluation (GRADE) approach was used to assess the overall certainty of the evidence [23]. The GRADE process was adapted to accommodate the umbrella review by considering both the findings across included reviews and across single studies within reviews by intervention type and outcome. Following the GRADE approach, reviews including primarily RCTs start at ‘high’ certainty, while reviews primarily including non-randomized studies start at ‘low’ certainty. The level of certainty was further downgraded based on the risk of bias, inconsistency of findings, indirectness of interventions/outcomes, imprecision of effect measures, and/or publication bias, and were upgraded based on the magnitude of effect size, dose-response relationship, and accounting for confounding. A narrative approach to data synthesis was used, with results presented in supporting tables and figures. Informative statements reflecting both the certainty of evidence and importance of the size of the effect are presented to communicate overall findings within each intervention type and outcome category, in line with published recommendations [24]. Only results that compared an intervention group to a control group were included in GRADE, although subgroup analyses are presented in accompanying tables.

The review team synthesized data with feedback and input from the larger research team and key stakeholders. After an initial draft, preliminary results and categories were presented to four older adult citizen and service provider partners from an established stakeholder group within the EMBOLDEN research program who were consulted via a one-time, virtual meeting. The aim of this engagement was to allow for feedback and discussion about the appropriateness of intervention/outcome groupings and to identify priority outcome measures (e.g., prioritize general physical function outcomes over measures such as body composition). Our older adult and service provider partners also contributed to developing public-facing documents, including a plain-language summary and infographic.

## Results

**Fig. 1:**
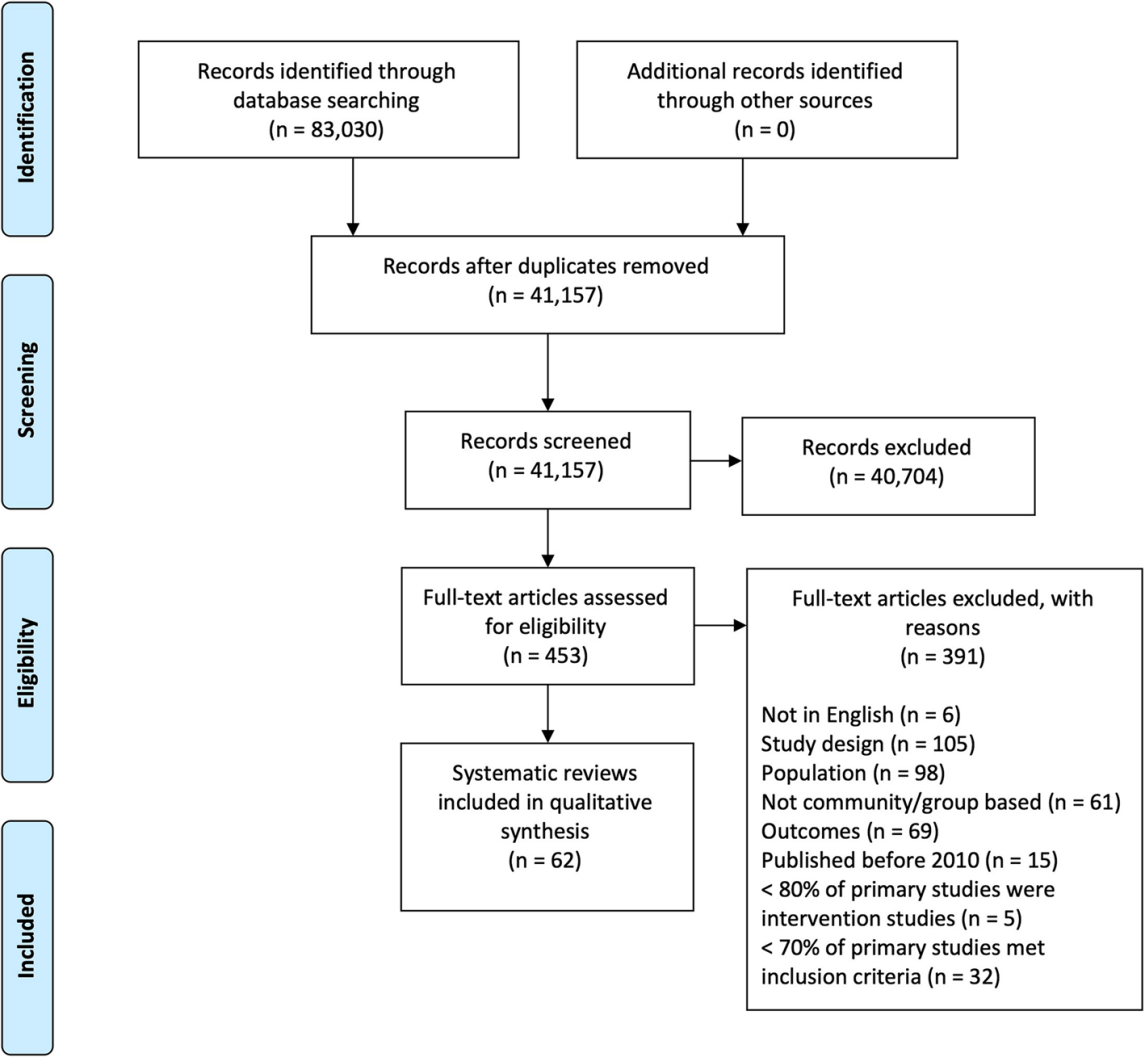
PRISMA Flow Diagram

The search identified 41,157 unique citations, of which 1547 were potentially relevant; at this point, the team elected to limit to systematic reviews (Fig. 1). A second screen identified 453 citations for full-text review, of which 62 were included (Table 1). A list of excluded studies with reasons is provided in Additional file 2.

**Table 1:** Characteristics of Included Reviews

Study	Objectives	Search	Years included	Description of intervention and comparator	Number and type of studies	Age (years)	N	% F	Study Quality (Tool)
Antoniak 2017 [25]	To assess effectiveness of RT + vitamin D3 on musculoskeletal health in older adults.	ScienceDirect, MEDLINE, PubMed, Google Scholar, Cochrane CENTRAL to Mar 2016	2003–2015	I: RT and vitamin D3 supplementation with or without calcium C: inactive, usual care without vitamin D3 supplementation	7 RCTs	Inclusion: ≥65 Mean: 72.8	792	82	RT + vitamin D: moderate RT + vitamin D + calcium: moderate-high (GRADE)
Borde 2015 [26]	To determine effects of RT on muscle strength and morphology and explore any dose–response relationships.	PubMed, Web of Science, Cochrane Library to May 2015	1991–2014	I: Machine-based RT of low, moderate, or high intensity C: inactive	25 RCTs	Inclusion: ≥60 Mean: 70.4 Range: 60–90	819	NR; 16% F only, 16% M only	Low (PEDro)
Bouaziz 2016 [27]	To evaluate the evidence of the health benefits of MCT training in adults > 65.	CINAHL, Embase, SPORTDiscus, Web of Science, Scopus, PubMed, MEDLINE, ScienceDirect, Jan 2000-Apr 2015	2000–2015	I: MCT including AT, RT, balance, stability, flexibility, and/or coordination training C: NR	27 total; 19 RCTs	Inclusion: ≥65 Range: 65–83	NR	NR	Low quality studies excluded (Tool NR)
Bouaziz 2017 [28]	To review effect of AT in adults > 70 on cardiovascular, metabolic, functional, cognitive, and QoL outcomes.	CINAHL Plus, Embase, MEDLINE, PubMed, Scopus, Web of Science, SPORTDiscus, ScienceDirect, to Jan 31, 2016	1984–2013	I: Supervised (class or small group) AT C: NR	53 total; 36 RCTs	Inclusion: ≥70 Range: 70.0–87	2051	NR	Low-moderate (Cochrane)
Bouaziz 2018 [29]	To determine the benefits of AT on VO_2_peak among those ≥70.	MEDLINE, PubMed Central, Scopus ScienceDirect, Embase to Mar 31, 2017	1989–2013	I: AT, any activity that uses large muscle groups, can be maintained continuously, and is rhythmic C: usual care or other exercise	10 RCTs	Inclusion: ≥70 Range: 70–79	348	NR; 20% F only, 10% M only	Moderate (Cochrane)
Bruderer-Hofstetter 2018 [30]	To identify effective MCT interventions for physical capacity and/or cognitive function.	MEDLINE, CENTRAL, CINAHL, PsycINFO, Scopus, Date NR	2002–2017	I: Combined cognitive training (exergames, dancing or Tai Chi) and physical exercise (planned, structured) C: attention controls or no intervention	17 RCTs	Inclusion ≥55 Mean: 71.37 ± 4.89	1758 Range: 32–555	67	Very low certainty (GRADE)
Bueno de Souza 2018 [31]	To determine the effects of mat Pilates on physical function in older persons.	MEDLINE, Scopus, Scielo, PEDro, Jan 2011-Mar 2017	2011–2017	I: Mat Pilates with or without accessories C: inactive control	9 RCTs	Inclusion ≥60 Mean: 68.5 ± 5.1	Range: 45–88	NR; 56% F only	56% high 44% low (PEDro)
Bullo 2015 [32]	To summarize the benefits of Pilates on physical fitness and QoL in the elderly.	MEDLINE, Embase, SPORTDiscus, PubMed, Scopus, PsycINFO, Date NR	2009–2014	I: Pilates-identified exercise training intervention C: inactive control	10; 6 RCTs	Inclusion: ≥60 Range: 60–80	349 Range: 9–60	NR; 50% F only	40% high 60% low (Cochrane)
Bullo 2018 [33]	To determine the effects of Nordic walking on physical fitness, QoL and body composition in the elderly.	MEDLINE, Embase, PubMed, Scopus, SPORTDiscus, PsycINFO, Date NR	2012–2017	I: Supervised or unsupervised Nordic walking C: sedentary, walking, or resistance exercise	15; 8 RCTs	Inclusion ≥60 Range: 60–92	Range: 18–95	“Majority female”	27% high 73% low (Cochrane)
Chase 2017 [34]	To determine the effects of supervised RT and/or AT on physical function among community-dwelling older adults	MEDLINE, PubMed, CINAHL, Cochrane Library, Proquest, SPORTDiscus, PEDro, Ageline, Dissertation Abstracts International, 1960–2015	1999–2015	I: Supervised intervention involving RT and/or AT. 18 studies used RT only, the remainder employed combination RT and AT C: NR	28 (designs NR)	Inclusion: > 65 Mean: 70 Range: 65–85	2608	71	NR; lower quality studies had stronger findings (PEDro)
da Rosa Orssatto 2019 [35]	To compare change in functional capacity following fast- vs. moderate-velocity lower limb RT in older adults	PubMed, Scopus, Web of Science to Jan 2019	2003–2019	I: Fast-velocity lower limb RT C: moderate-velocity lower limb RT	15 RCTs	Inclusion: ≥60 Range: 64.4–81.6	593	NR	Fair (PEDro)
Devries 2014 [36]	To determine whether the addition of creatine to RT increased gains in muscle mass, strength, and function in older adults over RT alone.	MEDLINE, HealthStar to June 2013	1998–2013	I: RT and Creatine supplementation C: RT and placebo	10 RCTs	Inclusion: > 45 Range: 55–71	357	NR; 20% F only, 40% M only	Low-moderate (Jadad)
Ebner 2021 [37]	To determine the effects of mind-body interventions on physical fitness in healthy community dwelling older adults.	Web of Knowledge, PubMed, SPORTDiscus to Nov 2019	2005–2019	I: Yoga, Qi Gong, Tai Chi, Pilates C: Active and inactive controls	30 RCTs	Inclusion: ≥65 Mean: 71.2	2792	37	3% poor 17% fair 63% good 17% excellent (PEDro)
Elboim-Gabyzon 2021 [38]	To explore the effectiveness of high-intensity interval training for reducing fall risk factors in older adults.	PubMed, CINAHL, Cochrane, APA PsycInfo, Web of Science, Scopus, PEDro, AgeLine, ClinicalTrials.gov, Google Scholar to July 2021	2015–2020	I: High-intensity exercise (90–95% peak heart rate, 90% maximal oxygen uptake, at least 75% peak work rate) separated by periods of low to moderate-intensity or rest (e.g., walking/running, cycling). C: No treatment or other exercise	11 (8 RCTs)	Inclusion: Average ≥ 60 Range: 50–81	328	9% F only, 36% M only	45% high 36% moderate 9% low 1 not assessed (PEDro)
Fernandez-Arguelles 2015 [39]	To know the effects of dancing as a physical exercise modality on balance, flexibility, gait and muscle strength in older adults.	PubMed, Cochrane Library Plus, PEDro, ScienceDirect, Dialnet, Academic Search Complete, Jan 2000-Jan 2013	2002–2012	I: Dance-based AT, dance and foot tapping or squatting, Turkish folk dance, low impact aerobic dance, Greek traditional dance, ballroom dance, and salsa dancing C: other types of exercise	7 RCTs	Inclusion: > 60 Range: 63.1–82.2	354 Range: 26–97	NR; 43% F only	29% good 71% fair (PEDro)
Fernández-Rodríguez 2021 [40]	To estimate the effectiveness of Pilates on physical performance and risk of falls in older adults.	MEDLINE, Scopus, Web of Science, Physiotherapy Evidence Database, Cochrane Central Register of Controlled Trials to April 2021	2010–2021	I: At least one exercise intervention described as “Pilates” (Mat, machine, or both) C: Habitual or non-exercise	39 RCTs	Inclusion: ≥60 Range: 60–80	1650	62	64% high 36% unclear (Cochrane)
Finger 2015 [41]	To determine whether protein supplements can optimize the effects of RT on muscle mass and strength in an aged population.	MEDLINE, Cochrane Central, EMBASE, LILACS to January 2014	1995–2013	I: RT and protein for ≥6 weeks. Protein supplements ranged from 0.3 to 0.8 g/kg/day (mean 0.46 g/kg/day) or 6 to 40 g/day (mean = 20.7 g/day) or high protein diet C: RT with placebo or no supplement	9 RCTs	Inclusion: ≥60 Range: 61.2–79.2	462 Range: 12–87	NR; 11% F only, 33% M only	Low-moderate risk of bias (PRISMA statement)
Frost 2017 [42]	To evaluate effectiveness of home- and community-based health promotion interventions on functioning and frailty in older people with mild or pre-frailty.	MEDLINE, EMBASE, Scopus, Social Science Citation Index, Science Citation Index Expanded, Cochrane (library, CENTRAL, EPOC), NHS Health Economic Evaluations, DARE, PsycINFO, CINAHL, Bibliomap, Social Care Online, Sociological Abstracts, Applied Social Sciences Index, Jan 1990-May 2016	2000–2015	I: Home- or community-based health promotion interventions (i.e., interventions that enable people to improve or increase control over their health) C: usual care or health education or flexibility training	10 (7 RCTs)	Inclusion: > 60 Range: 72–83	485	NR	Low or unclear risk of bias (Cochrane)
Gade 2018 [43]	To determine the effect of protein or essential amino acid supplementation during RT in older adults.	PubMed, SCOPUS, EMBASE, Cochrane databases to 2017	1994–2016	I: RT plus protein or essential amino acid supplementation or a modified diet with increased protein content for > 5 weeks C: RT with/without non-protein placebo	16 RCTs	Inclusion: > 60 Range: 61–85	1107 Range: 16–179	NR; 13% F only, 25% M only	Good to excellent (PEDro)
Garcia-Hermoso 2020 [44]	To analyze the safety and effectiveness of long-term exercise interventions in older adults.	PubMed, Cochrane CENTRAL, SPORTDiscus to Sept 16, 2019	1991–2019	I: MCT (*n* =45), RT (*n*=24), AT (*n*=19), and Tai Chi (*n*=4). Most used group-based supervised exercise alone (*n*=46) or combined with home-based unsupervised training (*n*=21) C: usual care with or without non-exercise intervention	99 (93 RCTs; 90 RCTs in meta-analysis)	Inclusion: ≥65 Mean: 74.2	28,523	NR; 19% F only, 4% M only	Good (PEDro)
Grässler 2021 [45]	To summarize the effects of endurance, resistance, coordinative, and multimodal exercise interventions on resting heart rate variability and secondary health factors in healthy older adults.	PubMed, Scopus, SPORTDiscus, Ovid, Cochrane Jan 2005-Sept 2020	2005–2020	I: Physical training intervention (endurance, resistance, coordinative, or multimodal training) with a minimum of 4 weeks and 8 training sessions C: NR	13 RCTs and non-RCTs (designs NR)	Inclusion: ≥60 Mean: 67.8	422	31% F only, 8% M only	Mean: 8.88 (SD 2.47)/15 (Tool for the Assessment of Study Quality and reporting in Exercise) Mean: 20 (SD 1.56)/25 (STARDHRV)
Hanach 2019 [46]	To evaluate the effectiveness of dairy proteins on functions associated with sarcopenia in middle-aged and older adults.	PubMed, CINAHL, Web of Science to May 10, 2017	2009–2016	I: Dairy protein supplementation (e.g., whey protein, milk-protein concentrate, casein) or a protein-based dairy product (e.g., ricotta cheese) for ≥12 weeks with/out RT C: usual care, placebo, or regular dairy	14 RCTs	Inclusion: 45–65 Range: 61–81	1424	NR; 7% F only	Moderate-High (Cochrane)
Hortobagyi 2015 [47]	To determine the effects of strength, power, coordination, and MCT on healthy older adults’ gait speed.	PubMed, Web of Knowledge, Cochrane, Jan 1984 to Dec 2014	1993–2014	I: RT or interventions that included 2+ types of exercise in any combination or functional or coordination training C: no exercise	42 (designs NR)	Inclusion ≥65 Mean: 74.2 Range: 64.4–82.7	2495	63	Low (PEDro)
Hou 2019 [48]	To explore whether a combination of protein supplementation with RT is effective in enhancing muscle mass, strength and function in the elderly.	PubMed, MEDLINE, Embase, Jan 2004-May 2018	2004–2018	I: Protein supplements containing leucine, whey protein, casein, lean meat, low-fat milk or related mixture and RT 1–4 times/week C: RT alone	21 RCTs	Inclusion: > 50 Range: 50–91	1249	NR; 38% F only, 14% M only	Moderate certainty (Cochrane)
Howe 2011 [49]	To examine the effects of exercise interventions on balance in older people, ≥60 y, living in the community or institutional care.	Cochrane Specialized Register, CENTRAL, MEDLINE, EMBASE, PEDro, CINAHL, AMED to Feb 2011	1989–2010	I: Interventions designed to improve balance, or RT or MCT or Tai Chi, qi gong, dance and yoga or gait, coordination, and functional exercises C: attention control	94 RCTs and quasi-experimental	Inclusion: ≥60 Range: 60–75	9821	NR; 27% F only, 5% M only	Most unclear risk of bias (Cochrane)
Hurst 2019 [50]	To assess the effects of same session combined exercise on measures of fitness in adults ≥50 y.	PubMed, MEDLINE, Scopus, BIOSIS, Web of Science to July 2018	1991–2018	I: At least one AT and RT group C: no exercise, AT only, or RT only	27 (22 RCTs)	Inclusion: > 50 Mean: 68.8 Range: 54–85	1346	NR; 44% F only, 18% M only	Low or unclear risk of bias (Cochrane)
Hwang 2015 [51]	To examine the benefits to physical health of dance among older adults.	PubMed, Date NR	2004–2013	I: Dance defined as a form of artistic expression through rhythmic movement to music, not including aerobic fitness classes taught to music C: other activity or no activity	18 (10 RCTs)	Inclusion: NR Range: 52–87	Range: 13–97	NR; 44 > 50% F, 28% F only	Moderate (Sackett, Megens and Harris)
Katsoulis 2019 [52]	To investigate the effect of high- vs. low-intensity RT on muscular power in older, healthy, untrained adults.	MEDLINE, Embase, CINAHL, AgeLine, SPORTDiscus, Scopus to Apr 2017	2001–2017	I: Low (≤50% 1RM), moderate or high (≥70% 1RM) intensity power training C: post-intervention vs. pre-intervention	27 RCTs	Inclusion: > 60 Mean: 74.5 Range: 62.7–81.8	549 Range: 5–59	NR; 22% F only, 7% M only	52% high (> 6); remaining fair-good (PEDro)
King 2016 [53]	To synthesize research that tests the effects of aquatic exercise in healthy older adults on functional balance.	Academic Search Complete, AMED, CINHAL, MEDLINE, SPORTDiscus, Date NR	1996–2013	I: Exercise programs in water, with no restriction on depth or temperature of the aquatic environment. Swimming programs were not included C: land exercise or no exercise	13 (6 RCTs)	Inclusion > 60 Mean: 71 Range: 68–80	545 Range: 20–79	NR; 31% F only, 8% M only	46% good; 54% poor-fair (Downs and Black)
Labott 2019 [54]	To examine the effects of exercise training on handgrip strength in healthy community-dwelling older adults ≥60 y.	PubMed, Web of Science, SPORTDiscus to Nov 25, 2018	1995–2018	I: aquatic exercise, walking, flexibility, TRX-training, home-trainer exercise, RT, vibration platform, dance, Tai Chi, exergames, balance training, calisthenics, and MCT C: NR	24 RCTs	Inclusion: ≥60 Mean: 73.3 ± 6.0	3018 Range: 22–1635	NR; 50% F only 4% M only	Fair (PEDro)
Lesinski 2015 [55]	To quantify effects of balance training on balance outcomes and to characterize dose–response in healthy community-dwelling older adults.	PubMed, Web of Science, Jan 1985 to Jan 2015	1994–2014	I: Balance training protocol comprising static/dynamic postural stabilization exercises (combined training was excluded) C: no intervention	23 RCTs	Inclusion: ≥65 Range: 66–83	1220 Range: 11–75	NR; 9% F only	74% weak (PEDro)
Leung 2011 [56]	To assess the usefulness of tai chi to improve balance reduce falls in older adults.	CINAHL, Science citation index, social science citation index, MEDLINE, Cochrane Central, ScienceDirect, PubMed, Allied & Complementary medicine, China journals, eCAM, Jan 1, 1998-Jan 31, 2008	2000–2007	I: Various styles of Tai Chi C: no treatment or other exercise	13 RCTs	Inclusion: ≥60 Range: 45–98	2151	NR; 23% mostly F, 8% mostly M, 8% M only	Good to excellent (PEDro)
Levin 2017 [57]	To examine the dual effects of different types of physical training on cognitive and motor tasks in older adults with no known cognitive or motor disabilities or disease	PubMed, Jan 2007-Dec 2016	2008–2016	I: physical training (e.g., balance training, AT, strength training, group sports, etc.) or combined physical and cognitive intervention (dual-task) C: passive control or health education classes and lesser training	19 (17 RCTs)	Inclusion: > 65 Range: 65.5 ± 6.3–81.9 ± 6.3	1226	52	Mostly low (Jadad)
Liberman 2017 [58]	To assess the effects of exercise on muscle strength, body composition, physical function and inflammatory profile in older adults.	PubMed, 2015–2016	2015–2016	I: Any exercise; included RT (*n*=16), AT (*n*=8), AT/RT (*n*=6) and other types (*n*=10) C: no intervention	34 RCTs	Inclusion: > 65 Range: 54.5–92.3	1747	NR	Unclear risk of bias across domains (NICE)
Liu 2010 [59]	To determine whether Tai Chi has an effect on static and dynamic balance, functional performance, muscle strength and flexibility, and subjective measures.	MEDLINE, PubMed; Jan 2000-July 2007	2000–2007	I: Tai Chi C: NR	18 (15 RCTs)	Inclusion: ≥60 Mean: NR	3741 Range: 17–1200	NR	NR; lower-quality studies screened out
Liu 2017 [60]	To compare RT or MCT to no intervention or attentional controls, on muscle strength, physical functioning, ADL, and falls in community- dwelling older adults with reduced physical capacity.	MEDLINE, Embase, Cochrane Library Central, Date NR	1996–2015	I: Progressive RT, strength training in which one exerts an effort against an external resistance or MCT combines > 2 types of exercise RT, balance, stretching, and AT C: no intervention or attention control	23 RCTs	Inclusion: ≥60 Mean: 75 Range: 69–84	2018	NR; 22% F only, 4% M only	Low risk of bias (Cochrane)
Liu 2020 [61]	To evaluate the effects of dance on physical function performance in healthy older adults.	Cochrane Library, PsycINFO, PubMed, Scopus, Web of Science to June 2018	2008–2017	I: Dance interventions of at least 6 weeks duration C: usual care with no intervention or other exercise	13 RCTs	Inclusion: ≥65 Mean: NR	1029 Range: 23–510	85	Low-moderate risk of bias (Cochrane)
Loureiro 2021 [62]	To determine the effects of multifactorial programs including physical activity based on individual assessment of fall risk factors on rate of falls and physical performance in older adults.	PubMed, Cochrane Plus, Web of Science, SCOPUS, 2009–2020	2009–2017	I: Multi-component interventions including strength and balance training, flexibility, endurance, gait, and/or functional exercises, treatment of sensory impairments, health education, medical management and/or in home falls risk assessment C: Usual care, delayed intervention, health education	6 RCTs	Inclusion: ≥60 years Mean: 77.62	2012 Range: 19–616	54.4	50% good 50% fair (PEDro)
Martin 2013 [63]	To compare physical therapist–administered group-based exercise with individual or no exercise control.	PubMed, CINAHL, Dec 1, 2001-June 7, 2012	2002–2010	I: Physical therapist led or supervised group exercise C: individual physical therapy or no exercise control	10 RCTs	Inclusion: ≥65 Mean: 76.21 Range: 72–81	2293 Range: 32–1090	NR	Good (PEDro)
Martins 2018 [64]	To identify modified Otago Exercise Program delivering methods and analyze their effects on balance, functional ability and self-reported falls.	PubMed, PEDro, ScienceDirect, Scopus, Date NR	2011–2016	I: Modified Otago Exercise program (RT, balance and walking) C: original Otago program, non-intervention, or other exercise	8 (5 RCTs)	Inclusion: NR Mean: 76.75 ± 5.5	604	NR; 13% F only	Fair-Good (PEDro)
Meereis-Lemos 2019 [65]	To determine the effectiveness of RT and MCT on functionality of healthy older patients.	PubMed, Web of Science, PEDro, Cochrane, Lilacs databases, Date NR	2007–2016	I: Supervised RT or RT combined with another modality at least twice a week for a minimum of 8 weeks C: no exercise	28 RCTs	Inclusion: ≥60 Range: 62.2 ± 4.3–83.4 ± 2.8	NR	NR; 36% F only, 14% M only	Good (PEDro)
Montero 2016 [66]	To explore the effects of AT on VO_2_max, Qmax and Ca-VO_2_max in healthy middle-aged and older subjects.	MEDLINE, Scopus and Web of Science to May 2015	1989–2014	I: Dynamic exercise involving a large muscle mass (e.g., running, cycling), 3 weeks or more C: post-intervention vs. pre-intervention	16 (designs NR)	Inclusion: > 40 Range: 42–71	153	NR; 19% F only, 63% M only	Moderate-high (SAQOR)
Moore 2016 [67]	To assess the effectiveness of community-based interventions to increase physical activity in older people (≥ 65 y) living rural or regional areas.	CINHAL, Ageline, ProQuest Central, PubMed, Informit Complete, Google Scholar to Aug 2014	1997–2014	I: Community-based PA intervention of six weeks or more (from start to follow-up) C: NR	7 (3 RCTs)	Inclusion: ≥65 Mean: NR	Range: 37–1200	NR; 14% F only	High risk of bias (Cochrane)
Moran 2018 [68]	To determine the effect of jump training on muscular power in older adults (≥ 50 y).	Google Scholar, PubMed, Microsoft Academic, Date NR	1998–2018	I: Jump training, defined as lower body unilateral and bilateral bounds, jumps and hops C: NR	9 (designs NR)	Inclusion: ≥50 Range: 53.0–72.4	467	NR; 56% F only, 11% M only	Good (PEDro)
Nicolson 2021 [69]	To evaluate the effects of therapeutic exercise interventions on physical function, health-related quality of life and psychosocial outcomes in community-dwelling adults.	MEDLINE, EMBASE, CINAHL to July 2020	1997–2020	I: Therapeutic exercise including AT, RT, functional training, balance training, gait training, flexibility, or 3D (constant movement in a controlled, fluid, repetitive way through all three spatial dimensions, e.g., Tai Chi) C: Usual care, no treatment, other exercise, pharmacotherapy, or health education	16 RCTs	Inclusion: ≥80 Median: 84.2 (interquartile range: 83.4–86.1)	1660	19% F only, 6% M only	6% low 63% moderate 31% high (Cochrane)
Plummer 2015 [70]	To examine the effects of physical exercise on dual-task performance during walking in older adults.	PubMed, CINAHL, EMBASE, Web of Science, PsycINFO up to Sept 19, 2014	2006–2014	I: Any physical exercise intervention C: active, education, or inactive no treatment/delayed treatment	21 (15 RCTs)	Inclusion: ≥60 Range: 71.1–91.1	Range: 10–134	≥70% in all but 2 studies	Good (Downs and Black)
Qi 2020 [71]	To evaluate the effects of Tai Chi with RT on health outcomes in adults ≥50 y.	PubMed, Scopus, Web of Science, CINAHL, MEDLINE, PEDro, Cochrane library to Jan 2018	2005–2016	I: Tai Chi combined with RT C: any control or comparison	7 (6 RCTs)	Inclusion: ≥50 Range: 58.5–74.0	703	NR; 14% F only	Fair (PEDro)
Raymond 2013 [72]	To examine the effect of high intensity RT on strength, function, mood, QoL, and adverse events in older adults.	Cochrane Central, MEDLINE, Embase, CINAHL, AMED, AgeLine, PEDro to July 2012	1995–2007	I: Lower limb high intensity progressive RT with or without upper limb or trunk strengthening C: other intensity RT	21 RCTs	Inclusion: ≥65 Range: 60–95	724	NR; 14% F only, 24% M only	Poor to fair (PEDro)
Rodrigues-Krause 2019 [73]	To review the literature on the use of dance to promote functional and metabolic health in older adults.	MEDLINE, Cochrane Wiley, ClinicalTrials.gov; PEDRO, LILACS, Nov 1980 to Mar 2016	1984–2016	I: Regular dance classes of any style for at least 2 weeks. Dance environments included dance studios and stage and/or dance ballrooms C: inactive control or other exercise	50 (31 RCTs)	Inclusion: > 55 Range: 50–94	Range: 10–700	NR; 34% F only, 4% M only	Majority high risk of bias (PRISMA)
Roland 2011 [74]	To investigate whether physical fitness and function benefits are engendered through the practice of yoga in older adults.	PubMed, Scholars Portal, AgeLine, CINAHL, EBSCO, MEDLINE, SPORTDiscus, PsycINFO, EMBASE, 1970–2009	1989–2009	I: Yoga C: other exercise, no exercise, or pre/post yoga groups	10 (5 RCTs)	Inclusion: ≥65 or 55–64 Mean: 69.6 ± 6.3	544 Range: 13–176	71	Moderate-high (Modified Downs and Black)
Sivaramakrishnan 2019 [75]	To synthesize existing evidence on the effects of yoga on physical function and QoL in older adults not characterized by any specific clinical condition.	MEDLINE, PsycINFO, CINAHL Plus, Scopus, Web of Science, Cochrane Library, Embase, SPORTDiscus, AMED, ProQuest Dissertations & Theses Global to Sept 2017	1983–2017	I: Yoga C: inactive or active controls	22 RCTs	Inclusion: ≥60 Range: 61.0–83.8	Range: 18–410	> 70	Moderate risk of bias (Cochrane)
Stares 2020 [76]	To assess whether creatine combined with exercise results improves indices of skeletal muscle, bone, and mental health over exercise alone in healthy older adults.	PubMed, CINAHL, Web of Science, 1998–2018	1998–2016	I: A physical training program and creatine supplementation C: placebo	17 RCTs	Inclusion: ≥48 Mean age: NR Range: 48–84	583	39	Overall good (PEDro)
Stathokostas 2012 [77]	To assess the effects of flexibility training on functional outcomes in healthy older adults > 65 y.	PubMed, Embase, CINAHL, Scopus, and SPORTDiscus to Jan 2011	1988–2011	I: Flexibility training (excluding Tai Chi or yoga) C: NR	22 (13 RCTs)	Inclusion: ≥65 Mean: 74.1 Range: 64–88.8	1127 Range: 7–132	75	RCTs: good Non-RCTs: low-moderate (Modified Downs and Black)
Straight 2016 [78]	To estimate the effect of RT on lower-extremity muscle power in middle-aged and older adults.	Google Scholar to Nov 1, 2014	1995–2013	I: RT, defined as muscle-strengthening activities that use major muscle groups and could include free weights, machines, and resistance bands C: usual care or sham exercise	12 RCTs	Inclusion: ≥50 Range: 56.3–93 (intervention), 56.7–93 (control)	810	NR; 17% F only, 8% M only	NR
Ten Haaf 2018 [79]	To assess the effect of protein on lean body mass, muscle strength, and/or physical performance, in non-frail community-dwelling older adults.	PubMed, Embase, Web of Science to May 15, 2018	1992–2018	I: Multi-nutrient protein or essential amino acid supplementation added to or replacing normal diet with or without RT. Supplements were consumed ≥3 times/week for at least 4 weeks C: placebo control or RT	36 RCTs	Inclusion: ≥50 Range: 55–85	1682	NR; 19% F only, 31% M only	50% Moderate, 42% Good, 8% Excellent (Downs and Black)
Tschopp 2011 [80]	To determine the effects of power training with high movement velocity for older community-dwelling people.	PubMed (MEDLINE), EMBASE, CINAHL, PEDro, Cochrane Central and Google Scholar to April 2010	2002–2009	I: Power training (training with moderate resistance and an ‘as fast as possible’ movement speed for at least the concentric phase of an exercise) C: Conventional RT (high or moderate resistance and slow concentric movement)	11 RCTs	Inclusion: > 60	377	NR	Moderate risk of bias (Tool NR)
Van Abbema 2015 [81]	To determine the effects of different types or combinations of exercise to improve preferred gait speed.	PubMed, EMBASE, AMED, CINAHL, ERIC, MEDLINE, PsycINFO, SocINDEX, and Cochrane Library 1990 -Dec 9, 2013	1994–2013	I: Progressive RT or RT, balance and AT with or without additional training components, exercise interventions with a dance/rhythmic component or stretching exercises C: usual care or attention control	25 RCTs	Inclusion: ≥65 Mean: 75.8 Range: 61.4 ± 5.5–87.1 ± 0.6	2389	NR; 32% F only	Low-quality studies excluded Moderate-high (PEDro)
Vetrovsky 2019 [82]	To evaluate the safety and efficacy of plyometric training in older adults regarding various performance, functional, and health related outcomes.	PubMed, SPORTDiscus, Scopus, and EMBASE to 2017	2007–2017	I: Plyometric training (eccentric loading followed by a concentric contraction, e.g., repetitive jumping, hopping, bounding, and skipping) or MCT with plyometrics C: non-exercising control or other exercise	12 RCTs	Inclusion: ≥60 Range: 58.4–79.4	289 Range: 8–36	61	75% high (PEDro)
Waller 2016 [83]	To investigate the effect of aquatic exercise on physical functioning in healthy older adults.	MEDLINE, Embase, CINAHL, PEDro, SPORTDiscus, Web of Science, Cochrane Library to Dec 31, 2015	1994–2015	I: Exercise in an aquatic environment with no limitation on the type of exercise C: land exercise or no exercise	28 RCTs	Inclusion: ≥55 Mean: 66.4 Range: 55.4–82.0	1456	89	High risk of bias (Cochrane)
Wang 2021 [84]	To examine the impact of Traditional Chinese medicine-based exercises on physical performance, balance, and muscle strength in the elderly.	PubMed, EMBASE, Scopus, Cochrane Central Register of Controlled Trials, China National Knowledge Infrastructure, Wan Fang, manual search of Soochow University and Nanjing University of Chinese Medicine libraries to March 2021	2003–2020	I: Traditional Chinese medicine-based exercises including but not limited to Tai Chi, Ba Duan Jin, and Qigong C: Placebo, AT, routine care, or educational programs	27 RCTs	Inclusion: ≥55 Range: 59.7–88.8	2580	68	Moderate (Cochrane)
Wirth 2020 [85]	To investigate the effect of protein supplementation on body composition and muscle function in healthy adults.	PubMed, Web of Science, CINAHL, Embase to March 2019	2001–2019	I: Oral protein intake, 2wk minimal duration, including energy-restriction or not, and including exercise or not C: Low-protein diet, no protein supplementation, or non-protein placebo	23 RCTs	Inclusion: > 55 Range: 55–81	1290	62	Moderate certainty (GRADE)
Yang 2019 [86]	To determine intensity and interval of effective interventions in improving physical function in community-dwelling older adults.	PubMed, EBSCO, and Cochrane Trials, Jan 1, 2013-Dec 31, 2017.	2013–2017	I: Any types of MCT interventions that were conducted in the community, delivered by any kinds of providers C: no exercise control	5 RCTs	Inclusion: > 60 Mean: 70 (intervention) 69 (control)	272	“Majority female”	Moderate (Cochrane)

*F* Female, *M* Male, *NR* Not reported, *RCT* Randomized controlled trial, *PEDro* Physiotherapy Evidence Database, *GRADE* Grading of Recommendations, Assessment, Development and Evaluations, *MCT* Multicomponent interventions, *AT* Aerobic exercise training, *RT* Resistance training, *QoL* Quality of life, *ADL* Activities of daily living, *VO*_*2*_ *max* Maximal oxygen consumption, *Qmax* Maximal cardiac output, *Ca-VO*_*2*_*max* Arteriovenous oxygen difference at maximal exercise

Included reviews reported on several types of interventions, with some reporting separate results for more than one intervention type. Most reviews focused on exercise or PA only (*n* = 53) [26–35, 37–40, 42, 44, 45, 47, 49–75, 77, 78, 80–84, 86], while others included exercise with nutritional supplements (*n* = 9) [25, 36, 41, 43, 46, 48, 76, 79, 85]. No reviews included group-based nutrition interventions alone. Exercise or PA interventions were categorized as resistance exercise (*n* = 12) [26, 35, 47, 49, 52, 60, 65, 69, 72, 78, 80, 81], aerobic exercise (*n* = 5) [28, 29, 33, 38, 66], combined aerobic and resistance exercise (*n* = 9) [27, 34, 47, 50, 57, 60, 64, 65, 81], general PA (*n* = 12) [42, 44, 45, 49, 54, 58, 62, 63, 67, 69, 70, 86], mind-body exercise (e.g., Tai Chi, yoga, Pilates) (*n* = 11) [31, 32, 37, 40, 49, 56, 59, 71, 74, 75, 84], dance (*n* = 5) [39, 51, 61, 73, 81], and other (e.g., aquatics, stretching) (*n* = 10) [30, 47, 49, 53, 55, 68, 77, 81–83]. Nutritional supplements included protein (*n* = 5) [41, 43, 48, 79, 85], creatine (*n* = 2) [36, 76], vitamin D (*n* = 1) [25], or dairy (*n* = 1) [46]. Meta-analyses were undertaken in 39 reviews [25, 26, 29, 31–37, 40–42, 44, 46–50, 54–56, 60, 61, 65, 66, 68–70, 72, 75, 78–81, 83–86], 22 reviews presented findings narratively [27, 28, 38, 39, 43, 45, 51–53, 57–59, 62–64, 67, 71, 73, 74, 76, 77, 82], and one performed a network meta-analysis [30]. Total sample sizes ranged from 153 to 28,523 when reported. Participants ranged from 42 to 98 years old, with most reviews only including studies with participants aged 60 and older. No reviews extracted data on material deprivation, low income, or immigrant populations.

Eligible reviews included 1339 primary studies, of which 962 were unique (28.2% overlap across reviews, although some duplicates were included in reviews focused on different intervention types). Reviews with the most overlap by intervention type were exercise with nutritional supplements (36.2% overlap), dance (31.9% overlap), and resistance exercise (26.3% overlap). Single studies were published between 1983 and 2021 (range 5 to 99 studies per review). Of these, 83% were randomized controlled trials and 17% were quasi-experimental, observational, or not reported.

### Methodological quality of included reviews

**Fig. 2:**
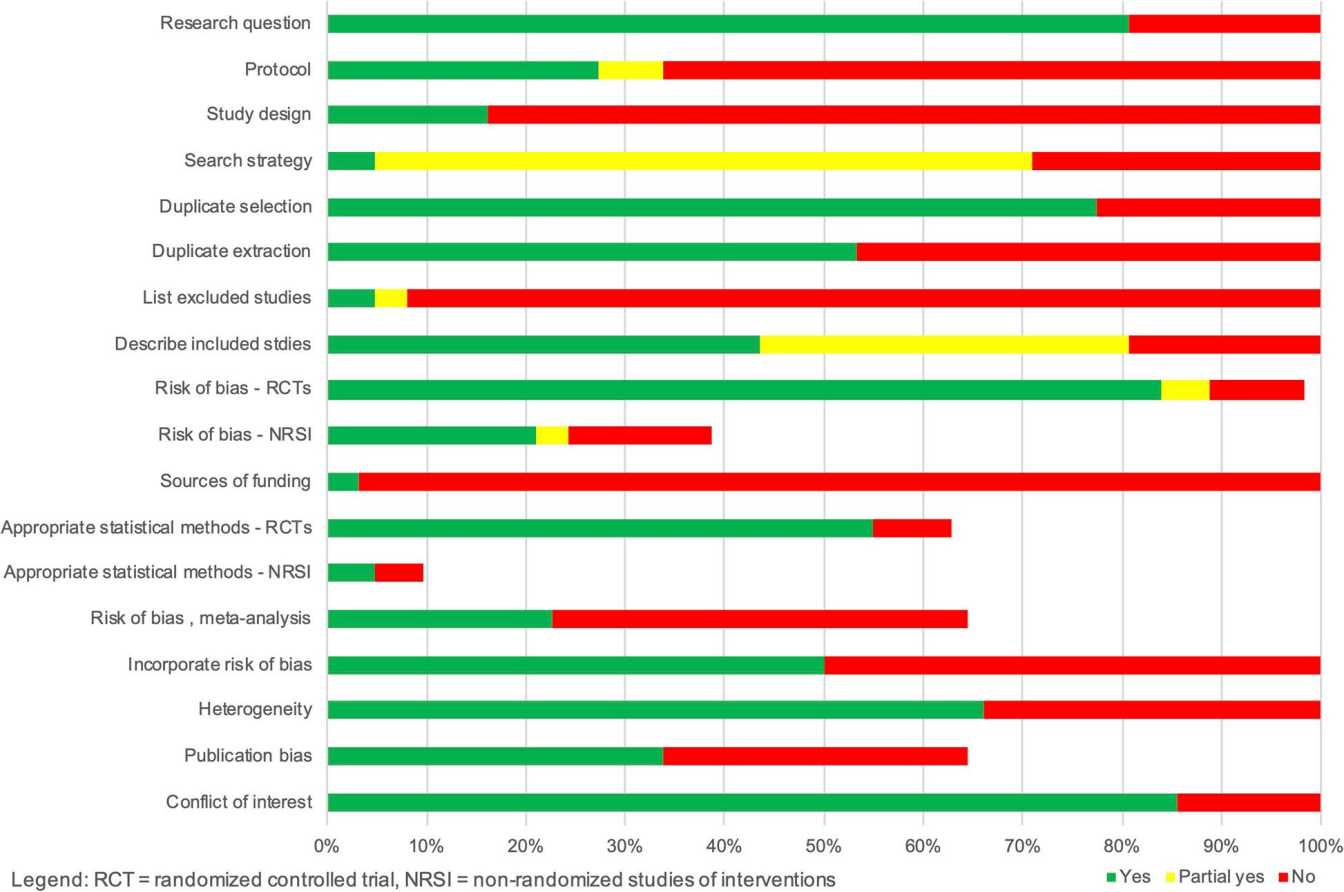
AMSTAR 2 Summary of Systematic Review Quality. Legend: RCT = randomized controlled trial, NRSI = non-randomized studies of interventions

Methodological quality of the reviews was variable (summary in Fig. 2, full assessment in Additional file 3), with one review [49] rated as having high confidence in findings. The confidence for the remaining reviews were moderate (*n* = 21) [25, 28, 30, 35, 36, 42–44, 46, 50, 53, 66, 67, 71, 72, 75, 77, 80–83], low (*n* = 17) [31, 34, 39, 40, 48, 54–56, 60, 63, 69, 70, 73, 74, 76, 85, 86], and critically low (*n* = 23) [26, 27, 29, 32, 33, 37, 38, 41, 45, 47, 51, 52, 57–59, 61, 62, 64, 65, 68, 78, 79, 84]. Most reviews did not report protocol registration, describe an adequate search strategy, justify excluded studies, or incorporate risk of bias in interpreting review findings.

### Findings of reviews

**Fig. 3:**
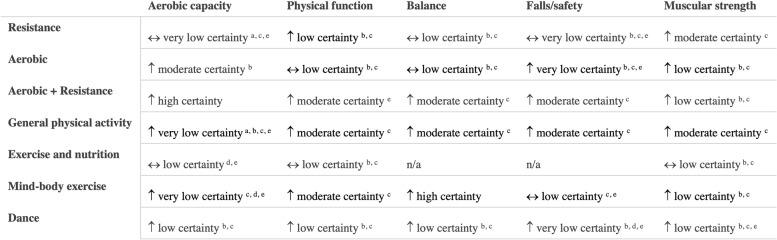
GRADE Summary of Certainty of Evidence. Legend: ^a^ start at low certainty due to non-randomized study designs. ^b^ downgraded due to risk of bias. ^c^ downgraded due to inconsistency in effects. ^d^ downgraded due to indirectness of interventions/outcomes. ^e^ downgraded due to imprecision in effect estimate. ^f^ downgraded due to publication bias. ^g^ upgraded due to large effect. ^h^ upgraded due to dose-response relationship. ^i^ upgraded due to accounting for confounding

A summary of findings by intervention type and outcome category, alongside review quality is listed in Table 2, with a summary of the certainty of evidence (GRADE) in Fig. 3.

**Table 2:**
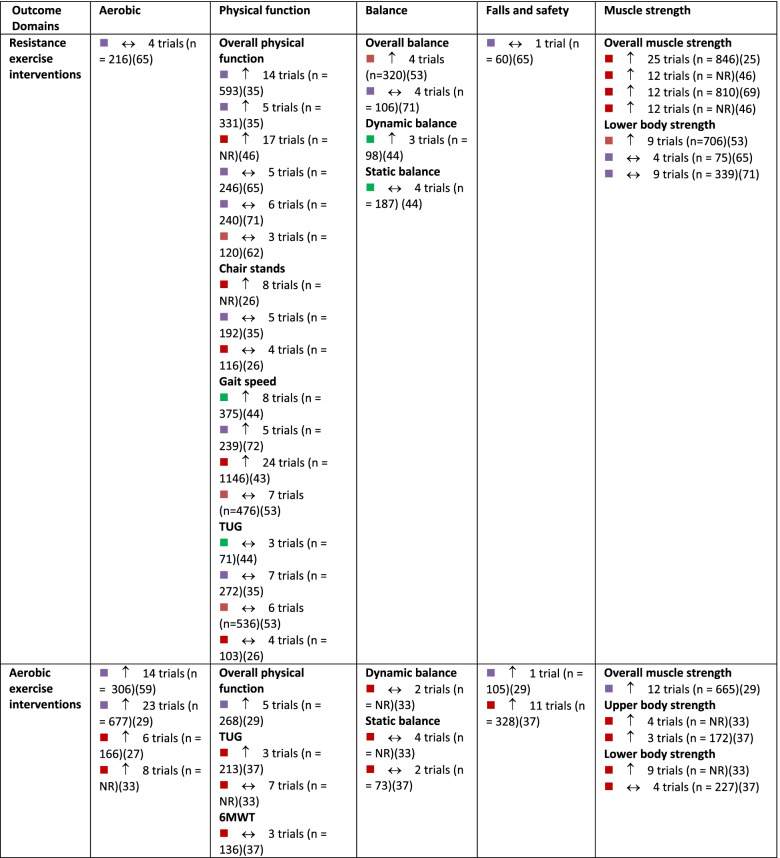

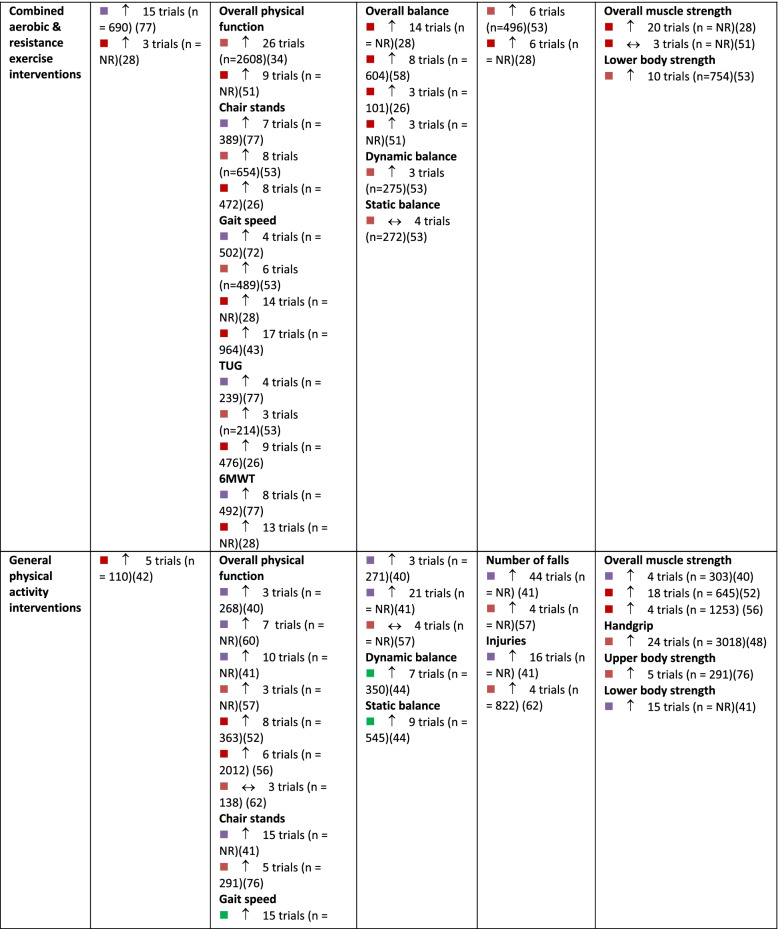

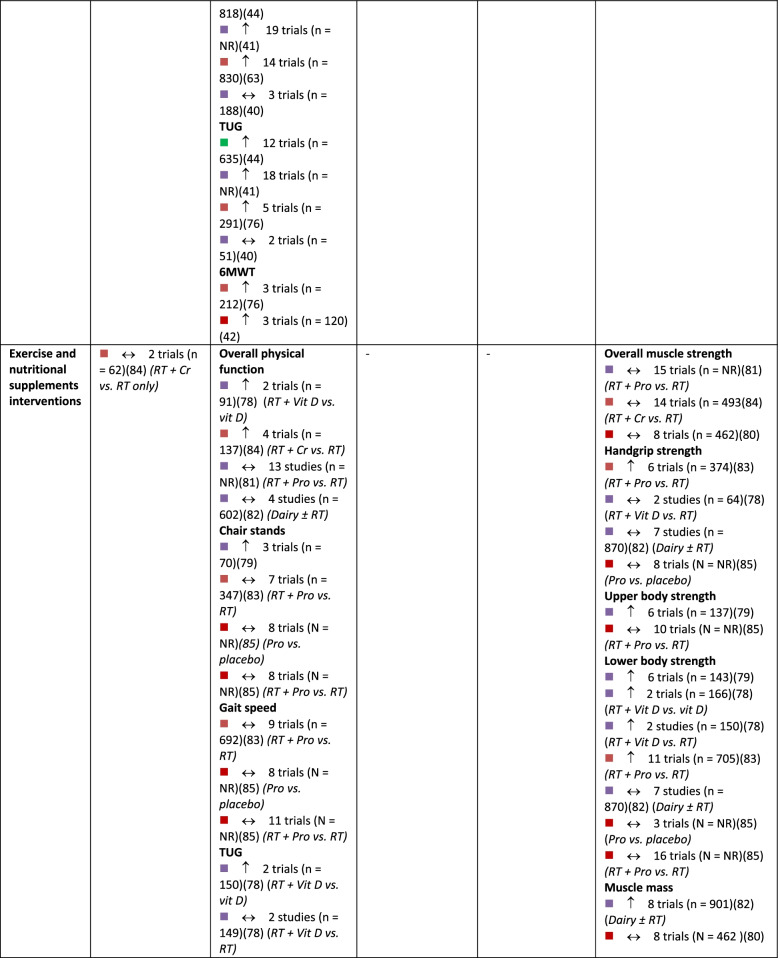

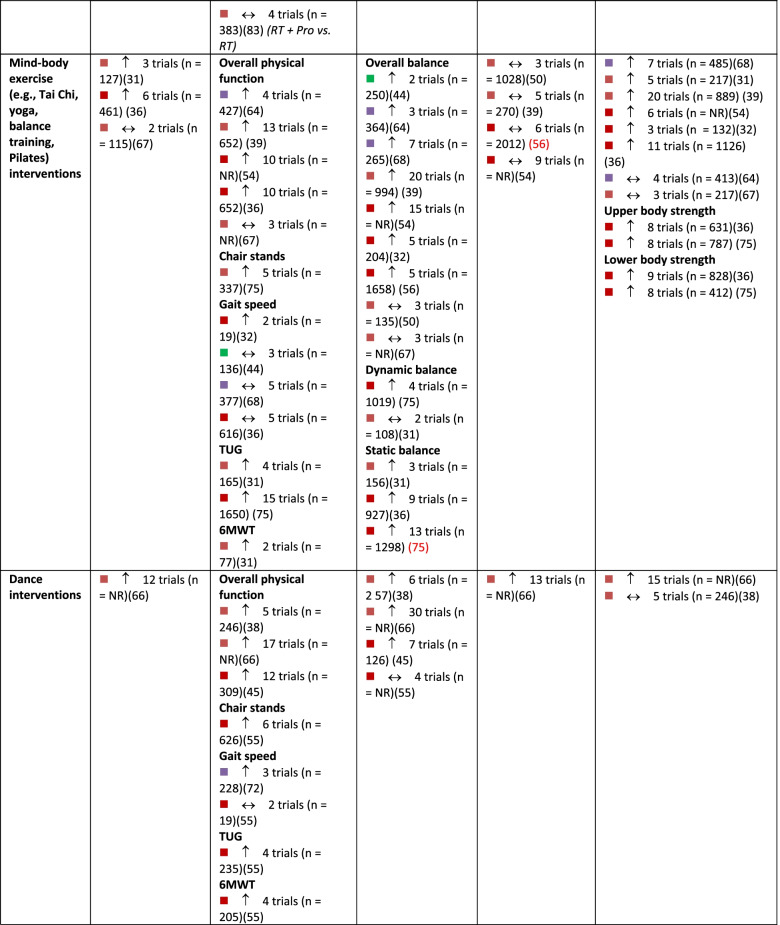

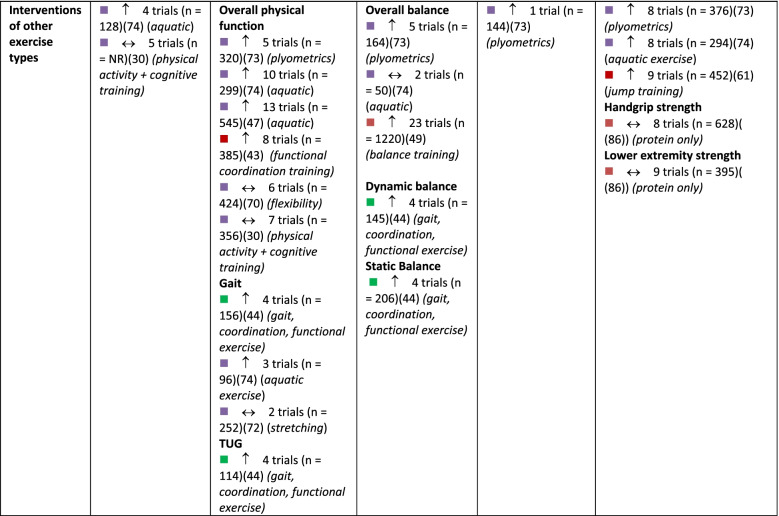
Summary of results across included systematic reviews by outcome domain and intervention type, alongside methodological quality of the review, number of trials included in the relevant analysis and sample size

Note:  = critically low quality;  = low quality;  = moderate quality;  = high quality (as determined by AMSTAR 2).  = compared to a control group, intervention demonstrates statistically significant beneficial effect on outcome;  = intervention demonstrates no significant change in outcome when compared to a control group.

*6MWT *six-minute walking test, *CR* creatine supplementation, *NR* not reported, *Pro *protein supplementation, *RT *resistance training, *TUG* Timed Up and Go test, *Vit D *vitamin D supplementation

#### Aerobic capacity

The effect of interventions on aerobic capacity was reported in 15 reviews [27–31, 33, 37, 45, 50, 66, 72–74, 76, 83] (Additional file 4). Across reviews, 109 studies were reported, of which 104 were unique (overlap, 4.6% across intervention types). Aerobic capacity was most often assessed using measured or predicted maximal or peak oxygen consumption (VO_2_ peak). Based on high-certainty evidence, a combination of aerobic and resistance training results in meaningful improvements in aerobic capacity in older adults. Based on moderate certainty evidence, aerobic exercise probably results in improvements in aerobic capacity. Dance interventions may result in increased aerobic capacity, although this is based on low-certainty evidence and findings may change as more information becomes available. Interventions that combined exercise with nutritional supplements may make little to no difference in aerobic capacity of older adults, although this is based on low-certainty evidence. The evidence is very uncertain about the effect of mind-body exercise, general physical activity, or resistance exercise alone on aerobic capacity in older adults (very low certainty evidence). Other exercise types, including aquatic exercise and a combination of PA and cognitive training, were also examined for their effect on aerobic capacity.

#### Physical function

In total, 51 reviews [25, 27, 28, 30–40, 42–53, 57–63, 65, 67, 69–77, 79–84, 86] reported on 596 single studies, of which 434 were unique (27.2% overlap across intervention types). Physical function was typically assessed using the Timed Up and Go test, chair stands, gait speed, and six-minute walk test; often findings from single studies were compiled into a composite score for self-reported and/or measured physical function within meta-analyses (Additional file 5). Based on moderate certainty evidence, interventions that included a combination of aerobic and resistance exercise, interventions focused on general PA, and mind-body exercise interventions are all likely to result in improvements in physical function in older adults. Resistance training and dance interventions may also increase physical function (low certainty evidence). Low certainty evidence suggests that aerobic exercise interventions and exercise combined with nutritional supplements may have little to no impact on physical function.

#### Balance

In total, 30 reviews [27, 31–33, 37–40, 42, 44, 49, 51, 55–57, 59, 60, 62–65, 71, 73–75, 80, 82–84] reported on 275 single studies, of which 226 were unique (17.8% overlap). Static and dynamic balance tests (e.g., single-leg stance, Berg Balance Scale) and composite balance measures were used across reviews (Additional file 6). High certainty evidence suggests that participation in mind-body exercise interventions increases balance in older adults. General PA interventions and interventions that combined aerobic and resistance training are also likely to result in improvements in balance in older adults, based on moderate-certainty evidence. Dance interventions may improve balance; however, this is based on low certainty evidence. Also based on low certainty evidence, resistance training and aerobic exercise alone may result in little to no change in balance. No included reviews explored the effects of exercise and nutritional supplements on balance.

#### Falls and safety

Number of falls, risk of falling, and fall-related injuries were measured across 14 reviews [27, 28, 38, 40, 44, 56, 59, 60, 62, 63, 69, 72, 73, 82] including 108 single studies, 98 of which were unique (9.3% overlap). Interventions that combine aerobic and resistance exercise and interventions focused on general PA are likely to result in a small reduction in the risk of falls or fall-related injuries in older adults, based on moderate certainty evidence (Additional file 7). Based on low-certainty evidence, mind-body exercises may have little to no meaningful effect on fall risk, although these findings may change as more data are available. Dance interventions and aerobic exercise only may reduce falls, but the evidence is of very low certainty. Also based on very low certainty evidence, resistance training alone may have little to no effect on falls risk. No reviews reported the risk of falls within interventions that combined exercise and nutrition.

#### Muscle strength

Within reviews reporting muscle strength outcomes, 40 reviews [25–28, 31–33, 36–44, 46, 48, 52, 54, 58–60, 62, 65, 68, 71–76, 78–80, 82–86] reported on 452 single studies, of which 349 were unique (22.8% overlap). Various measures were reported, including handgrip strength, upper body strength, lower body strength, muscle mass, and overall muscle strength (Additional file 8). Both resistance exercise interventions and general PA interventions likely increase upper and lower body strength (moderate certainty evidence). Aerobic exercise alone, combined aerobic and resistance exercise, mind-body exercise, and dance interventions may result in improvements in muscle strength, however, this is based on low certainty evidence and findings may change as more data become available. Also based on low certainty evidence, interventions that combined exercise with nutritional supplements may not improve muscle strength.

#### Health-related quality of life and self-reported wellbeing

In total, 14 reviews [27, 28, 32, 33, 38, 42, 44, 60, 61, 63, 72, 73, 75, 82] reported health-related quality of life and self-reported wellbeing outcomes (Additional file 9). Given the variation in constructs measured within this domain (e.g., activities of daily living, quality of life (SF-36), perceived mental health) and limited number of reviews for each outcome type, these results were not incorporated into the overall summary of findings using GRADE.

## Discussion

We provide a high-level comprehensive synthesis regarding the overall effectiveness of group-based PA and/or nutrition interventions to improve mobility among community-dwelling older adults. Within this review, we take a broad view of mobility, which captures several modifiable risk factors that influence older adults’ ability to move within and beyond their immediate environments [1, 5]. Interventions that combined aerobic and resistance exercise, and general PA interventions were found to result in meaningful improvements in physical function, balance, and muscle strength in older adults and are also likely to reduce falls and fall-related injuries. Mind-body exercise is also effective at improving physical function and balance, as is combined aerobic and resistance exercise for aerobic capacity.

Our findings support a multifaceted approach to health and wellbeing among community-dwelling older adults. Similar findings are reflected in two overviews of reviews focused on all adults over the age of 18, including older adults [87, 88], which informed the recent Canadian 24-Hour Movement Guidelines [89]. These guidelines also recommend a combination of aerobic, resistance, and balance exercises for adults aged 65+. Recent evidence has found that older adults face unique barriers and hesitancy to engage in certain types of exercise, such as resistance training [90]. It is encouraging that benefits for each of our outcome domains were seen across a range of intervention types. This suggests that effective interventions for older adults can incorporate a variety of types of exercises or physical activities that are most likely to foster enjoyment. This notion is consistent with emerging literature regarding the role of intrinsic motivation (i.e., enjoyment in physical activity) as an important predictor of physical activity engagement among older adults [91]. This approach can also improve accessibility to PA within this population by building upon existing community services and group-based PA programs that provide the additional benefit of social participation, which enhances enjoyment, adherence, and sustainability of PA [92]. The importance of social participation for older adults is supported by recent research informed by social-cognitive and socio-emotional theories, suggesting that older adults experiencing social isolation may derive meaningful social benefits from interactions with other participants in group-based exercise programs [93].

Our findings did not provide any convincing evidence for the addition of protein, creatine, vitamin D, or dairy supplementation to PA interventions to improve mobility-related outcomes within community-dwelling older adults. However, the overall quality of the systematic reviews and single studies was low to moderate, and numerous distinct comparator groups were used to test intervention effectiveness. These reviews typically synthesized highly heterogeneous single studies, including a wide range in “dose” of both exercise and dietary supplement components of the interventions; this may have limited the ability to see effects of specific combinations of interventions when synthesized together. Future high-quality studies with similar intervention and comparator groups may provide a better understanding of the role of combined diet and nutrition interventions on mobility-related outcomes in older adults. No reviews focused on group-based nutrition interventions alone, nor did any explore or report on domains of equity, diversity, and inclusion, highlighting priorities for future research.

There are several inherent limitations of this umbrella review that should be considered in interpreting results. Included reviews were limited to those in English, published since 2010. Considering the redundancy in single studies across the reviews dating back as early as 1983, we feel our strategy is robust, captures relevant data from much earlier than 2010, and conclusions are highly unlikely to be changed by older studies that employed less relevant methodologies and practices compared to those used today. Given the large number of included reviews, the overlap in single studies across reviews is unsurprising. The highest amount of overlap of studies evaluating physical function outcomes is attributable to our broad characterization of this outcome and the overlap in single studies among reviews focused on resistance, exercise and nutrition, and dance interventions. Although 28.2% overlap in single studies exists, each review contributing to these results focused on specific outcomes (e.g., gait speed alone, composite physical function measures), and we do not anticipate this greatly influenced our overall certainty of evidence. At the systematic review level, it was not possible to extract specific intervention “doses” and we did not examine single studies to collect this data. Although we would expect targeted aerobic, resistance, or combined aerobic and resistance exercise to be more effective than general PA interventions, certainty in the evidence was influenced by higher risk of bias and heterogeneity across both single studies and reviews of aerobic, resistance, and combined interventions, reflective of variation in types of interventions and tools used to assess outcomes. Finally, changing behaviour is a necessary precursor to changes in mobility-related health outcomes. For example, if an intervention fails to increase physical activity levels of older adults, an improvement in cardiovascular fitness or muscular strength will not occur. An understanding of interventions or techniques that are most effective to change older adults’ physical activity and/or nutrition-related behaviour is an important area of study, particularly when considering sustainability of change beyond the research study. A synthesis of the literature to answer this question is warranted but is outside of the scope of this review.

A strength of this umbrella review was the collaboration with older adults and service provider partners to inform the protocol and identify relevant outcomes. Specifically, the older adult partners involved in this project prioritized the inclusion of quality of life and wellbeing as outcomes of primary importance. The partners voiced that older adults’ self-reported functional measures were likely more meaningful to older adults than measures designed to capture physiology or function. We recognize that objective measures are important as benchmarks; however, we propose that subjective ratings represent a personal participant-relevant domain that could be as, or more, important when considering intervention effectiveness. However, very few reviews reported these outcomes separately as they were commonly combined within meta-analyses, thus we are unable to distinguish between self-reported and objectively measured function.

## Conclusion

Group and community-based PA interventions that combine aerobic and resistance, general PA, and mind-body exercise can improve mobility measures in older adults. There was no evidence of benefit for nutritional supplementation with physical activity. No reviews focused on group-based nutrition interventions alone, and very few identified quality of life outcomes, highlighting a need for future synthesis work. The results of this umbrella review will be used to inform the co-design of a community-based, mobility-enhancing intervention.

## Supplementary Information

**Additional file 1.** Search Strategy.

**Additional file 2.** List of Excluded Studies.

**Additional file 3.** AMSTAR 2 Critical Appraisal Results.

**Additional file 4.** Aerobic Outcomes.

**Additional file 5.** Physical Function Outcomes.

**Additional file 6.** Balance Outcomes.

**Additional file 7.** Falls and Safety Outcomes.

**Additional file 8.** Muscle Strength Outcomes.

**Additional file 9.** Health-Related Quality of Life and Wellbeing Outcomes.

**Additional file 10.** PRISMA Systematic Reviews and Meta-Analyses Checklist.

## Data Availability

The datasets supporting the conclusions of this article are included within the article and its additional files.

## Abbreviations

AMSTAR 2: A MeaSurement Tool to Assess systematic Reviews
EMBOLDEN: Enhancing physical and community MoBility in OLDEr adults with health inequities using commuNity co-design trial
GRADE: Grading of Recommendations Assessment, Development and Evaluation
JBI: Joanna Briggs Institute
PA: Physical activity
RCT: Randomized controlled trial
