# Supplementary material for: Community-based group physical activity and/or nutrition interventions to promote mobility in older adults: an umbrella review
Source: BMC Geriatr. 2022 Jun 29;22:539. doi: 10.1186/s12877-022-03170-9 (PMC9241281; doi:10.1186/s12877-022-03170-9)
Supplement: Supplementary file 1 — Additional file 1. Search Strategy. [file 12877_2022_3170_MOESM1_ESM.docx]

**Additional file 1: Search Strategy**

**Database: OVID Medline Epub Ahead of Print, In-Process & Other Non-Indexed Citations, Ovid MEDLINE(R) Daily and Ovid MEDLINE(R) 1946 to Present**

1. exp nutrition therapy/
2. nutrition$.tw.
3. exp diet/
4. (eat or eating).tw.
5. (diet? or dietary).tw.
6. exercis$.tw.
7. exp exercise therapy/
8. exp exercise/
9. physical activit$.tw.
10. mobility limitation/
11. (mobility or mobilization or mobilisation or mobile).tw.
12. abilit$.tw.
13. function$.tw.
14. adl?.tw.
15. "activities of daily living".tw.
16. exp aged/ or middle aged/
17. (older adult$ or elderly or 55 years or senior?).tw.
18. intervention$.tw.
19. program$.tw.
20. (therapy or therapies).tw.
21. (therapy or pc).fs.
22. (comparative study or controlled clinical trial or meta analysis or multicenter study or pragmatic clinical trial or randomized controlled trial or clinical trial).pt.
23. cohort studies/ or cohort$.tw.
24. (random$ or study or studies or trial or trials).tw.
25. animals/ not (animals/ and humans/)
26. or/1-9
27. or/10-15
28. 16 or 17
29. or/18-21
30. or/22-24
31. 26 and 27 and 28 and 29 and 30
32. animals/ not (animals/ and human/)
33. 31 not 32
34. limit 33 to english language

**Database: Embase**

1. nutrition/ or exp diet/ or exp dietary intake/ or geriatric nutrition/ or nutrition education/ or nutritional assessment/ or nutritional counseling/ or nutritional health/
2. nutrition$.tw.
3. (eat or eating).tw.
4. (diet? or dietary).tw.
5. exp exercise/ or exercis$.tw. or physical activit$.tw.
6. physical activity/ or cycling/ or jogging/ or running/ or stretching/ or swimming/ or walking/ or weight bearing/ or weight lifting/
7. limited mobility/
8. daily life activity/
9. (mobility or mobilization or mobilisation or mobile).tw.
10. function$.tw.
11. adl?.tw.
12. "activities of daily living".tw.
13. or/1-6
14. or/7-12
15. 13 and 14
16. adult/ or exp aged/ or middle aged/
17. (older adult$ or elderly or 55 years or senior?).tw.
18. 16 or 17
19. health program/
20. exp body weight management/
21. intervention$.tw.
22. program$.tw.
23. (therapy or therapies).tw.
24. (therapy or tu or prevention).fs.
25. or/19-24
26. clinical study/ or exp clinical trial/ or community trial/ or intervention study/ or exp longitudinal study/ or major clinical study/ or prospective study/
27. (random$ or study or studies or trial or trials or cohort$).tw.
28. controlled clinical trial/
29. or/26-28
30. 15 and 18 and 25 and 29
31. limit 30 to (human and english language)
32. limit 31 to conference abstract status
33. 31 not 32

**Database: Cochrane CENTRAL**

1. MeSH descriptor: [Nutrition Therapy] explode all trees
2. MeSH descriptor: [Diet] explode all trees
3. MeSH descriptor: [Exercise Therapy] explode all trees
4. (nutrition or eat or eating or diet* or exercis* or physical activ*):ti,ab,kw (Word variations have been searched)
5. #1or#2or#3or#4
6. MeSH descriptor: [Mobility Limitation] this term only
7. mobility or mobilization or mobilisation or mobile or function* or abilit* or "activities of daily living" or adl or adls
8. #6 or #7
9. #5 AND #8
10. MeSH descriptor: [Aged] explode all trees
11. MeSH descriptor: [Middle Aged] this term only
12. olderadult$orelderlyor55yearsorsenior*
13. #10or#11or#12
14. #9and#13

**Database: Sociological Abstracts**

ti(((older adult? OR elderly OR 55 years OR senior?) AND (nutrition* OR eat OR eating OR diet? OR dietary OR exercis* OR physical activit* OR mobility OR mobilization OR mobilisation OR mobile OR abilit* OR adl? OR function* OR "activities of daily living") AND (therapy OR therapies OR intervention? OR programme* OR cohort? OR random* OR study OR studies OR trial OR trials))) OR ab(((older adult? OR elderly OR 55 years OR senior?) AND (nutrition* OR eat OR eating OR diet? OR dietary OR exercis* OR physical activit* OR mobility OR mobilization OR mobilisation OR mobile OR abilit* OR adl? OR function* OR "activities of daily living") AND (therapy OR therapies OR intervention? OR programme* OR cohort? OR random* OR study OR studies OR trial OR trials)))

**Database: CINAHL**

S1 (MH "Nutrition") OR (MH "Diet+") OR (MH "Geriatric Nutrition")

S2 (MH "Exercise+")

S3 (MH "Diet Therapy+")

S4 TI(eatoreating)OR AB(eatoreating)

S5 TI ( diet? or dietary ) OR AB ( diet? or dietary )

S6 TI exercis* OR AB exercis*

S7 TI nutrition* OR AB nutrition*

S8 TI physical activit* OR AB physical activit*

S9 (MH "Functional Status")

S10 (MH "Physical Mobility") OR (MH "Movement")

S11 (MH "Activities of Daily Living")

S12 TI ( "activities of daily living" or adl or adls ) OR AB ( "activities of daily living" or adl or adls )

S13 TI ( mobility or mobilization or mobilisation or mobile ) OR AB ( mobility or mobilization or mobilisation or mobile )

S14 TI function* OR AB function*

S15 (MH "Aged") OR (MH "Aged, 80 and Over+") OR (MH "Frail Elderly")

S16 (MH "Middle Age")

S17 TI ( older adult* or elderly or 55 years or senior? ) OR AB ( older adult* or elderly or 55 years or senior? )

S18 (MH "Clinical Trials+")

S19 (MH "Prospective Studies") OR (MH "Concurrent Prospective Studies")

S20 (MH "Multicenter Studies") OR (MH "Comparative Studies+")

S21 TI ( random* or study or studies or trial or trials ) OR AB ( random* or study or studies or trial or trials )

S22 TI cohort* OR AB cohort*

S23 TI ( intervention* or therapy or therapies or program* ) OR AB ( intervention* or therapy or therapies or program* )

S24 S9 OR S10 OR S11 OR S12 OR S13 OR S14

S25 S15 OR S16 OR S17

S26 S18 OR S19 OR S20 OR S21 OR S22 OR S23

S27 S1 OR S2 OR S3 OR S4 OR S5 OR S6 OR S7 OR S8

S28 S24 AND S25 AND S26 AND S27
