# Supplementary material for: Community-based group physical activity and/or nutrition interventions to promote mobility in older adults: an umbrella review
Source: BMC Geriatr. 2022 Jun 29;22:539. doi: 10.1186/s12877-022-03170-9 (PMC9241281; doi:10.1186/s12877-022-03170-9)
Supplement: Supplementary file 2 — Additional file 2. List of Excluded Studies. [file 12877_2022_3170_MOESM2_ESM.docx]

**Additional file 2: List of Excluded Studies**

**Excluded Studies: Not in English language (n=6)**

- Carcamo-Regla, R., Ulloa, N., Zapata-Lamana, R., Cigarroa, I. Where and how is multicomponent exercise being applied, and in which elderly people, in order to obtain health benefits? A systematic review. *Revista Espanola de Geriatria y Gerontologia.* 2021. 56:100-108
- DeOliveira, C. R., Figueiredo, A. I., Schiavo, A., Martins, L. A., Telles, M. E., Rodrigues, G. A., Mestriner, R. G. Dança como uma intervenção para melhorar a mobilidade e o equilíbrio em idosos: uma revisão de literatura. *Ciência & Saúde Coletiva.* 2020. 25:1913-1924
- Font-Jutgla, C., Mur Gimeno, E., Bort Roig, J., Gomes da Silva, M., Mila Villarroel, R. Effects of mild intensity physical activity on the physical condition of older adults: A systematic review. Rev Esp Geriatr Gerontol. 2020. 55:98-106
- Lohne-Seiler, H., Torstveit, M. K. The importance of physical activity and training for older adults. Norsk Epidemiologi. 2012. 22:165-174
- Rivas, A. R., Rodríguez-Martín, B. Efectividad de las intervenciones multicomponente para la promoción de la actividad física en personas mayores: una revisión sistemática. *Gerokomos.* 2020. 31:149-157
- Roh, K. H., Park, H. A. A meta-analysis of the effect of walking exercise on lower limb muscle endurance, whole body endurance and upper body flexibility in elders. J Korean Acad Nurs. 2013. 43:536-46

**Excluded Studies: Not a systematic review (n=105)**

- Allam, F. Najihah, B, Hamid, Mohd A.H., Buhari, S, Noor, H.Web-based dietary and physical activity intervention programs for Patients with Hypertension: Scoping Review. *J Med Internet Res.* 2021. 23:e22465
- Almeida, F. J., Melo, H. M., Nogueira, R., Prazeres, J., Costa, C., Gambassi, B. B. Do All Resistance Exercise Protocols Improve the Functional Parameters of the Elderly? A Review Study. *Asian J Sports Med.* 2020. 11:1-7
- Arbesman, M., Mosley, L. J. Systematic review of occupation- and activity-based health management and maintenance interventions for community-dwelling older adults. *Am J Occup Ther.* 2012. 66:277-83
- Archer, S. Pilates and Fall Prevention: Weekly Training Reduces Risks for Older Adults. *IDEA Fitness Journal.* 2019. 16:6-6
- Arenas, B. A., Gonzalez, W., Theodore, L. F., Lozado-Tequeanes, L. A., Garcia-Guerra, A., Alvarado, R., Fernandez-Gaxiola, C. A., Rawlinson, J, C., de la Vega, V. A., Neufeld, M. L. Translating Evidence-Based Program Recommendations into Action: The Design, Testing, and Scaling Up of the Behavior Change Strategy EsIAN in Mexico. *J Nutr.* 2019. 149:2310S-2322S
- Astrup, C., O’Connor, M. Fuel for Life: A Literature Review of Nutrition Education and Assessment Among Older Adults Living at Home. *Home Health Care Manag Pract.* 2018. 30:61-69
- Audsley, S., Orton, E., Maula, A., Lam, Z., Kendrick, D., Logan, P. What intervention components work best to maintain physical activity in older people? A systematic review. *Physiotherapy.* 2019. 105:e56-e57
- Balis, L. E., Strayer, T., Ramalingam, N., Wilson, M., Harden, S. M. Open-Access Physical Activity Programs for Older Adults: A Pragmatic and Systematic Review. *Gerontologist.* 2019. 59:e268-e278
- Bennett, J. A. Maintaining and improving physical function in elders. *Annu Rev Nurs Res.* 2002. 20:3-33
- Bergia, R. E., Jun, S, Byers, A., Savaiano, D. Overhauling Nutrition Assistance Programs to Provide Comprehensive Nutrition Support for Older Adults. *Nutr Today.* 2020. 55:30-37
- Bofosa, T., Kam, E., Miangindula, B., Njimbu, F., Nkiama, C. Improvement of the cardiorespiratory endurance, perception of the effort and walking performance of the old people of the hospice Saint Marc of Kingasani by the practice of the adapted physical activities. *European Research Journal.* 2019. 5:36-41
- Borst, S. E. Interventions for sarcopenia and muscle weakness in older people. *Age Ageing.* 2004. 33:548-55
- Breen, L., Stewart, C. E., Onambéle, G. L. Functional benefits of combined resistance training with nutritional interventions in older adults: a review. *Geriatri Gerontol Int.* 2007. 7:326-340
- Brill, P.A., Cornman, C. B., Davis, R. D., Lane, M. J., Sanderson, M., Macera, C. The value of strength training for older adults. *Home Care Provid.* 1999. 4:62-66
- Byrne, C., Faure, C., Keene, D. J., Lamb, S. E. Ageing, Muscle Power and Physical Function: A Systematic Review and Implications for Pragmatic Training Interventions. *Sports Med.* 2016. 46:1311-32
- Campbell, W. W., Leidy, H. J. Dietary protein and resistance training effects on muscle and body composition in older persons. *J Am Coll Nutr.* 2007. 26:696S-703S
- Carella, A. M., Marinelli, T., Pumpo, M. D., Modola, G., Benvenuto, A. Coenzyme Q10 deficiency in elderly: Can nutritional supplementation play a role? Mini review. *European Research Journal.* 2019. 5:166-173
- Castro-Coronado, J, Yasima-Vasquez, G, Zapata-Lamana, R, Toloza-Ramirez, D, Cigarroa, I. Characteristics of resistance training-based programs in older adults with sarcopenia: Scoping review. *Rev Esp Geriatr Gerontol.* 2021.56:279-288
- Chaparro, G. N., Stine-Morrow, E. A. L., Hernandez, M. E. Effects of aerobic fitness on cognitive performance as a function of dual-task demands in older adults. *Exp Gerontol.* 2019. 118:99-105
- Chase, C. A., Mann, K., Wasek, S., Arbesman, M. Systematic review of the effect of home modification and fall prevention programs on falls and the performance of community-dwelling older adults. *Am J Occup Ther.* 2012. 66:284-91
- Chodzko-Zajko, W. J., Proctor, D. N., Fiatarone Singh, M. A., Minson, C. T., Nigg, C. R., Salem, J. G., Skinner, S. J. American College of Sports Medicine position stand. Exercise and physical activity for older adults. *Med Sci Sports Exerc.* 2009. 41:1510-30
- Clifford, T. Nutritional and Pharmacological Interventions to Expedite Recovery Following Muscle-Damaging Exercise in Older Adults: A Narrative Review of the Literature. *J Aging Phys Act.* 2019. 27:914-928
- Coelho-Junior, H., Marzetti, E., Calvani, R., Picca, A., Arai, H., Uchida, M. Resistance training improves cognitive function in older adults with different cognitive status: a systematic review and Meta-analysis. *Aging Ment Health.* 2020. 26:213-224
- Cook, S. B., Cleary, C. J. Progression of blood flow restricted resistance training in older adults at risk of mobility limitations. *Frontiers in Physiology.* 2019. 10:1-10.
- Community-Based Physical Activity Promotes Health. *Tufts University Health & Nutrition Letter.* 2019. 36:2
- Cress, E. M., Buchner, M. D., Prohaska, T., Rimmer, J., Brown, M., Best practices for physical activity programs and behavior counseling in older adult populations. *J Aging Phys Act.* 2005. 13:61-74
- Cyarto, E. V., Moorhead, G. E., Brown, W. J. Updating the evidence relating to physical activity intervention studies in older people. *J Sci Med Sport.* 2004. 7:30-8
- Daly, R. M. Exercise and nutritional approaches to prevent frail bones, falls and fractures: an update. *Climacteric.* 2017. 20:119-124
- Daly, R. M. Independent and combined effects of exercise and vitamin D on muscle morphology, function and falls in the elderly. *Nutrients.* 2010. 2:1005-17
- Davis, J. C., Donaldson, M. G., Ashe, M. C., Khan, K. M. The role of balance and agility training in fall reduction. A comprehensive review. *Eura Medicophys.* 2004. 40:211-21
- Denison, H. J., Cooper, C., Sayer, A. A., Robinson, S. M. Prevention and optimal management of sarcopenia: a review of combined exercise and nutrition interventions to improve muscle outcomes in older people. *Clin Interv Aging.* 2015. 10:859-69
- Dighe, N., Anandh, S., Varadharajulu, G. Effect of weight bearing and neurobic exercises on bone health and physical function in elderly individuals. *Indian Journal of Public Health Research and Development.* 2020. 11:261-266
- Ebeling, P. R., Cicuttini, F., Scott, D., Jones, G. Promoting mobility and healthy aging in men: a narrative review. *Osteoporos Int.* 2019. 30:1911-1922
- Ellingson, T., Conn, V. S. Exercise and quality of life in elderly individuals. *J Gerontol Nurs.* 2000. 26:17-25
- Etnier, J. L., Drollette, E. S., Slutsky, A. B. Physical activity and cognition: A narrative review of the evidence for older adults. *Psych Sport Exerc.* 2019. 42:156-166
- Filho, S., Meneses, E., Albuquerque, J. A. Influência do treinamento com o método pilates sobre o equilíbrio de idosos: uma revisão sistemática. *Man Ther, Posturology Rehabil J.* 2016. 14:1-6
- Fragala, M. S., Cadore, E. L., Dorgo, S., Izquierdo, M., Kraemer, W. J., Peterson, M. D., Ryan, D. E. Resistance Training for Older Adults: Position Statement from the National Strength and Conditioning Association. *J Strength Cond Res.* 2019. 33:2019-2052
- Gajewski, P. D., Falkenstein, M. Physical activity and neurocognitive functioning in aging - a condensed updated review. *Eur Rev Aging Phys Act.* 2016. 13:1-7.
- Gallant, M. P., Tartaglia, M., Hardman, S., Burke, K. Using Tai Chi to Reduce Fall Risk Factors Among Older Adults: An Evaluation of a Community-Based Implementation. *J Appl Gerontol.* 2019. 38:983-998
- Gardner, B., Jovicic, A., Belk, C., Kharicha, K., IIiffe, S., Manthorpe, J., Goodman, C., Drennan, M. V., Walters, K. Specifying the content of home-based health behaviour change interventions for older people with frailty or at risk of frailty: An exploratory systematic review. *BMJ Open.* 2017. 7:e014127.
- Gasquoine, P. G., Chen, P. Y. Effect of physical exercise on popular measures of executive function in older, nonclinical, participants of randomized controlled trials: A meta-analytic review. *Appl Neuropsychol Adult.* 2020. 2020:1-9
- Gawler, S., Skelton, A. D., Dinan-Young, S., Masud, T., Morris, W. R., Griffin, M., Kendrick, D., IIiffe, S. Reducing falls among older people in general practice: The ProAct65+ exercise intervention trial. *Arch Gerontol Geriatr.* 2016. 67:46-54
- Gomersall, J., Tufanaru, C., White, S. The cost effectiveness of exercise for preventing falls in older people living in the community: A systematic review. *JBI Libr Syst Rev.* 2012. 10:3949-3959
- Gomez-Cabello, A., Ara, I., Gonzalez-Aguero A., Casajus J. A., Vicente-Rodriguez, G. Effects of training on bone mass in older adults: a systematic review. *Sports Med.* 2012. 42:301-25
- Gothe, N. P., Khan, I., Hayes, J., Erlenbach, E., Damoiseaux, J. S. Yoga Effects on Brain Health: A Systematic Review of the Current Literature. *Brain Plast.* 2019. 5:105-122
- Gu, M. O., Conn, V. S. Meta-analysis of the effects of exercise interventions on functional status in older adults. *Res Nurs Health.* 2008. 31:594-603
- Gualano, B., Rawson, E. S., Candow, D. G., Chilibeck, P. D. Creatine supplementation in the aging population: effects on skeletal muscle, bone and brain. *Amino Acids.* 2016. 48:1793-805
- Hackney, M. E., Wolf, S. L. Impact of Tai Chi Chu'an practice on balance and mobility in older adults: an integrative review of 20 years of research. *J Geriatr Phys Ther.* 2014. 37:127-35
- Hartman, M. J., Fields, D. A., Byrne, N. M., Hunter, G. R. Resistance training improves metabolic economy during functional tasks in older adults. *J Strength Cond Res.* 2007. 21:91-5
- Hazell, T., Kenno, K., Jakobi, J. Functional benefit of power training for older adults. *J Aging Phys Act.* 2007. 15:349-59
- Hernandez Morante, J. J., Gomez Martinez, C., Morillas-Ruiz, J. M. Dietary Factors Associated with Frailty in Old Adults: A Review of Nutritional Interventions to Prevent Frailty Development. *Nutrients.* 2019. 11:05
- Hita-Contreras, F., Martinez-Amat, A., Cruz-Diaz, D., Perez-Lopez, F. R. Fall prevention in postmenopausal women: the role of Pilates exercise training. *Climacteric.* 2016. 19:229-33
- Hogan, M. Physical and cognitive activity and exercise for older adults: a review. *Int J Aging Hum Dev.* 2005. 60:95-126
- Huber, G. The effect of resistance training on proximal disablement outcomes: a meta-analysis. *Journal of Geriatric Physical Therapy.* 2005. 28:113-113
- Hurley, B. F., Kostek, M. C. Exercise interventions for seniors: What training modality is best for health? *Orthopaedic Physical Therapy Clinics of North America.* 2001. 10:213-25
- Improved nutritional intake reduces risk of malnutrition among elderly-study. *Business Mirror.* 2021
- Jorgic, B., Milanovic, Z., Aleksandrovic, M., Pantelic, S., Daly, D. Effects of deep water running in older adults. a systematic review. *HealthMED.* 2012. 6:3219-3227
- Kaewjoho, C., Mato, L., Thaweewannakij, T., Nakmareong, S., Phadungkit, S., Gaogasigam, C., Amatachaya, S.. Thai dance exercises benefited functional mobility and fall rates among community-dwelling older individuals. *Hong Kong Physiother J.* 2020. 40:19-27
- Kalapotharakos, V. I. Aerobic exercise in older adults: effects on VO2max and functional performance. *Crit Rev Phys Rehabil Med.* 2007. 19:213-225
- Karlsson, M. K., Magnusson, H., von Schewelov, T., Rosengren, B. E. Prevention of falls in the elderly--a review. *Osteoporos Int.* 2013. 24:747-62
- Kasim, N. F., Zanten, J., Veldhuijzen van, Aldred, S. Tai Chi is an effective form of exercise to reduce markers of frailty in older age. *Exp Gerontol.* 2020. 135:110925.
- Keysor, J. J., Brembs, A. Exercise: necessary but not sufficient for improving function and preventing disability? *Curr Opin Rheumatol.* 2011. 23:211-8
- King, A. C., Rejeski, W. J., Buchner, D. M. Physical Activity Interventions Targeting Older Adults: A Critical Review and Recommendations. *Am J Prev Med.* 1998. 15:316-33.
- Klima, W. D., Rabel, M., Mandelblatt, A., Miklosovich, M., Putman, T., Smith, A. Community-Based Fall Prevention and Exercise Programs for Older Adults. *Curr Geriatr Rep.* 2021. 10:58-65
- Law, T. D., Clark, L. A., Clark, B. C. Resistance Exercise to Prevent and Manage Sarcopenia and Dynapenia. *Annu Rev Gerontol Geriatr.* 2016. 36:205-228
- Lelard, T., Ahmaidi, S. Effects of physical training on age-related balance and postural control. *Neurophysiol Clin.* 2015. 45:357-69
- Lemura, L. M., von Duvillard, S. P., Mookerjee, S. The effects of physical training of functional capacity in adults. Ages 46 to 90: a meta-analysis. *J Sports Med Phys Fitness.* 2000. 40:1-10
- Liu, X., Ng, D. H. M., Seah, J. W. T., Munro, Y. L., Wee, S. L. Update on Interventions to Prevent or Reduce Frailty in Community-Dwelling Older Adults: A Scoping Review and Community Translation. *Curr Geriatr Rep.* 2019. 8:72-86
- Maciaszek, J., Osinski, W. The effects of Tai Chi on body balance in elderly people: a review of studies from the early 21st century. *Am J Chin Med.* 2010. 38:219-29
- Mahajan, N., Goyal, P., Pandya, J. The effect of yoga on cardiorespiratory and physical efficiency of the healthy subjects. *Natl J Physiol Pharm Pharmacol.* 2019. 9:543-546
- Maki, E. B., Cheng, C. K., Mansfield, A., Scovil, Y. C., Perry, D. S., Peters, L. A., McKay, S., Lee, T., Marquis, A., Corbeil, P., Fernie, R. G., Liu, B., Mcllroy, E. W. Preventing falls in older adults: new interventions to promote more effective change-in-support balance reactions. *J Electromyogr Kinesiol.* 2008. 18:243-54
- Mallepally, A. R., Karthik, Y., Ansari, N., Chhabra, H. S., Goel, S. A. Reversible Central Hypoventilation Syndrome in Basilar Invagination. *World Neurosurg.* 2019. 131:120-125
- Mangione, K. K., Miller, A. H., Naughton, I. V. Cochrane review: Improving physical function and performance with progressive resistance strength training in older adults. *Phys Ther.* 2010. 90:1711-5
- Marzetti, E., Calvani, R., Tosato, M., Cesari, M., Bari, D. M., Cherubini, A., Broccatelli, M., Savera, G., D’Elia, M., Pahor, M., Bernabei, R., Landi, F.Physical activity and exercise as countermeasures to physical frailty and sarcopenia. *Aging Clin Exp Res.* 2017. 29:35-42
- McBean, L. O’Reilly, S. Diet quality interventions to prevent neurocognitive decline: a systematic review and meta-analysis. *Eur J Clin Nutr.* 2021.
- McGarrigle, L., Todd, C. Promotion of physical activity in older people using mHealth and eHealth technologies: Rapid review of reviews. *J Med Internet Res.* 2020. 22:e22201
- Mendonca, G. V., Pezarat-Correia, P., Vaz, J. R., Silva, L., Almeida, I. D., Heffernan, K. S. Impact of Exercise Training on Physiological Measures of Physical Fitness in the Elderly. *Curr Aging Sci.* 2016. 9:240-259
- Menichetti, J., Cipresso, P., Bussolin, D., Graffigna, G. Engaging older people in healthy and active lifestyles: a systematic review. *Ageing Soc.* 2016. 36:2036-2060
- Netz, Y., Wu, M. J., Becker, B. J., Tenenbaum, G. Physical activity and psychological well-being in advanced age: a meta-analysis of intervention studies. *Psychol Aging.* 2005. 20:272-84
- Nicklett, E. J., Anderson, L. A., Yen, I. H. Gardening Activities and Physical Health Among Older Adults: A Review of the Evidence. *J Appl Gerontol.* 2016. 35:678-90
- Nnodim, J. O., Alexander, N. B. Assessing falls in older adults: a comprehensive fall evaluation to reduce fall risk in older adults. *Geriatrics.* 2005. 60:24-8
- Orellano, E., Colon, W. I., Arbesman, M. Effect of occupation- and activity-based interventions on instrumental activities of daily living performance among community-dwelling older adults: a systematic review. *Am J Occup Ther.* 2012. 66:292-300
- Pahor, M., Guralnik, M. J., Anton, D. S., Ambrosius, T. W., Blair, N. S., Church, S. T., Espeland, A. M.,Fielding, A. R., Gill, M. T., Glynn, W, N., Groessl, J, E., King, C. A., Kritchevsky, B. S., Manini, M. T., McDermott, M. M., Miller, E. M., Newman, B. A., Williamson, D. J. Impact and Lessons from the Lifestyle Interventions and Independence for Elders (LIFE) Clinical Trials of Physical Activity to Prevent Mobility Disability. *J Am Geriatr Soc.* 2020. 68:872-881
- Petrusevski, C., Choo, S., Wilson, M., MacDermid, J., Richardson, J. Interventions to address sedentary behaviour for older adults: a scoping review. *Disabil Rehabil.* 2020. 1-12
- Phillips, S. M., Martinson, W. Nutrient-rich, high-quality, protein-containing dairy foods in combination with exercise in aging persons to mitigate sarcopenia. *Nutr Rev.* 2019. 77:216-229
- Puts, M. T. E., Toubasi, S., Andrew, M. K., Ashe, M. C., Ploeg, J., Atkinson, E., Ayala, P. A., Roy, A., Monforte, R. M., Bergman, H., McGilton, K. Interventions to prevent or reduce the level of frailty in community-dwelling older adults: a scoping review of the literature and international policies. *Age Ageing.* 2017. 46:383-392
- Richardson, D. L., Duncan, M. J., Jimenez, A., Juris, P. M., Clarke, N. D. Affective responses to supervised 10-week programs of resistance exercise in older adults. *J Sport Health Sci.* 2019. 9:604-613.
- Rossato, L. T., Oliveira, Erick P., Schoenfeld, Brad J. Is there sufficient evidence to supplement omega-3 fatty acids to increase muscle mass and strength in young and older adults? *Clin Nutr.* 2020. 39:23-32
- Roswiyani, R., Kwakkenbos, L., Spijker, J., Witteman, C. L. M. The Effectiveness of Combining Visual Art Activities and Physical Exercise for Older Adults on Well-Being or Quality of Life and Mood: A Scoping Review. *J Appl Gerontol.* 2019. 38:1784-1804
- Saffel-Shrier, S., Johnson, M. A., Francis, S. L. Position of the Academy of Nutrition and Dietetics and the Society for Nutrition Education and Behavior: Food and Nutrition Programs for Community-Residing Older Adults. *J Nutr Educ Behav.* 2019. 51:781-797
- Sairam, P., Gulyani, L., Ganesh, S. H. M. Effect of gaze stability exercises on balance confidence and mobility in elderly population. *Indian J Public Health Res Dev.* 2019. 10:477-480
- Senthil, P., Radhakrishnan, R., Kumar, P. G. Mahesh, S. S. Effectiveness of quadriceps resistance training on physical function of elderly people. *J Clin Diagn Res.* 2019. 13:6-8
- Smith, M. The Effects of Tai Chi Versus Non-Tai Chi Interventions on Functional Gait Measures in Older Adults: A Meta-Analysis. *Department of Physical Therapy, California State University, Fresno*. 2018. 1-60.
- Smith, P. J., Potter, G. G., McLaren, M. E., Blumenthal, J. A. Impact of aerobic exercise on neurobehavioral outcomes. *Ment Health Phys Act.* 2013. 6:139-153
- Stanley, C. K., Harrigan, B. P., Serrano, L. E., Kraak, I. V. Applying a multi-dimensional digital food and nutrition literacy model to inform research and policies to enable adults in the U.S. supplemental nutrition assistance program to make healthy purchases in the online food retail ecosystem. *Int J Environ Res Public Health.* 2021. 18:8335
- Taylor, A. H., Cable, N. T., Faulkner, G., Hillsdon, M., Narici, M., Van Der Bij, A. K. Physical activity and older adults: a review of health benefits and the effectiveness of interventions. *J Sports Sci.* 2004. 22:703-25
- Thomas, E., Battaglia, G., Patti, A., Brusa, J., Leonardi, V., Palma, A., Bellafiore, M. Physical activity programs for balance and fall prevention in elderly: A systematic review. *Medicine (Baltimore).* 2019. 98:e16218
- Torbahn, G., Schoene, D., Schwingshackl, L., Rucker, G., Knuttel, H., Kemmler, W., Sieber, C. C., Batsis, A. J., Villareal, T. D., Stroebele-Benschop, N., Volkert, D., Kiesswetter, E. Effective SLOPE: EffectS of Lifestyle interventions in Older PEople with obesity: A systematic review and network meta-analysis protocol. *BMJ Open.* 2020. 10:e038330
- Veronese, N., Maggi, S., Schofield, P., Stubbs, B. Dance movement therapy and falls prevention. *Maturitas.* 2017. 102:1-5
- Wang, R. Y., Wang, Y. L., Cheng, F. Y., Chao, Y. H., Chen, C. L., Yang, Y. R. Effects of combined exercise on gait variability in community-dwelling older adults. *Age (Dordr).* 2015. 37:9780
- Waters, D. L., Ward, A. L., Villareal, D. T. Weight loss in obese adults 65years and older: a review of the controversy. *Exp Gerontol.* 2013. 48:1054-61
- Wu, C. Y., Rodakowski, J. L., Terhorst, L., Karp, J. F., Fields, B., Skidmore, E. R. A. Scoping Review of Nonpharmacological Interventions to Reduce Disability in Older Adults. *Gerontologist.* 2020. 60:e52-e65
- Yao, C. T., Tseng, C. H. Effectiveness of Chair Yoga for Improving the Functional Fitness and Well-being of Female Community-Dwelling Older Adults with Low Physical Activities. *Top Geriatr Rehabil.* 2019. 35:248-254
- Yeun, Y. R. Effectiveness of resistance exercise using elastic bands on flexibility and balance among the elderly people living in the community: a systematic review and meta-analysis. *J Phys Ther Sci.* 2017. 29:1695-1699
- Yoneda, T., Lewis, A. N., Knight, E. J., Rush, J., Vendittelli, R., Kleineidam, L., Hyun, J., Piccinin, M. A., Hofer, M. S., Hoogendijk, O, E., Derby, A. C., Scherer, M., Riedel-Heller, S., Wagner, M., Hout, A., Wang, W., Bennett, A. C., Muniz-Terrera, G. The Importance of Engaging in Physical Activity in Older Adulthood for Transitions Between Cognitive Status Categories and Death: A Coordinated Analysis of 14 Longitudinal Studies. *J Gerontol A Biol Sci Med Sci.* 2021. 76:1661-1667

**Excluded Studies: Specific study population, selected because of a health condition (n=98)**

- Alexander, M. N., Gaydos, K. A., Walch, A. K., McCallum, C. A. Which interventions incorporating physical and cognitive elements are most effective to improve gait in cognitively impaired older adults? A systematic review. *Phys Ther Rev.* 2019. 24:239-247
- Arantes, M. M. P., Alencar, A. M., Dias, C. R., Dias, D. M. J., Pereira, M. S. L. Physical therapy treatment on frailty syndrome: systematic review. *Braz J Phys Ther.* 2009. 13:365-375
- Ashworth, N. L., Chad, K. E., Harrison, E. L., Reeder, B. A., Marshall, S. C. Home versus center based physical activity programs in older adults. *Cochrane Database Syst Rev.* 2005. 1:CD004017
- Aubertin-Leheudre, M., Buckinx, F. Effects of Citrulline alone or combined with exercise on muscle mass, muscle strength, and physical performance among older adults: a systematic review. *Curr Opin Clin Nutr Metab Care.* 2020. 23:8-16
- Bao, W., Sun, Y., Zhang, T., Zou, L., Wu, X., Wang, D., Chen, Z. Exercise programs for muscle mass, muscle strength and physical performance in older adults with sarcopenia: A systematic review and meta-analysis. *Aging Dis.* 2020. 11:863-873
- Barajas-Galindo, E. D., Arnaiz, G. E., Vicente, F. P., Ballesteros-Pomar, D. M. Effects of physical exercise in sarcopenia. A systematic review. *Endocrinologia, Diabetes y Nutricion.* 2021. 68:159-169
- Beauchamp, M. K., Lee, A., Ward, R. F., Harrison, S. M., Bain, P. A., Goldstein, R. S., Brooks, D., Bean, F. J., Jette, M. A. Do Exercise Interventions Improve Participation in Life Roles in Older Adults? A Systematic Review and Meta-Analysis. *Physical Therapy.* 2017. 97:964-974
- Beck, M. A., Husted, M. M., Weekes, E. C., Baldwin, C. Interventions to Support Older People's Involvement in Activities Related to Meals. A Systematic Review. *J Nutr Gerontol Geriatr.* 2020. 39:155-191
- Bird, K. J., Troesch, B., Warnke, I., Calder, C. P. The effect of long chain omega-3 polyunsaturated fatty acids on muscle mass and function in sarcopenia: A scoping systematic review and meta-analysis. *Clin Nutr.* 2021. 46:73-86
- Blazevich, A. J., Wilson, C. J., Alcaraz, P. E., Rubio-Arias, J. A. Effects of Resistance Training Movement Pattern and Velocity on Isometric Muscular Rate of Force Development: A Systematic Review with Meta-analysis and Meta-regression. *Sports Med.* 2020. 50:943-963
- Braga, M. M., Soares, F. C., Costa, A. D. S., Deslandes, A. C., Hardman, C. M. Efeito do exercício físico nas funções cognitivas em idosos depressivos: revisão sistemática. *ConScientiae Saúde.* 2019. 18:141-148
- Buto, S. S. M., de Oliveira, P. M., Carvalho, C., Vassimon-Barroso, V., Takahashi, M. C. A. Effect of complementary therapies on functional capacity and quality of life among prefrail and frail older adults: A systematic review of randomized controlled trials. *Arch Gerontol Geriatr.* 2020. 91:104236.
- Cadore, E. L., Rodriguez-Manas, L., Sinclair, A., Izquierdo, M. Effects of different exercise interventions on risk of falls, gait ability, and balance in physically frail older adults: a systematic review. *Rejuvenation Res.* 2013. 16:105-14
- Campbell, E., Petermann-Rocha, F., Welsh, P., Celis-Morales, C., Pell, P. J., Ho, K. F., Gray, R. S. The effect of exercise on quality of life and activities of daily life in frail older adults: A systematic review of randomised control trials. *Exp Gerontol.* 2021. 147:111287
- Chang, P. S., Knobf, T., Oh, B., Funk, M. Physical and Psychological Health Outcomes of Qigong Exercise in Older Adults: A Systematic Review and Meta-Analysis. *Am J Chin Med.* 2019. 47:301-322
- Chen, Y., Perez-Cueto, A. J. F., Giboreau, A., Mavridis, I., Hartwell, H. The promotion of eating behaviour change through digital interventions. *Int J Environ Res Public Health.* 2020. 17:1-19
- Chou, C. H., Hwang, C. L., Wu, Y. T. Effect of exercise on physical function, daily living activities, and quality of life in the frail older adults: a meta-analysis. *Arch Phys Med Rehabil.* 2012. 93:237-44
- Clegg, A. P., Barber, S. E., Young, J. B., Forster, A., Iliffe, S. J. Do home-based exercise interventions improve outcomes for frail older people? Findings from a systematic review. *Rev Clin Gerontol.* 2012. 22:68-78
- Cui, S., Yang, H., Lin, R., Yang, J., Wahner-Roedler, L. D., Mallory, J. M., Do, A., Bublitz, E. S., Chon, Y. T.., Bauer, A. B., Feng, Z., Tang, C., Zhou, X., Salinas, M., Xu, M. Effects of Tai Chi on Patients with Mild Cognitive Impairment: A Systematic Review and Meta-analysis of Randomized Controlled Trials. *BioMed Res Int.* 2021. 2021:5530149
- Dale, H., Brassington, L., King, K. The impact of healthy lifestyle interventions on mental health and wellbeing: a systematic review. *Ment Health Rev.* 2014. 19:1-26
- Daniels, R., van Rossum, E., de Witte, L., Kempen, G. I., van den Heuvelm, W. Interventions to prevent disability in frail community-dwelling elderly: a systematic review. *BMC Health Serv Res.* 2008. 8:278
- Daryabeygi-Khotbehsara, R., Islam, S. M. S., McVicar, J., Maddison, R., Dunstan, D., Abdelrazek, M. Smartphone-based interventions to reduce sedentary behavior and promote physical activity using integrated dynamic models: Systematic review. *J Med Internet Res.* 2021. 23:e26315
- Davis, A., Sweigart, R., Ellis, R. A systematic review of tailored mHealth interventions for physical activity promotion among adults. *Transl Behav Med.* 2020. 10:1221-1232
- de Labra, C., Guimaraes-Pinheiro, C., Maseda, A., Lorenzo, T., Millan-Calenti, J. C. Effects of physical exercise interventions in frail older adults: a systematic review of randomized controlled trials. *BMC Geriatr.* 2015. 15:154
- de Mello, R. G. B., Dalla Corte, R. R., Gioscia, J., Moriguchi, E. H. Effects of Physical Exercise Programs on Sarcopenia Management, Dynapenia, and Physical Performance in the Elderly: A Systematic Review of Randomized Clinical Trials. *J Aging Res.* 2019. 2019:1959486
- de Oliveira. A. P., Blasczyk, C. J., Junior, S. G., Lagoa, F. K., Soares, M., de Oliveira, J. R., Filho, G. B. J. P., Carregaro, L. R., Martins, R. W. Effects of Elastic Resistance Exercise on Muscle Strength and Functional Performance in Healthy Adults: A Systematic Review and Meta-Analysis. *J Phys Act Health.* 2017. 14:317-32
- Denham-Jones, L., Gaskell, L., Spence, N., Pigott, T. A systematic review of the effectiveness of Pilates on pain, disability, physical function, and quality of life in older adults with chronic musculoskeletal conditions *Musculoskeletal Care*. 2021. 20:10-30.
- Denham-Jones, L., Gaskell, L., Spence, N., Tim, P. A systematic review of the effectiveness of yoga on pain, physical function, and quality of life in older adults with chronic musculoskeletal conditions. *Musculoskeletal Care.* 2021. 20: 47-73
- Dennis, S., Williams, A., Taggart, J., Newall, A., Denney-Wilson, E., Zwar, N., Shortus, T., Harris, F. M. Which providers can bridge the health literacy gap in lifestyle risk factor modification education: a systematic review and narrative synthesis. *BMC Fam Pract.* 2012. 13:44
- Dillon, L., Clemson, L., Ramulu, P., Sherrington, C., Keay, L. A systematic review and meta-analysis of exercise-based falls prevention strategies in adults aged 50+ years with visual impairment. *Ophthalmic Physiol Opt.* 2018. 38:456-467
- Egerton, T., Cordner, T., Schubert, K., Wijesinghe, T., Williams, G. Ballistic Resistance Training: Feasibility, Safety, and Effectiveness for Improving Mobility in Adults With Neurologic Conditions: A Systematic Review. *Arch Phys Med Rehabil.* 2021. 102:735-751
- Emberson, A. M., Lalande, A., Wang, D., McDonough, J. D., Liu, W., Gao, Z. Effectiveness of Smartphone-Based Physical Activity Interventions on Individuals' Health Outcomes: A Systematic Review. *BioMed Res Int.* 2021. 2021:629686
- Fiogbe, E., de Vassimon-Barroso, V., de Medeiros Takahashi, C. A. Exercise training in older adults, what effects on muscle oxygenation? A systematic review. *Arch Gerontol Geriatr.* 2017. 71:89-98
- Frost, H., Haw, S., Frank, J. Interventions in community settings that prevent or delay disablement in later life: an overview of the evidence. *Qual Ageing Older Adults.* 2012. 13:212-230
- Gates, N., Fiatarone Singh, M. A., Sachdev, P. S., Valenzuela, M. The effect of exercise training on cognitive function in older adults with mild cognitive impairment: a meta-analysis of randomized controlled trials. *Am J Geriatr Psychiatry.* 2013. 21:1086-97
- Gine-Garriga, M., Roque-Figuls, M., Coll-Planas, L., Sitja-Rabert, M., Salvam A. Physical exercise interventions for improving performance-based measures of physical function in community-dwelling, frail older adults: a systematic review and meta-analysis. *Arch Phys Med Rehabil.* 2014. 95:753-769.
- Gleeson, M., Sherrington, C., Keay, L. Exercise and physical training improve physical function in older adults with visual impairments but their effect on falls is unclear: a systematic review. *J Physiother.* 2014. 60:130-5
- Hadgraft, N. T., Winkler, E., Climie, R. E., Grace, M. S., Romero, L., Owen, N., Dunstan, D., Healy, G., Dempsey, C. P. Effects of sedentary behaviour interventions on biomarkers of cardiometabolic risk in adults: systematic review with meta-analyses. *Br J Sports Med.* 2020. 55:144-154.
- Hanson, S., Jones, A. Is there evidence that walking groups have health benefits? A systematic review and meta-analysis. *Br J Sports Med.* 2015. 49:710-5
- Harris, K. L., Skou, T. S., Juhl, B. C., Bricca, A. Recruitment and retention rates in randomised controlled trials of exercise therapy in people with multimorbidity: a systematic review and meta-analysis. *Trials.* 2021. 22:396
- Hartley, L., Dyakova, M., Holmes, J., Clarke, A., Lee, M. S., Ernst, E., Rees, K. Yoga for the primary prevention of cardiovascular disease. *Cochrane Database Syst Rev.* 2014. 5:CD010072
- Hartley, L., Flowers, N., Lee, M. S., Ernst, E., Rees, K. Tai chi for primary prevention of cardiovascular disease. *Cochrane Database Syst Rev.* 2014. 4:CD010366
- Hartley, L., Lee, M. S., Kwong, J. S., Flowers, N., Todkill, D., Ernst, E., Rees, K.. Qigong for the primary prevention of cardiovascular disease. *Cochrane Database Syst Rev.* 2015. 6:CD010390
- Holmberg, T., Andersen, S., Rubaek, M., Hitz, M. F., Schonwandt, B. M. T.. Effectiveness of patient education for patients with osteoporosis: a systematic review. *Osteoporos Int.* 2021. 1-19
- Hsiao, B. S., Sibeko, L., Troy, L. M. A Systematic Review of Mobile Produce Markets: Facilitators and Barriers to Use, and Associations with Reported Fruit and Vegetable Intake. *J Acad Nutr Diet.* 2019. 119:76-97 e1
- Husk, K., Lovell, R., Cooper, C., Stahl-Timmins, W., Garside, R. Participation in environmental enhancement and conservation activities for health and well-being in adults: a review of quantitative and qualitative evidence. *Cochrane Database Syst Rev.* 2016. 5:CD010351
- Jadczak, A. D., Makwana, N., Luscombe-Marsh, N., Visvanathan, R., Schultz, T. J. Effectiveness of exercise interventions on physical function in community-dwelling frail older people: an umbrella review of systematic reviews. *JBI Database System Rev Implement Rep.* 2018. 16:752-775
- Jones, L., Bailey, J. S., Rowland, N. S., Alsharif, N., Clifford, T., Shannon, M. O. The Effect of Nitrate-Rich Beetroot Juice on Markers of Exercise-Induced Muscle Damage: A Systematic Review and Meta-Analysis of Human Intervention Trials. *J Diet Suppl.* 2021.1-23
- Kamioka, H., Tsutani, K., Katsumata, Y., Yoshizaki, T., Okuizumi, H., Okada, S., Park, S. J., Kitayuguchi, J., Abe, T., Mutoh, Y. Effectiveness of Pilates exercise: A quality evaluation and summary of systematic reviews based on randomized controlled trials. *Complement Ther Med.* 2016. 25:1-19
- Khamzina, M., Parab, V. K., Bullard, T., An, R., Grigsby-Toussaint, S. D. Impact of Pokemon Go on Physical Activity: A Systematic Review and Meta-Analysis. *Am J Prev Med.* 2020. 58:270-282
- Khor, Y. P., Vearing, Rebecca M., Charlton, E. K. The effectiveness of nutrition interventions in improving frailty and its associated constructs related to malnutrition and functional decline among community-dwelling older adults: A systematic review. *J Hum Nutr Diet.* 2021.1-17
- Kidd, T., Mold, F., Jones, C., Ream, E., Grosvenor, W., Sund-Levander, M., Tingstrom, P., Carey, N. What are the most effective interventions to improve physical performance in pre-frail and frail adults? A systematic review of randomised control trials. *BMC Geriatr.* 2019. 19:184
- Kimber, K., Gibbs, M., Weekes, C. E., Baldwin, C. Supportive interventions for enhancing dietary intake in malnourished or nutritionally at-risk adults: a systematic review of nonrandomised studies. *Journal of Human Nutrition & Dietetics.* 2015. 28:517-545
- Laranjo, L., Ding, D., Heleno, B., Kocaballi, B., Quiroz, C. J., Tong, L. H., Chahwan, B., Neves, L. A., Gabarron, E., Dao, P. K., Rodrigues, D., Neves, C. G., Antunes, L. M., Coiera, E., Bates, W. D. Do smartphone applications and activity trackers increase physical activity in adults? Systematic review, meta-analysis and metaregression. *Br J Sports Med.* 2021. 55:422-432
- Lee, P. H., Lee, Y. S, Chan, D. C. Interventions targeting geriatric frailty: A systemic review. *Journal of Clinical Gerontology and Geriatrics.* 2012. 3:47-52
- Lee, S. H., Kim, H. S. Exercise Interventions for Preventing Falls Among Older People in Care Facilities: A Meta-Analysis. *Worldviews on Evidence-Based Nursing.* 2017. 14:74-80
- Liao, C. D., Lee, P. H., Hsiao, D. J., Huang, S. W., Tsauo, J. Y., Chen, H. C., Liou, T. H. Effects of Protein Supplementation Combined with Exercise Intervention on Frailty Indices, Body Composition, and Physical Function in Frail Older Adults. *Nutrients.* 2018. 10:1916.
- Lin, S. F., Sung, H. C. The effectiveness of resistance training with thera band on physiological functions for older adults: A systematic review. *JBI Lib Syst Rev.* 2012. 10:S239-S248
- Lopez, P., Pinto, R. S., Radaelli, R., Rech, A., Grazioli, R., Izquierdo, M., Cadore, E. L. Benefits of resistance training in physically frail elderly: a systematic review. *Aging Clin Exp Res.* 2018. 30:889-899
- Luo, H., Newton, R. U., Ma'ayah, F., Galvao, D. A., Taaffe, D. R. Recreational soccer as sport medicine for middle-aged and older adults: a systematic review. *BMJ Open Sport Exerc Med.* 2018. 4:e000336
- McGrattan, A., Aller, C., Narytnyk, A., Reidpath, D., Keage, H., Mohan, D., Su. T. T., Stephan, B., Robinson, L., Siervo, M. Nutritional interventions for the prevention of cognitive impairment and dementia in developing economies in East-Asia: a systematic review and meta-analysis. *Crit Rev Food Sci Nutr.* 2020. 62;1-18
- Miller, K. J., Suarez-Iglesias, D., Varela, S., Rodriguez, D., Ayan, C. Exercise for Nonagenarians: A Systematic Review. *J Geriatr Phys Ther.* 2019. 43:208-218.
- Monninghoff, A., Kramer, N. J., Hess, J. A., I., Kamila, T., Gisbert W.T., Car, T. L., Muller-Riemenschneider, F. Long-term Effectiveness of Health Physical Activity Interventions: Systematic Review and Meta-analysis of Randomized Controlled Trials. *J Med Internet Res.* 2021. 23:e26699
- Moore, S. G., Donnelly, J. K., Jones, S., Cade, J. E. Effect of Educational Interventions on Understanding and Use of Nutrition Labels: A Systematic Review. *Nutrients.* 2018. 10:1432.
- Morilla-Herrera, J. C., Martin-Santos, F. J., Caro-Bautista, J., Saucedo-Figueredo, C., Garcia-Mayor, S., Morales-Asencio, J. M. Effectiveness of food-based fortification in older people a systematic review and meta-analysis. *J Nutr Health Aging.* 2016. 20:178-184
- Naseri, C., Haines, T. P., Etherton-Beer, C., McPhail, S., Morris, M. E., Flicker, L., Netto, J., Francis-Coad, J., Lee, A. D., Shorr, R., Hill, A.Reducing falls in older adults recently discharged from hospital: a systematic review and meta-analysis. *Age Ageing.* 2018. 47:512-519.
- Nash, K. C. M. The effects of exercise on strength and physical performance in frail older people: A systematic review. *Rev Clin Gerontol.* 2012. 22:274-285
- Orange, T. S., Madden, A. L., Vince, V. R. Resistance training leads to large improvements in strength and moderate improvements in physical function in adults who are overweight or obese: a systematic review. *J Physiother.* 2020. 66:214-224
- Ou, S. L. J., Yang, D., Liu, H. M. Effects of anthocyanins in composite meals on cardiometabolic outcomes-a systematic review of randomized controlled feeding trials. *Nutrients.* 2020. 12:1-23
- Pan, X., Tian, L., Yang, F., Sun, J., Li, X., Su, X., Liu, X., Liu, C., Xing, Y., An, N., Gao, Y., Xing, Y. Tai Chi as a Therapy of Traditional Chinese Medicine on Reducing Blood Pressure: A Systematic Review of Randomized Controlled Trials. *Evid Based Complement Alternat Med.* 2021. 2021:4094325
- Paramastri, R., Pratama, A. S., Ho, N. K. D., Purnamasari, D. S., Mohammed, Z. A., Galvin, J. C., Hsu, E. Y., Tanweer, A., Humayun, A., Househ, M., Iqbal, U. Use of mobile applications to improve nutrition behaviour: A systematic review. *Comput Methods Programs Biomed.* 2020. 192:105459
- Patnode, C. D., Perdue, L. A., Rossom, R. C., Rushkin, M. C., Redmond, N., Thomas, R. G. Screening for Cognitive Impairment in Older Adults: An Evidence Update for the U.S. Preventive Services Task Force. *Agency for Healthcare Research and Quality.* 2020. 189:1-473.
- Paw, A., Chin, M. J., van Uffelen, J. G., Riphagen, I., van Mechelen, W. The functional effects of physical exercise training in frail older people: a systematic review. *Sports Med.* 2008. 38:781-93
- Paw, M. J. M., van Uffelen, J. G. Z., Riphagen, I. I., van Mechelen, W. A systematic review of exercise programs for the frail elderly. *Long-Term Care Interface.* 2007. 8:14-19
- Pedro, L., Mikel, I., Regis, R., Graciele, S., Rafael, G., Silveira, P. R., Cadore, L. E. Effectiveness of Multimodal Training on Functional Capacity in Frail Older People: A Meta-Analysis of Randomized Controlled Trials. *J Aging Phys Act.* 2018. 26:407-418
- Richards, J., Hillsdon, M., Thorogood, M., Foster, C. Face-to-face interventions for promoting physical activity. *Cochrane Database Syst Rev.* 2013. 9:CD010392
- Roig, M., O'Brien, K., Kirk, G., Murray, R., McKinnon, P., Shadgan, B., Ried, D. W. The effects of eccentric versus concentric resistance training on muscle strength and mass in healthy adults: a systematic review with meta-analysis. *Br J Sports Med.* 2009. 43:556-68
- Russo, J. M., Kanevsky, A., Leis, A., Iturry, M., Roncoroni, M., Serrano, C., Cristalli, D., Ure, J., Zuin, D. Role of physical activity in preventing cognitive impairment and dementia in older adults: A systematic review. *Neurologia Argentina.* 2020. 12:124-137
- Saragih, D. I., Yang, Y., Saragih, S. I., Batubara, O. S., Lin, C. Effects of resistance bands exercise for frail older adults: A systematic review and meta-analysis of randomised controlled studies. *J Clin Nurs.* 2021. 31:43-61
- Schechtman, K. B., Ory, M. G. The effects of exercise on the quality of life of frail older adults: A preplanned meta-analysis of the FICSIT trials. *Ann Behav Med.* 2001. 23:186-197
- Sequi-Dominguez, I., Alvarez-Bueno, C., Martinez-Vizcaino, V., Fernandez-Rodriguez, R., Lara, S. A., Cavero-Redondo, I. Effectiveness of mobile health interventions promoting physical activity and lifestyle interventions to reduce cardiovascular risk among individuals with metabolic syndrome: Systematic review and meta-analysis. *J Med Internet Res.* 2020. 22:e17790
- Seron, P., Lanas, F., Pardo Hernandez, H., Bonfill Cosp, X. Exercise for people with high cardiovascular risk. *Cochrane Database Syst Rev.* 2014. 8:CD009387
- Sexton, B. P., Taylor, N. F. To sit or not to sit? A systematic review and meta-analysis of seated exercise for older adults. *Australas J Ageing.* 2019. 38:15-27
- Shannon. M. O., Mendes, I., Kochl, C., Mazidi, M., Ashor, W. A., Rubele, S., Minihane, A., Mathers, C. J., Siervo, M. Mediterranean diet increases endothelial function in adults: A systematic review and meta-Analysis of randomized controlled trials. *J Nutr.* 2020. 150:1151-1159
- Silva, R. B., Aldoradin-Cabeza, H., Eslick, G. D., Phu, S., Duque, G. The Effect of Physical Exercise on Frail Older Persons: A Systematic Review. *J Frailty Aging.* 2017. 6:91-96
- Sweeting, J., Ding, D., Merom, D., Astuti, P, Antoun, M., Edwards, K. Physical activity interventions for adults who are visually impaired: A systematic review and meta-analysis. *BMJ Open.* 2020. 10:e034036
- Talar, K., Kalamacka, E., Hernandez-belmonte, A., Courel-ibanez, J., Vetrovsky, T., Steffl, M. Benefits of resistance training in early and late stages of frailty and sarcopenia: A systematic review and meta-analysis of randomized controlled studies. *J Clin Med.* 2021. 10:1630
- Theodorakopoulos, C., Jones, J., Bannerman, E., Greig, C. A. Effectiveness of nutritional and exercise interventions to improve body composition and muscle strength or function in sarcopenic obese older adults: A systematic review. *Nutr Res.* 2017. 43:3-15
- Theou, O., Stathokostas, L., Roland, K. P., Jakobi, J. M., Patterson, C., Vandervoort, A. A., Jones, R. G. The effectiveness of exercise interventions for the management of frailty: a systematic review. *J Aging Res.* 2011. 2011:569194
- Torres, R., Ribeiro, F., Alberto Duarte, J., Cabri, J. M. Evidence of the physiotherapeutic interventions used currently after exercise-induced muscle damage: systematic review and meta-analysis. *Phys Ther Sport.* 2012. 13:101-14
- Trabal, J., Farran-Codina, A. Effects of dietary enrichment with conventional foods on energy and protein intake in older adults: a systematic review. *Nutr Rev.* 2015. 73:624-33
- Youkhana, S., Dean, C. M., Wolff, M., Sherrington, C., Tiedemann, A. Yoga-based exercise improves balance and mobility in people aged 60 and over: a systematic review and meta-analysis. *Age Ageing.* 2016. 45:21-9
- Wang, Le-Cong, Ye, Ming-Zhu, Xiong, Jian, Wang, Xiao-Qian, Wu, Jia-Wei, Zheng, Guo-Hua. Optimal exercise parameters of tai chi for balance performance in older adults: A meta-analysis. *J Am Geriatr Soc.* 2021. 69:2000-2010
- Yin, Y., Liu, W., Fan, T, Leung, K., Ng, M., Tsang, Y., Wong, K., Valimaki, M. Effectiveness of Nutritional Advice for Community-Dwelling Obese Older Adults With Frailty: A Systematic Review and Meta-Analysis. *Front Nutr.* 2021. 8:619903
- Zhang, Y., Li, C., Zou, L., Liu, X., Song, W. The Effects of Mind-Body Exercise on Cognitive Performance in Elderly: A Systematic Review and Meta-Analysis. *Int J Environ Res Public Health.* 2018. 15:09
- Zhang, Y., Zou, L., Chen, S., Bae, H. J., Kim, Y. D., Liu, S. X. Effects and Moderators of Exercise on Sarcopenic Components in Sarcopenic Elderly: A Systematic Review and Meta-Analysis. *Front Med.* 2021. 8:649748
- Zhu, H., An, R. Impact of home-delivered meal programs on diet and nutrition among older adults: a review. *Nutr Health.* 2013. 22:89-103
- Zhu, L., Wu, W., Chen, M., Xu, D., Xu, H., Liu, L., Liu, J., Zhu, Z. Effects of Nonpharmacological Interventions on Balance Function in Patients with Osteoporosis or Osteopenia: A Network Meta-Analysis of Randomized Controlled Trials. *Evid Based Complement Alternat Med.* 2021. 2021:6662510

**Excluded Studies: Intervention not community and group based (n=61)**

- Aboutorabi, A., Arazpour, M., Bahramizadeh, M., Farahmand, F., Fadayevatan R. Effect of vibration on postural control and gait of elderly subjects: a systematic review. *Aging Clin Exp Res.* 2018. 30:713-726
- Ahn, S., Anderson, G. J., Chung, W. J., Crane, K. M., Bassett, R. D. The Effects of Multi-Domain Interventions on Cognition: A Systematic Review. *West J Nurs Res.* 2021. 1-21
- Alhasan, H., Hood, V., Mainwaring, F. The effect of visual biofeedback on balance in elderly population: a systematic review. *Clin Interv Aging.* 2017. 12:487-497
- Ammar, A., Trabelsi, K., Muller, P., Bouaziz, B., Boukhris, O., Glenn, J. M., Bott, N., Driss, T., Chtourou, H., Muller, N., Hokelmann, A. The Effect of (Poly)phenol-Rich Interventions on Cognitive Functions and Neuroprotective Measures in Healthy Aging Adults: A Systematic Review and Meta-Analysis. *J Clin Med.* 2020. 9:19
- Beaumont, A. J., Grace, F. M., Richards, J. C., Campbell, A. K., Sculthorpe, N. F. Aerobic Training Protects Cardiac Function During Advancing Age: A Meta-Analysis of Four Decades of Controlled Studies. *Sports Med.* 2019. 49:199-219
- Beswick, A. D., Rees, K., Dieppe, P., Ayis, S., Gooberman-Hill, R., Horwood, J., Ebrahim, S. Complex interventions to improve physical function and maintain independent living in elderly people: a systematic review and meta-analysis. *Lancet.* 2008. 371:725-735
- Boulton, R. E., Horne, M., Todd, C. Involving Older Adults in Developing Physical Activity Interventions to Promote Engagement: a Literature Review. *J Popul Ageing.* 2020. 13:325-345
- Burk, C., Perry, J., Lis, S., Dischiavi, S., Bleakley, C. Can Myofascial Interventions Have a Remote Effect on ROM? A Systematic Review and Meta-Analysis. *J Sport Rehabil.* 2019. 29:1-23.
- Coelho-Junior, H. J., Milano-Teixeira, L., Rodrigues, B., Bacurau, R., Marzetti, E., Uchida, M. Relative Protein Intake and Physical Function in Older Adults: A Systematic Review and Meta-Analysis of Observational Studies. *Nutrients.* 2018. 10:19
- Corregidor-Sanchez, A., Segura-Fragoso, A., Criado-Alvarez, J., Rodriguez-Hernandez, M., Mohedano-Moriano, A., Polonio-Lopez, B. Effectiveness of Virtual Reality Systems to Improve the Activities of Daily Life in Older People. *Int J Environment Res Public Health.* 2020. 17:1-21
- Costello, E., Edelstein, J. E. Update on falls prevention for community-dwelling older adults: review of single and multifactorial intervention programs. *J Rehabil Res Dev.* 2008. 45:1135-52
- Cunningham, C., O' Sullivan, R., Caserotti, P., Tully, M. A. Consequences of physical inactivity in older adults: A systematic review of reviews and meta-analyses. *Scand J Med Sci Sports.* 2020. 30:816-827
- Dabbous, Massar, Hastings, Rebecca, Baldwin, Christine, Weekes, C. Elizabeth. The role of non-dietetic healthcare professionals in managing interventions among adults at risk of malnutrition: A systematic review. *Clin Nutr.* 2021. 40:4509-4525
- Delafontaine, A., Vialleron, T., Fourcade, P., Yiou, E., Ditcharles, S. Effects of stretching exercises on human gait: A systematic review and meta-analysis. *F1000Res.* 2020. 9:984
- Donath, L., Rossler, R., Faude, O. Effects of Virtual Reality Training (Exergaming) Compared to Alternative Exercise Training and Passive Control on Standing Balance and Functional Mobility in Healthy Community-Dwelling Seniors: A Meta-Analytical Review. *Sports Med.* 2016. 46:1293-309
- Elavsky, S., Knapova, L., Klocek, A., Smahel, D. Mobile Health Interventions for Physical Activity, Sedentary Behavior, and Sleep in Adults Aged 50 Years and Older: A Systematic Literature Review. *J Aging Phys Act.* 2019. 27:565-593
- Engeroff, T., Ingmann, T., Banzer, W. Physical Activity Throughout the Adult Life Span and Domain-Specific Cognitive Function in Old Age: A Systematic Review of Cross-Sectional and Longitudinal Data. *Sports Med.* 2018. 48:1405-1436
- Fang, Qun, Ghanouni, Parisa, Anderson, Sarah E., Touchett, Hilary, Shirley, Rebekah, Fang, Fang, Fang, Chao. Effects of Exergaming on Balance of Healthy Older Adults: A Systematic Review and Meta-analysis of Randomized Controlled Trials. *Games Health J.* 2020. 9:11-23
- Granic, A., Hurst, C., Robinson, M. S., Sayer, A. A., Dismore, L. Myoprotective whole foods, muscle health and sarcopenia: A systematic review of observational and intervention studies in older adults. *Nutrients.* 2020. 12:1-32
- Hill, K. D., Hunter, S. W., Batchelor, F. A., Cavalheri, V., Burton, E. Individualized home-based exercise programs for older people to reduce falls and improve physical performance: A systematic review and meta-analysis. *Maturitas.* 2015. 82:72-84
- Hilliere, C., Collado-Mateo, D., Villafaina, S., Duque-Fonseca, P., Parraca, J. A. Benefits of Hippotherapy and Horse Riding Simulation Exercise on Healthy Older Adults: A Systematic Review. *PM R.* 2018. 10:1062-1072
- Hopewell, S., Adedire, O., Copsey, B. J., Boniface, G. J., Sherrington, C., Clemson, L., Close, C. J., Lamb, E. S. Multifactorial and multiple component interventions for preventing falls in older people living in the community. *Cochrane Database Syst Rev.* 2018. 7:CD012221
- Hughes, K. J., Salmon, N., Galvin, R., Casey, B., Clifford, A. M. Interventions to improve adherence to exercise therapy for falls prevention in community-dwelling older adults: systematic review and meta-analysis. *Age Ageing.* 2019. 48:185-195
- Ilgaz, A., Gozum, S. Health promotion interventions for older people living alone: a systematic review. *Perspect Public Health.* 2019. 139:255-263
- Kulkarni, D., Evans, M., Gregory, S. Effectiveness of eccentric-biased exercise interventions in reducing the incidence of falls and improving functional performance in older adults: a systematic review. *Eur Geriatr Med.* 2021. 1-14
- Lam, F. M., Lau, R. W., Chung, R. C., Pang, M. Y. The effect of whole body vibration on balance, mobility and falls in older adults: a systematic review and meta-analysis. *Maturitas.* 2012. 72:206-13
- Lau, R. W., Liao, L. R., Yu, F., Teo, T., Chung, R. C., Pang, M. Y. The effects of whole body vibration therapy on bone mineral density and leg muscle strength in older adults: a systematic review and meta-analysis. *Clin Rehabil.* 2011. 25:975-88
- Laufer, Y., Dar, G., Kodesh, E. Does a Wii-based exercise program enhance balance control of independently functioning older adults? A systematic review. *Clin Interv Aging.* 2014. 9:1803-13
- Leonhardt, R., Becker, C., Gros, M., Mikolaizak, A. S. Impact of the backward chaining method on physical and psychological outcome measures in older adults at risk of falling: a systematic review. *Aging Clin Exp Res.* 2020. 32:985-997
- Lim, Y. M., Kim, H., Cha, Y. J. Effects of environmental modification on activities of daily living, social participation and quality of life in the older adults: a meta-analysis of randomized controlled trials. *Disabil Rehabil Assist Technol.* 2020. 15:132-140
- Limongi, F., Siviero, P., Noale, M., Maggi, S., Bozanic, A., Veronese, N. The Effect of Adherence to the Mediterranean Diet on Late-Life Cognitive Disorders: A Systematic Review. *J Am Med Dir Assoc.* 2020. 21:1402-1409
- Lindberg, J., Carlsson, J. The effects of whole-body vibration training on gait and walking ability - a systematic review comparing two quality indexes. *Physiother Theory Pract.* 2012. 28:485-98
- Liu, C. J., Chang, W. P., Chang, M. C. Occupational Therapy Interventions to Improve Activities of Daily Living for Community-Dwelling Older Adults: A Systematic Review. *Am J Occup Ther.* 2018. 72:4.
- Lorenzo-López, L., Maseda, A., de Labra, C., Regueiro-Folgueira, L., Rodríguez-Villamil J. L., Millán-Calent J. C. Nutritional determinants of frailty in older adults: A systematic review. *BMC Geriatr.* 2017. 17:1-13
- McNarry, A. M., Mackintosh, A. K., Hudson, J., Knight, L. R., Chalabaev, A. Do age stereotype-based interventions affect health-related outcomes in older adults? A systematic review and future directions. *Br J Health Psychol.* 2021. 1-36
- Mikhael, M., Orr, R., Fiatarone Singh, M. A. The effect of whole body vibration exposure on muscle or bone morphology and function in older adults: a systematic review of the literature. *Maturitas.* 2010. 66:150-7
- Milte, C. M., McNaughton, S. A. Dietary patterns and successful ageing: a systematic review. *Eur J Nutr.* 2016. 55:423-450
- Mol, A., Reijnierse, E. M., Bui Hoang, P. T. S., van Wezel, R. J. A., Meskers, C. G. M., Maier, A. B. Orthostatic hypotension and physical functioning in older adults: A systematic review and meta-analysis. *Ageing Res Rev.* 2018. 48:122-144
- Moore, A., Motagh, S., Sadeghirad, B., Begum, H., Riva, J. J., Gaber, J., Dolovich, L. Volunteer Impact on Health-Related Outcomes for Seniors: a Systematic Review And Meta-Analysis. *Can Geriatr J.* 2021. 24:44-72
- Motamed-Jahromi, M., Kaveh, H. M. Effective Interventions on Improving Elderly's Independence in Activity of Daily Living: A Systematic Review and Logic Model. *Front Public Health.* 2020. 8:516151
- Molina, K. I., Ricci, N. A., de Moraes, S. A., Perracini, M. R. Virtual reality using games for improving physical functioning in older adults: a systematic review. *J Neuroeng Rehabil.* 2014. 11:156
- Nowson, C. A., Service, C., Appleton, J., Grieger, J. A. The Impact of Dietary Factors on Indices of Chronic Disease in Older People: A Systematic Review. *J Nutr Health Aging.* 2018. 22:282-296
- Orr, R. The effect of whole body vibration exposure on balance and functional mobility in older adults: a systematic review and meta-analysis. *Maturitas.* 2015. 80:342-358
- Patience, J., Lai, K. S. P., Russell, E., Vasudev, A., Montero-Odasso, M., Burhan, A. M. Relationship Between Mood, Thinking, and Walking: A Systematic Review Examining Depressive Symptoms, Executive Function, and Gait. *Am J Geriatr Psychiatry.* 2019. 27:1375-1383
- Pedersen, A. N., Cederholm, T. Health effects of protein intake in healthy elderly populations: a systematic literature review. *Food Nutr Res.* 2014. 58:1-39.
- Pedersen, M., Harris, J. K., Brown, B., Anderson, K., Lewis, P. J. A Systematic Review of Interventions to Increase Physical Activity Among American Indian and Alaska Native Older Adults. *Gerontologists.* 2021. 1-12
- Pollock R. D., Martin F. C., Newham D, J. The effect of whole body vibration on older people: a systematic review. *Phys Ther Rev.* 2012. 17:110-123
- Rogan, S., de Bruin, E. D., Radlinger, L., Joehr, C., Wyss, C., Stuck, N. J., Bruelhart, J., Bie, A. R., Hilfiker, R. Effects of whole-body vibration on proxies of muscle strength in old adults: a systematic review and meta-analysis on the role of physical capacity level. *Eur Rev Aging Phys Act.* 2015. 12:12
- Rogan, S.. Taeymans, J., Radlinger, L., Naepflin, S., Ruppen, S., Bruelhart, Y., Hilfiker, R. Effects of whole-body vibration on postural control in elderly: An update of a systematic review and meta-analysis. *Arch Gerontol Geriatr.* 2017. 73:95-112
- Sherrington, C., Oliveira, S. J., Zheng, Y. R. E., Franco, R. M., Tiedemann, A. Effect of interventions using physical activity trackers on physical activity in people aged 60 years and over: a systematic review and meta-analysis. *Br J Sports Med.* 2020. 54:1188-1194
- Sakaki, K., Nouchi, R., Matsuzaki, Y., Saito, T., Dinet, J., Kawashima, R. Benefits of VR Physical Exercise on Cognition in Older Adults with and without Mild Cognitive Decline: A Systematic Review of Randomized Controlled Trials. *Healthcare.* 2021. 9:883
- Schoene, D., Valenzuela, T., Lord, S. R., de Bruin, E. D. The effect of interactive cognitive-motor training in reducing fall risk in older people: a systematic review. *BMC Geriatr.* 2014. 14:107
- Schwenk, M., Jordan, E. D., Honarvararaghi, B., Mohler, J., Armstrong, D. G., Najafi, B. Effectiveness of foot and ankle exercise programs on reducing the risk of falling in older adults: a systematic review and meta-analysis of randomized controlled trials. *J Am Podiatr Med Assoc.* 2013. 103:534-47
- Silva, R., Pizato, N., da Mata, F., Figueiredo, A., Ito, M., Pereira, M. G. Mediterranean Diet and Musculoskeletal-Functional Outcomes in Community-Dwelling Older People: A Systematic Review and Meta-Analysis. *J Nutr Health Aging.* 2018. 22:655-663
- Spielau Riise, U., Gudbrandsen, O. A., Ranhoff, A. H. Vitamin D supplementation and its influence on muscle strength and mobility in community-dwelling older persons: a systematic review and meta-analysis. *J Hum Nutr Diet.* 2017. 30:3-15
- Wang, D., MacMillan, T. The benefits of gardening for older adults: a systematic review of the literature. *Act Adapt Aging.* 2013. 37:153-181
- Welch, V., Mathew, M. C., Babelmorad, P., Li, Y., Ghogomu, T. E., Borg, C. J., Monserrat, K., Elizabeth, L., Anne, M., Sue, N., Jason W., Pottie, K., Rogers, M., Sadana, R., Saran, A., Shea, B., Sheehy, L., Sveistrup, H., Tanuseputro, P., Joanna, T., Coon,W., Peter,Z., Wei,H. Health, social care and technological interventions to improve functional ability of older adults living at home: An evidence and gap map. *Campbell Syst Rev.* 2021. 17:1-245
- Western, J. M, Armstrong, E. G. M., Islam, I., Morgan, K., Jones, F. U., Kelson, J. M. The effectiveness of digital interventions for increasing physical activity in individuals of low socioeconomic status: a systematic review and meta-analysis. *Int J Behav Nutr Phys Act.* 2021. 18:148
- Wu, H., Xia, Y., Jiang, J., Du, H., Guo, X., Liu, X., Li, C., Huang, G., Niu, K. Effect of beta-hydroxy-beta-methylbutyrate supplementation on muscle loss in older adults: A systematic review and meta-analysis. *Arch Gerontol Geriatr.* 2015. 61:168-175
- Xu, Z. R., Tan, Z. J., Zhang, Q., Gui, Q. F., Yang, Y. M. Clinical effectiveness of protein and amino acid supplementation on building muscle mass in elderly people: a meta-analysis. *PLoS One.* 2014. 9:e109141
- Zhong, S., Lee, C., Bian, J., Foster, J. M. Intergenerational communities: A systematic literature review of intergenerational interactions and older adults' health-related outcomes. *Soc Sci Med.* 2020. 264:113374

**Excluded Studies: No mobility related outcomes reported or unrelated outcomes (e.g., falls prevention, body composition only) without other mobility measures (n=69)**

- Beijersbergen, C. M., Granacher, U., Vandervoort, A. A., DeVita, P., Hortobagyi, T. The biomechanical mechanism of how strength and power training improves walking speed in old adults remains unknown. *Ageing Res Rev.* 2013. 12:618-27
- Burton, E., Farrier, K., Lewin, G., Petrich, M., Boyle, E., Hill, K. D. Are interventions effective in improving the ability of older adults to rise from the floor independently? A mixed method systematic review. *Disabil Rehabil.* 2020. 42:743-753
- Campbell, A., Grace, F., Ritchie, L., Beaumont, A., Sculthorpe, N. Long-Term Aerobic Exercise Improves Vascular Function Into Old Age: A Systematic Review, Meta-Analysis and Meta Regression of Observational and Interventional Studies. *Front Physiol.* 2019. 10:31
- Campos, M. D., Ferreira, L. D., Goncalves, H. G., DeOliveira, C. J., Farche, S. C. A, Juliana H. Effects of aquatic physical exercise on neuropsychological factors in older people: A systematic review. *Arch Gerontol Geriatr.* 2021. 96:104435
- Carvalho, A., Rea, I. M., Parimon, T., Cusack, B. J. Physical activity and cognitive function in individuals over 60 years of age: a systematic review. *Clin Interv Aging.* 2014. 9:661-82
- Chan, J. S. Y., Deng, K., Wu, J., Yan, J. H. Effects of Meditation and Mind-Body Exercises on Older Adults' Cognitive Performance: A Meta-analysis. *Gerontologist.* 2019. 59:782-90
- Chen, F. T., Hopman, R. J., Huang, C. J., Chu, C. H., Hillman, C. H., Hung, T. M., Chang, Y. The Effect of Exercise Training on Brain Structure and Function in Older Adults: A Systematic Review Based on Evidence from Randomized Control Trials. *J Clin Med.* 2020. 9:27
- Chen, F., Etnier, Jennifer L., Chan, K., Chiu, P., Hung, T., Chang, Y. Effects of Exercise Training Interventions on Executive Function in Older Adults: A Systematic Review and Meta‑Analysis. *Sports Med.* 2020. 50:1451-1467
- Chiu, Huei-Ling, Lo, Yun-Ting, Lee, Shu-Chun, Yeh, Ting-Ting, Liang, Pei-Jung. The effects of the Otago Exercise Programme on actual and perceived balance in older adults: A meta-analysis. *PLoS ONE.* 2021. 16:e0255780
- Cohelo-Junior, H., Marzetti, E., Calvani, R., Picca, A., Uchida, M., Arai, H. Resistance training improves cognitive function in older adults with different cognitive status: a systematic review and meta-analysis. *Aging Mental Health*. 2020. 26: 1-12.
- Devereux-Fitzgerald, A., Powell, R., Dewhurst, A., French, D. P. The acceptability of physical activity interventions to older adults: A systematic review and meta-synthesis. *Soc Sci Med.* 2016. 158:14-23
- Dipietro, L., Campbell, W. W., Buchner, D. M., Erickson, K. I., Powell, K. E., Bloodgood, B., Hughes, T., Day, R. K., Piercy, L, K., Vaux-Bjerke, A., Olson, D, R. Physical Activity, Injurious Falls, and Physical Function in Aging: An Umbrella Review. *Med Sci Sports Exerc.* 2019. 51:1303-1313
- Erickson, K. I., Hillman, C., Stillman, C. M., Ballard, R. M., Bloodgood, B., Conroy, D. E., Macko, R., Marquez, X. D., Petruzzello, J. S., Powell, E. K. Physical Activity, Cognition, and Brain Outcomes: A Review of the 2018 Physical Activity Guidelines. *Med Sci Sports Exerc.* 2019. 51:1242-1251
- Feter, N., Alt, R., Dias, M. G., Rombaldi, A. J. How do different physical exercise parameters modulate brain-derived neurotrophic factor in healthy and non-healthy adults? A systematic review, meta-analysis and meta-regression. *Sci Sports.* 2019. 34:293-304
- Fiogbe, E., Carnavale, B. F., Takahashi, A. C. M. Exercise training in older adults, what effects on muscle force control? A systematic review of randomized clinical trials. *Arch Gerontol Geriatr.* 2019. 83:138-150
- Gheysen, F., Poppe, L., DeSmet, A., Swinnen, S., Cardon, G., De Bourdeaudhuij, I., Chastin, S., Fias, W. Physical activity to improve cognition in older adults: can physical activity programs enriched with cognitive challenges enhance the effects? A systematic review and meta-analysis. *Int J Behav Nutr Phys Act.* 2018. 15:63
- Gillespie, L. D., Robertson, M. C., Gillespie, W. J., Sherrington, C., Gates, S., Clemson, L. M., Lamb, E. S. Interventions for preventing falls in older people living in the community. *Cochrane Database Syst Rev.* 2012. 9:CD007146
- Gray, M. S., McKay, A. H. Nettlefold, R. L., Douglas, M., Heather M., Patti-Jean, N., Sims-Gould, J. Physical activity is good for older adults—but is programme implementation being overlooked? A systematic review of intervention studies that reported frameworks or measures of implementation. *Br J Sports Med.* 2021. 55:84-91
- Guadagnin, E. C., da Rocha, E. S., Duysens, J., Carpes, F. P. Does physical exercise improve obstacle negotiation in the elderly? A systematic review. *Arch Gerontol Geriatr.* 2016. 64:138-145
- Guirguis-Blake, J. M., Michael, Y. L., Perdue, L. A., Coppola, E. L., Beil, T. L., Thompson, J. H. Interventions to Prevent Falls in Community-Dwelling Older Adults: A Systematic Review for the U.S. Preventive Services Task Force. *Agency for Healthcare Research and Quality.* 2018. 159:1-261.
- Guo, W., Zang, M., Klich, S., Kawczynski, A., Smoter, M., Wang, B. Effect of Combined Physical and Cognitive Interventions on Executive Functions in OLDER Adults: A Meta-Analysis of Outcomes. *Int J Environment Res Public Health.* 2020. 17:1-19
- Hewston, P., Kennedy, C. C., Ioannidis, G., Marr, S., Santaguida, P., Santesso, N., Papaioannou, A., Borhan, S,, Merom, D,, Thabane, L., Bray, S. Effects of dance on cognitive function in older adults: A systematic review and meta-analysis. *Age Ageing.* 2021. 50:1084-1092
- Hindin, S. B., Zelinski, E. M. Extended practice and aerobic exercise interventions benefit untrained cognitive outcomes in older adults: a meta-analysis. *J Am Geriatr Soc.* 2012. 60:136-41
- Howe, T. E., Shea, B., Dawson, L. J., Downie, F., Murray, A., Ross, C., Harbour, T. R., Caldwell, M. L., Creed, G. Exercise for preventing and treating osteoporosis in postmenopausal women. *Cochrane Database Syst Rev.* 2011. 6:CD000333
- Hoy, O, S., Josefine, P., Michaela, K., Aaron, M. H. Effects of yoga-based interventions on cognitive function in healthy older adults: A systematic review of randomized controlled trials. *Complement Ther Med.* 2021. 58:1-8
- Hvid, G. L., Harwood, L. D., Eskildsen, F. S., Dalgas, U. A Critical Systematic Review of Current Evidence on the Effects of Physical Exercise on Whole/Regional Grey Matter Brain Volume in Populations at Risk of Neurodegeneration. *Sports Med.* 2021. 51:1651-1671
- Jmker-Hemink, V. E., Dijxhoorn, D. N., Briseno Ozumbilla, C. M., Wanten, G. J., van den Berg, M. G. Effective elements of home-delivered meal services to improve energy and protein intake: A systematic review. *Nutrition.* 2020. 69:110537
- James, E., Nichols, S., Goodall, S., Hicks, M. K. O'Doherty, F. A. The influence of resistance training on neuromuscular function in middle-aged and older adults: A systematic review and meta-analysis of randomised controlled trials. *Exp Gerontol.* 2021. 149:111320
- Kato, M., Nihei F.G., Hotta, K., Tsukamoto, T., Kurita, Y., Kubo, A., Takagi, H. The Efficacy of Stretching Exercises on Arterial Stiffness in Middle-Aged and Older Adults: A Meta-Analysis of Randomized and Non-Randomized Controlled Trials. *Int J Environment Res Public Health.* 2020. 17:1-15
- Kendrick, D., Kumar, A., Carpenter, H., Zijlstra, G. A., Skelton, D. A., Cook, J. R., Stevens, Z., Belcher, C. M., Haworth, D., Gawler, S. J., Gage, H., Masud, T., Bowling, A., Pearl, M., Morris, R. W. Exercise for reducing fear of falling in older people living in the community. *Cochrane Database Syst Rev.* 2014. 11:CD009848
- Keogh, J. W. L., O'Reilly, S., O'Brien, E., Morrison, S., Kavanagh, J. J. Can Resistance Training Improve Upper Limb Postural Tremor, Force Steadiness and Dexterity in Older Adults? A Systematic Review. *Sports Med.* 2019. 49:1199-1216
- Kheirouri, S., Alizadeh, M. MIND diet and cognitive performance in older adults: a systematic review. *Crit Rev Food Sci Nutr.* 2021. 1-19
- Klimova, B., Dostalova, R. The impact of physical activities on cognitive performance among healthy older individuals. *Brain Sciences.* 2020. 10:1-14
- Klimova, B., Novotny, M., Schlegel, P., Valis, M. The Effect of Mediterranean Diet on Cognitive Functions in the Elderly Population. *Nutrients.* 2021. 13:2067-2067
- Lee, S. H., Yu, S. Effectiveness of multifactorial interventions in preventing falls among older adults in the community: A systematic review and meta-analysis. *Int J Nurs Stud.* 2020. 106:103564
- Li, N., Hou, Z., Tan, J., Liu, T., Liu, L., Gao, L., Wang, L. Nutrition and exercise interventions could ameliorate age-related cognitive decline: a meta-analysis of randomized controlled trials. *Aging Clinical and Experimental Research.* 2021. 33:1799-1809
- Liu, C. J., Latham, N. Adverse events reported in progressive resistance strength training trials in older adults: 2 sides of a coin. *Arch Phys Med Rehabil.* 2010. 91:1471-3
- Ludyga, S., Gerber, M., Puhse, U., Looser, V. N., Kamijo, K. Systematic review and meta-analysis investigating moderators of long-term effects of exercise on cognition in healthy individuals. *Nat Hum Behav.* 2020. 4:603-612.
- McBean, L., O'Reilly, S. Diet quality interventions to prevent neurocognitive decline: a systematic review and meta-analysis. 2021. *Eur J Clin Nutr.* Online ahead of print.
- McEvoy, C. T., Leng, Y., Peeters, G. M., Kaup, A. R., Allen, I. E., Yaffe, K. Interventions involving a major dietary component improve cognitive function in cognitively healthy adults: a systematic review and meta-analysis. *Nutr Res.* 2019. 66:1-12
- McSween, M. P., Coombes, J. S., MacKay, C. P., Rodriguez, A. D., Erickson, K. I., Copland, D. A., McMohon, L, K. The Immediate Effects of Acute Aerobic Exercise on Cognition in Healthy Older Adults: A Systematic Review. *Sports Med.* 2019. 49:67-82
- Meng, X., Li, G., Jia, Y., Liu, Y., Shang, B., Liu, P., Bao, X., Chen, L. Effects of dance intervention on global cognition, executive function and memory of older adults: a meta-analysis and systematic review. *Aging Clin Exp Res.* 2020. 32:7-19
- Messecar, D. C. Review: several interventions reduce fear of falling in older people living in the community. *Evidence Based Nursing.* 2008. 11:21-21
- Olanrewaju, O., Kelly, S., Cowan, A., Brayne, C., Lafortune, L. Physical Activity in Community Dwelling Older People: A Systematic Review of Reviews of Interventions and Context. *PLoS One.* 2016. 11:e0168614
- Orssatto, B. R. L., Bezerra, S. E., Shield, J. A., Trajano, S. G. Is power training effective to produce muscle hypertrophy in older adults? A systematic review and meta-analysis. *Appl Physiol Nutr Metab.* 2020. 45:1031-1040
- Pai, L. W., Chen, W. The effectiveness of physical leisure time activities on the older adult's physical functioning: A Systematic Review. *JBI Lib Syst Rev.* 2011. 9:1-19
- Pedroso, R. V., Fraga, F. J., Ayán, C., Carral, J. M. C., Scarpari, L., Santos-Galduróz, R. F. Effects of physical activity on the P300 component in elderly people: a systematic review. *Psychogeriatrics.* 2017. 17:479-487
- Petrov, E. M., Lee, E. R., Hoffmann, M. C. Aerobic physical activity to improve memory and executive function in sedentary adults without cognitive impairment: A systematic review and meta-analysis. *Prev Med Rep.* 2021. 23:101496
- Pierson, K., Maloney, M., Bavuso, A., Dowling, Kathryn, K., Tenzin, W., Michelle E. A review of the impact of exercise on fall rates among community-dwelling older adults. *J Am Assoc Nurse Pract.* 2021. 34: 247-251
- Saez de Asteasu, M. L., Martinez-Velilla, N., Zambom-Ferraresi, F., Casas-Herrero, A., Izquierdo, M. Role of physical exercise on cognitive function in healthy older adults: A systematic review of randomized clinical trials. *Ageing Res Rev.* 2017. 37:117-134
- Sanders, L. M. J., Hortobagyi, T., la Bastide-van Gemert, S., van der Zee, E. A., van Heuvelen, M. J. G. Dose-response relationship between exercise and cognitive function in older adults with and without cognitive impairment: A systematic review and meta-analysis. *PLoS One.* 2019. 14:e0210036
- Schattin, A., Baur, K., Stutz, J., Wolf, P., de Bruin, E. D. Effects of Physical Exercise Combined with Nutritional Supplements on Aging Brain Related Structures and Functions: A Systematic Review. *Front Aging Neurosci.* 2016. 8:161
- Sherrington, C., Fairhall, N. J., Wallbank, G. K., Tiedemann, A., Michaleff, Z. A., Howard, K., Clemson, L., Hopewell, S., Lamb, E. S. Exercise for preventing falls in older people living in the community. *Cochrane Database Syst Rev.* 2019. 1:CD012424
- Sherrington, C., Fairhall, N., Wallbank, G., Tiedemann, A., Michaleff, Z. A., Howard, K., Clemson, L., Hopewell, S., Lamb, S. 2020. Exercise for preventing falls in older people living in the community: an abridged Cochrane systematic review *Br J Sports Med.* 54(15): 885-891.
- Sibley, M. K., Thomas, M. S., Veroniki, A. A., Rodriques, M., Hamid, S. J., Lachance, C. C., Cogo, E., Khan, A. P., Riva, J. J., Thavorn, K., MacDonald, H., Holroyd-Leduc, J., Feldman, F., Kerr, D. G., Jaglal, B. S., Straus, E. S., Tricco, C. A. Comparative effectiveness of exercise interventions for preventing falls in older adults: A secondary analysis of a systematic review with network meta-analysis. *Exp Gerontol.* 2021. 143:111151
- Sprague, B. N., Freed, S. A., Webb, C. E., Phillips, C. B., Hyun, J., Ross, L. A. The impact of behavioral interventions on cognitive function in healthy older adults: A systematic review. *Ageing Res Rev.* 2019. 52:32-52
- Turner, D. T., Hu, M. X., Generaal, E., Bos, D., Ikram, M. K., Heshmatollah, A., Fani, L., Ikram, A. M., Penninx, H. J. W. B., Cuijpers, P. Physical Exercise Interventions Targeting Cognitive Functioning and the Cognitive Domains in Nondementia Samples: A Systematic Review of Meta-Analyses. *J Geriatr Psychiatry Neurol.* 2020. 34:91-101.
- Vanderlinden, J., Boen, F., van Uffelen, J. G. Z. Effects of physical activity programs on sleep outcomes in older adults: a systematic review. *Int J Behav Nutr Phys Act.* 2020. 17:11
- Vaportzis, E., Niechcial, M. A., Gow, A. J. A systematic literature review and meta-analysis of real-world interventions for cognitive ageing in healthy older adults. *Ageing Res Rev.* 2019. 50:110-130
- Veazie, S., Gilbert, J., Winchell, K., Paynter, R., Guise, J. M. Addressing social isolation to improve the health of older adults: a rapid review. *Agency for Healthcare Research and Quality.* 2019. 19-EHC009-EF:1-19.
- Walton, K., do Rosario, V. A., Pettingill, H., Cassimatis, E., Charlton, K. The impact of home-delivered meal services on the nutritional intake of community living older adults: a systematic literature review. *J Hum Nutr Diet.* 2020. 33:38-47
- Weber, M., Schnorr, T., Morat, T., Morat, M., Donath, L. Effects of mind-body interventions involving meditative movements on quality of life, depressive symptoms, fear of falling and sleep quality in older adults: A systematic review with meta-analysis. *Int J Environment Res Public Health.* 2020. 17:1-22
- Weber, M., Schnorr, T., Morat, T., Morat, M., Donath, L. Effects of mind-body interventions involving meditative movements on quality of life, depressive symptoms, fear of falling and sleep quality in older adults: A systematic review with meta-analysis. *Int J Environment Res Public Health.* 2020. 17:1-2
- Winser, S. J., Chan, H. T. F., Ho, L., Chung, L. S., Ching, L. T., Felix, T. K. L., Kannan, P. Dosage for cost-effective exercise-based falls prevention programs for older people: A systematic review of economic evaluations. *Ann Phys Rehabil Med.* 2020. 63:69-80
- Young, J., Angevaren, M., Rusted, J., Tabet, N. Aerobic exercise to improve cognitive function in older people without known cognitive impairment. *Cochrane Database Syst Rev.* 2015. 4:CD005381
- Zang, Ming, Guo, Wei, Wang, Biye, Klich, Sebastian, Kawczynski, Adam, Smoter, Malgorzata 2020. Effect of combined physical and cognitive interventions on executive functions in older adults: A meta-analysis of outcomes *Int J Environment Res Public Health*, 17(17): 1-19.
- Zhang, C., Luo, J., Yuan, Changzheng, Ding, D. Vitamin B12, B6, or Folate and Cognitive Function in Community-Dwelling Older Adults: A Systematic Review and Meta-Analysis. *Journal of Alzheimer's Disease.* 2020. 77:781-794
- Zheng, G., Ye, B., Zheng, Y., Xiong, Z., Xia, R., Qiu, P., Tao, J., Chen, L. The effects of exercise on the structure of cognitive related brain regions: A meta-analysis of functional neuroimaging data. *Int J Neurosci.* 2018. 129:406-415.
- Zhu, X., Yin, S., Lang, M., He, R., Li, J. The more the better? A meta-analysis on effects of combined cognitive and physical intervention on cognition in healthy older adults. *Ageing Res Rev.* 2016. 31:67-79

**Excluded Studies: Published before 2010 (n=15)**

- Baker, M. K., Atlantis, E., Fiatarone Singh, M. A. Multi-modal exercise programs for older adults. *Age Ageing.* 2007. 36:375-81
- Donald, F. Review: exercise interventions improve functional and physical performance but not activities of daily living in older adults. *Evid Based Nurs.* 2009. 12:119-119
- Dudet, M. E. Importance of diet and physical exercise in sarcopenia prevention. *Rev Espanola de Nutr Comunitaria.* 2001. 7:86-91
- Heyn, P. C., Johnson, K. E., Kramer, A. F. Endurance and strength training outcomes on cognitively impaired and cognitively intact older adults: a meta-analysis. *J Nutr Health Aging.* 2008. 12:401-9
- Kelley, G. A., Kelley, K. S., Hootman, J. M., Jones, D. L. Exercise and health-related quality of life in older community-dwelling adults: A meta-analysis of randomized controlled trials. *J Appl Gerontol.* 2009. 28:369-394
- Keogh, J. W., Kilding, A., Pidgeon, P., Ashley, L., Gillis, D. Physical benefits of dancing for healthy older adults: a review. *J Aging Phys Act.* 2009. 17:479-500
- Latham, N. K., Bennett, D. A., Stretton, C. S., Anderson, C. A. Systematic review of progressive resistance training in older adults. *J Geriatr Phys Ther.* 2002. 25:28-29
- Latham, N., Anderson, C., Bennett, D., Stretton, C. Progressive resistance strength training for physical disability in older people. *Cochrane Database Syst Rev.* 2003. 2:CD002759
- Liu, C. J., Latham, N. K. Progressive resistance strength training for improving physical function in older adults. *Cochrane Database Syst Rev.* 2009. 3:CD002759
- McClure, R., Turner, C., Peel, N., Spinks, A., Eakin, E., Hughes, K. Population-based interventions for the prevention of fall-related injuries in older people. *Cochrane Database Syst Rev.* 2005. 1:CD004441
- Medical Advisory Secretariat. Prevention of falls and fall-related injuries in community-dwelling seniors: an evidence-based analysis. *Ontario Health Technology Assessment Series.* 2008. 8:1-78
- Orr, R., Raymond, J., Fiatarone Singh, M. Efficacy of progressive resistance training on balance performance in older adults: a systematic review of randomized controlled trials. *Sports Med.* 2008. 38:317-43
- Sayers, S. P. Resistance training in older adults: the importance of muscle power and speed of movement. *Am J Recreat Ther.* 2005. 4:21-26
- Sherrington, C., Lord, S. R., Finch, C. F. Physical activity interventions to prevent falls among older people: update of the evidence. *J Sci Med Sport.* 2004. 7:43-51
- Sohng, K. Y., Choi, J. H., Song, H. H., Moon, J. S. A meta-analysis of exercise programmes or preventing falls in older people. *Asian J Nurs Stud.* 2005. 8:3-15

**Excluded: Less than 80% of primary studies were intervention studies (n=5)**

- Burton, E., Farrier, K., Hill, K. D., Codde, J., Airey, P., Hill, A. M. Effectiveness of peers in delivering programs or motivating older people to increase their participation in physical activity: Systematic review and meta-analysis. *J Sports Sci.* 2018. 36:666-678
- Giné-Garriga, M., Vidal-Garcia, E., Gómara-Toldrà, N., Roman-Viñas, B., Roqué-Fíguls, M. Combined Effects of Diet and Exercise or Diet Alone to Improve Physical Function in Community-Dwelling Older Adults: A Systematic Review of the Literature. *Curr Nutr Rep.* 2015. 4:164-175
- Lim, S. E. R., Cox, N. J., Roberts, H. C. The Effectiveness of Volunteer-Led Physical Activity Interventions in Improving Health Outcomes for Community-Dwelling Older People: A Systematic Review. *Age Ageing.* 2020. 49:i30-i32
- Tan, Q. Y., Ibrahim, K., Lim, S. E. R., Cox, N. J., Roberts, H. C.. Volunteer-led physical activity interventions to improve health outcomes for community-dwelling older people: a systematic review. *Aging Clin Exp Res.* 2021. 33:843-853
- Valdes-Badilla, P. A., Gutierrez-Garcia, C., Perez-Gutierrez, M., Vargas-Vitoria, R., Lopez-Fuenzalida, A. Effects of Physical Activity Governmental Programs on Health Status in Independent Older Adults: A Systematic Review. *J Aging Phys Act.* 2019. 27:265-275.

**Excluded Studies: Less than 70% of primary studies meet inclusion criteria (n=32)**

- Apostolo, J., Cooke, R., Bobrowicz-Campos, E., Santana, S., Marcucci, M., Cano, A., Vollenbroek-Hutten, M., Germini, F., D’Avanzo, B., Gwyther, H., Holland, C. Effectiveness of interventions to prevent pre-frailty and frailty progression in older adults: a systematic review. *JBI Database System Rev Implement Rep.* 2018. 16:140-232
- Beaudart, C., Dawson, A., Shaw, S. C., Harvey, N. C., Kanis, J. A., Binkley, N. Reginster, Y. J., Charpurlat, R., Chan, C. D., Bruyere, O. Nutrition and physical activity in the prevention and treatment of sarcopenia: systematic review. *Osteoporos Int.* 2017. 28:1817-1833
- Beckwee, D., Delaere, A., Aelbrecht, S., Baert, V., Beaudart, C., Bruyere, O., de Saint-Hubert, M., Bautmans, I. Exercise Interventions for the Prevention and Treatment of Sarcopenia. A Systematic Umbrella Review. *J Nutr Health Aging.* 2019. 23:494-502
- Clifford, T., Jeffries, O., Stevenson, E. J., Davies, K. A. B. The effects of vitamin C and E on exercise-induced physiological adaptations: a systematic review and Meta-analysis of randomized controlled trials. *Crit Rev Food Sci Nutr.* 2020. 60:3669-3679.
- Corregidor-Sanchez, I.S., Segura-Fragoso, A., Rodriguez-Hernandez, M., Jimenez-Rojas, C., Polonio-Lopez, B., Criado-Alvarez, J. J. Effectiveness of virtual reality technology on functional mobility of older adults: systematic review and meta-analysis. *Age Ageing.* 2021. 50:370-379
- Courel-Ibanez, J., Vetrovsky, T., Dadova, K., Pallares, J. G., Steffl, M. Health Benefits of beta-Hydroxy-beta-Methylbutyrate (HMB) Supplementation in Addition to Physical Exercise in Older Adults: A Systematic Review with Meta-Analysis. *Nutrients.* 2019. 11:03
- Dedeyne, L., Deschodt, M., Verschueren, S., Tournoy, J., Gielen, E. Effects of multi-domain interventions in (pre)frail elderly on frailty, functional, and cognitive status: a systematic review. *Clin Interv Aging.* 2017. 12:873-896
- El-Kotob, R., Ponzano, M., Chaput, J., Janssen, I., Kho, E. M., Poitras, J. V., Ross, R., Ross-White, A., Saunders, J. T., Giangregorio, M. L. Resistance training and health in adults: an overview of systematic reviews. *Appl Physiol Nutr Metab.* 2020. 45:S165-S179
- Fairhall, N., Sherrington, C., Clemson, L., Cameron, I. D. Do exercise interventions designed to prevent falls affect participation in life roles? A systematic review and meta-analysis. *Age Ageing.* 2011. 40:666-74
- Farlie, M. K., Robins, L., Haas, R., Keating, J. L., Molloy, E., Haines, T. P. Programme frequency, type, time and duration do not explain the effects of balance exercise in older adults: a systematic review with a meta-regression analysis. *Br J Sports Med.* 2019. 53:996-1002.
- Gavelin, M. H., Dong, C., Minkov, R., Bahar-Fuchs, A., Ellis, A. K., Lautenschlager, T. N., Mellow, L. M., Wade, T. A., Smith, E. A., Finke, C., Krohn, S., Lampit, A. Combined physical and cognitive training for older adults with and without cognitive impairment: A systematic review and network meta-analysis of randomized controlled trials. *Ageing Res Rev.* 2021. 66:101232
- Grande, D. G., Oliveria, B. C., Morelhao, K. P., Sherrington, C, Tiedemann, A., Pinto, Z. R,. Franco, R. M. Interventions Promoting Physical Activity Among Older Adults: A Systematic Review and Meta-Analysis. *Gerontologist.* 2020. 60:583-599
- Hart, P. D., Buck, D. J. The effect of resistance training on health-related quality of life in older adults: Systematic review and meta-analysis. *Health Promot Perspect.* 2019. 9:1-12
- Huang, Y., Liu, X. Improvement of balance control ability and flexibility in the elderly Tai Chi Chuan (TCC) practitioners: a systematic review and meta-analysis. *Arch Gerontol Geriatr.* 2015. 60:233-8
- Katigbak, C., Flaherty, E., Chao, Y. Y., Nguyen, T., Cheung, D., Yiu-Cho Kwan, R. A. Systematic Review of Culturally Specific Interventions to Increase Physical Activity for Older Asian Americans. *J Cardiovasc Nurs.* 2018. 33:313-321
- Klempel, N, Blackburn, E. N., McMullan, L. I., Wilson, J. J., Smith, L., Cunningham, C., O'Sullivan, R., Caserotti, P., Tully, A. M., The Effect of Chair-Based Exercise on Physical Function in Older Adults: A Systematic Review and Meta-Analysis. *Int J Environmen Res Public Health.* 2021. 18:1902
- Klempel, N, Blackburn, E. N., McMullan, L. I., Wilson, J. J., Smith, L., Cunningham, C., O'Sullivan, R., Caserotti, P., Tully, A. M., The Effect of Chair-Based Exercise on Physical Function in Older Adults: A Systematic Review and Meta-Analysis. *Int J Environmen Res Public Health.* 2021. 18:1902
- Liao, C. D., Tsauo, J. Y., Wu, Y. T., Cheng, C. P., Chen, H. C., Huang, Y. C. Chen, H., Liou, T. Effects of protein supplementation combined with resistance exercise on body composition and physical function in older adults: a systematic review and meta-analysis. *Am J Clin Nutr.* 2017. 106:1078-1091
- Liu, C. J., Latham, N. Can progressive resistance strength training reduce physical disability in older adults? A meta-analysis study. *Disabil Rehabil.* 2011. 33:87-97
- Martins, W. R., de Oliveira, R. J., Carvalho, R. S., de Oliveira Damasceno, V., da Silva V. Z., Silva, M. S. Elastic resistance training to increase muscle strength in elderly: a systematic review with meta-analysis. *Arch Gerontol Geriatr.* 2013. 57:8-15
- Oliveira, J. S., Sherrington, C., Amorim, A. B., Dario, A. B., Tiedemann, A. What is the effect of health coaching on physical activity participation in people aged 60 years and over? A systematic review of randomised controlled trials. *Br J Sports Med.* 2017. 51:1425-1432
- Pacheco, T. B. F., e Medeiros, C. S. P., e Oliveira, V. H. B., Vieira, E. R., e Cavalcanti, F. A. C.. Effectiveness of exergames for improving mobility and balance in older adults: a systematic review and meta-analysis. *Syst Rev.* 2020. 9:163
- Poscia, A., Milovanovic, S., La Milia, D. I., Duplaga, M., Grysztar, M., Landi, F., Moscato, U., Magnavita, N., Collamati, A., Ricciardi, W. Effectiveness of nutritional interventions addressed to elderly persons: umbrella systematic review with meta-analysis. *Eur J Public Health.* 2018. 28:275-283
- Roberts, C. E., Phillips, L. H., Cooper, C. L., Gray, S., Allan, J. L. Effect of Different Types of Physical Activity on Activities of Daily Living in Older Adults: Systematic Review and Meta-Analysis. *J Aging Phys Act.* 2017. 25:653-670
- Sarabon, N., Kozinc, Z., Lofler, S., Hofer, C. Resistance exercise, electrical muscle stimulation, and whole-body vibration in older adults: Systematic review and meta-analysis of randomized controlled trials. *J Clin Med.* 2020. 9:1-21
- Shvedko, A., Whittaker, A. C., Thompson, J. L., Greig, C. A. Physical activity interventions for treatment of social isolation, loneliness or low social support in older adults: A systematic review and meta-analysis of randomised controlled trials. *Psych Sport Exerc.* 2018. 34:128-137
- Stewart, V. H., Saunders, D. H., Greig, C. A. Responsiveness of muscle size and strength to physical training in very elderly people: a systematic review. *Scand J Med Sci Sports.* 2014. 24:e1-10
- Teng, B, Gomersall, R. G., Hatton, A., Brauer, G.B. Combined group and home exercise programmes in community-dwelling falls-risk older adults: Systematic review and meta-analysis. *Physiother Res Int.* 2020. 25:e1839
- Thomas, D. K., Quinn, M. A., Saunders, D. H., Greig, C. A. Protein Supplementation Does Not Significantly Augment the Effects of Resistance Exercise Training in Older Adults: A Systematic Review. *J Am Med Dir Assoc.* 2016. 17:959.e1-9
- Vasquez-Araneda, E., Solis-Vivanco, R. I., Mahecha-Matsudo, S., Zapata-Lamana, R., Cigarroa, I. Characteristics of Physical Exercise Programs for Older Adults in Latin America. A Systematic Review of Randomized Controlled Trials. *Int J Environment Res Public Health.* 18:1-27
- Young, K., Bunn, F., Trivedi, D., Dickinson, A. Nutritional education for community dwelling older people: a systematic review of randomised controlled trials. *Int J Nurs Stud*. 48:751-80

Zheng, L., Li, G., Wang, X., Yin, H., Jia, Y., Leng, M., Li, H., Chen, L. Effect of exergames on physical outcomes in frail elderly: a systematic review. *Aging Clinical Exp Res.* 2020. 32:2187-2200
