# Supplementary material for: Community-based group physical activity and/or nutrition interventions to promote mobility in older adults: an umbrella review
Source: BMC Geriatr. 2022 Jun 29;22:539. doi: 10.1186/s12877-022-03170-9 (PMC9241281; doi:10.1186/s12877-022-03170-9)
Supplement: Supplementary file 3 — Additional file 3. AMSTAR 2 Critical Appraisal Results. [file 12877_2022_3170_MOESM3_ESM.docx]

**Additional file 3: AMSTAR 2 Critical Appraisal Results**

| Author | 1 | 2 | 3 | 4 | 5 | 6 | 7 | 8 | 9a | 9b | 10 | 11a | 11b | 12 | 13 | 14 | 15 | 16 | Overall |
| --- | --- | --- | --- | --- | --- | --- | --- | --- | --- | --- | --- | --- | --- | --- | --- | --- | --- | --- | --- |
| Antoniak, 2017 | Yes | Yes | No | Partial | No | Yes | No | Yes | Yes | N/A | No | Yes | N/A | Yes | Yes | Yes | Yes | Yes | ◼ |
| Borde, 2015 | Yes | No | No | No | Yes | No | No | No | Yes | N/A | No | No | N/A | No | No | Yes | No | Yes | ◼ |
| Bouaziz, 2016 | No | No | No | Partial | Yes | No | No | No | No | No | No | N/A | N/A | N/A | No | Yes | N/A | Yes | ◼ |
| Bouaziz, 2017 | Yes | No | No | Partial | Yes | Yes | No | No | Yes | Yes | No | N/A | N/A | N/A | Yes | Yes | N/A | Yes | ◼ |
| Bouaziz, 2018 | No | No | No | No | Yes | No | No | No | Yes | N/A | No | Yes | N/A | No | No | Yes | Yes | Yes | ◼ |
| Bruderer-Hofstetter, 2018 | Yes | Yes | No | Partial | Yes | Yes | No | Yes | Yes | N/A | No | Yes | N/A | Yes | Yes | Yes | Yes | Yes | ◼ |
| Bueno de Souza, 2018 | Yes | No | No | No | Yes | No | No | Partial | Yes | N/A | No | Yes | N/A | Yes | Yes | Yes | No | Yes | ◼ |
| Bullo, 2015 | No | No | No | No | Yes | Yes | No | No | Partial | No | No | No | No | No | No | No | No | Yes | ◼ |
| Bullo, 2018 | Yes | No | Yes | Partial | Yes | Yes | No | Partial | Yes | Yes | No | No | No | No | No | No | No | Yes | ◼ |
| Chase, 2017 | Yes | No | No | Partial | No | Yes | No | No | Yes | Yes | No | Yes | No | Yes | Yes | Yes | Yes | Yes | ◼ |
| da Rosa Orssatto, 2019 | Yes | Partial | No | Partial | Yes | No | Yes | Yes | Yes | N/A | No | Yes | N/A | Yes | Yes | Yes | Yes | No | ◼ |
| Devries, 2014 | Yes | No | Yes | Partial | No | No | No | Yes | Yes | N/A | No | Yes | N/A | No | Yes | Yes | Yes | Yes | ◼ |
| Ebner, 2021 | Yes | No | No | No | Yes | Yes | No | No | Yes | N/A | No | Yes | N/A | No | No | No | No | Yes | ◼ |
| Elboim-Gabyzon, 2021 | No | Yes | No | No | Yes | Yes | No | Yes | Yes | Yes | No | N/A | N/A | N/A | No | Yes | N/A | Yes | ◼ |
| Fernandez-Arguelles, 2015 | Yes | Partial | No | Partial | Yes | Yes | No | Yes | Yes | Yes | No | N/A | N/A | N/A | No | Yes | N/A | Yes | ◼ |
| Fernández-Rodríguez, 2020 | Yes | Yes | No | Partial Yes | Yes | Yes | No | Partial Yes | Yes | N/A | No | Yes | N/A | Yes | Yes | Yes | Yes | Yes | ◼ |
| Finger, 2015 | Yes | No | No | Partial | Yes | Yes | No | No | No | N/A | No | Yes | N/A | No | No | Yes | No | Yes | ◼ |
| Frost, 2017 | Yes | Partial | No | Partial | Yes | No | No | Yes | Yes | N/A | No | Yes | N/A | No | Yes | Yes | No | Yes | ◼ |
| Gade, 2018 | Yes | Yes | Yes | Yes | Yes | Yes | Partial | Yes | Yes | N/A | Yes | N/A | N/A | N/A | Yes | Yes | N/A | Yes | ◼ |
| Garcia-Hermoso, 2020 | Yes | Yes | No | Partial | Yes | No | No | Partial | Yes | N/A | No | Yes | N/A | Yes | Yes | Yes | Yes | Yes | ◼ |
| Grässler, 2021 | No | Yes | No | No | Yes | Yes | No | No | Yes | Yes | No | N/A | N/A | N/A | No | Yes | N/A | Yes | ◼ |
| Hanach, 2019 | Yes | No | No | Partial | No | No | No | Partial | Yes | N/A | No | Yes | N/A | No | Yes | No | No | Yes | ◼ |
| Hortobagyi, 2015 | Yes | No | No | Partial | Yes | No | No | Partial | Yes | N/A | No | No | N/A | No | No | No | No | Yes | ◼ |
| Hou, 2019 | Yes | No | No | Partial | Yes | Yes | No | Partial | Yes | N/A | No | Yes | N/A | No | No | Yes | Yes | Yes | ◼ |
| Howe, 2011 | Yes | Yes | Yes | Yes | Yes | Yes | Yes | Yes | Yes | N/A | Yes | Yes | N/A | Yes | Yes | Yes | Yes | Yes | ◼ |
| Hurst, 2019 | Yes | Yes | No | Partial | Yes | Yes | No | Yes | Yes | Partial | No | Yes | Yes | No | Yes | Yes | Yes | Yes | ◼ |
| Hwang,2015 | No | No | No | No | No | No | No | Partial | No | No | No | N/A | N/A | N/A | No | No | N/A | No | ◼ |
| Katsoulis, 2019 | Yes | Yes | No | Partial | Yes | No | No | Partial | Yes | No | No | N/A | N/A | N/A | No | No | N/A | No | ◼ |
| King, 2016 | Yes | No | Yes | Partial | Yes | No | Partial | Yes | Yes | Yes | No | N/A | N/A | N/A | Yes | Yes | N/A | No | ◼ |
| Labott, 2019 | Yes | No | No | Partial | Yes | No | No | Partial | Yes | N/A | No | Yes | N/A | No | No | Yes | Yes | Yes | ◼ |
| Lesinski, 2015 | Yes | No | Yes | Partial | Yes | No | No | Partial | Yes | N/A | No | Yes | N/A | No | No | No | No | Yes | ◼ |
| Leung, 2011 | No | No | No | Partial | Yes | No | No | No | Yes | N/A | No | No | N/A | No | No | No | No | No | ◼ |
| Levin, 2017 | Yes | No | Yes | Partial | Yes | No | Yes | Yes | No | No | No | N/A | N/A | N/A | Yes | Yes | N/A | Yes | ◼ |
| Liberman, 2017 | Yes | No | No | No | No | No | No | Yes | Yes | N/A | No | N/A | N/A | N/A | No | No | N/A | Yes | ◼ |
| Liu, 2010 | Yes | No | No | No | No | Yes | No | Yes | No | No | No | N/A | N/A | N/A | No | Yes | N/A | No | ◼ |
| Liu, 2017 | Yes | No | No | Partial | Yes | No | No | Yes | Yes | N/A | No | Yes | N/A | No | No | Yes | No | Yes | ◼ |
| Liu, 2020 | Yes | Partial | Yes | No | Yes | No | No | Partial | Yes | N/A | No | Yes | N/A | No | No | Yes | No | Yes | ◼ |
| Loureiro, 2021 | No | No | No | No | Yes | No | No | Yes | Yes | N/A | No | N/A | N/A | N/A | No | No | N/A | Yes | ◼ |
| Martin, 2013 | Yes | No | No | No | Yes | No | No | No | Yes | N/A | No | N/A | N/A | N/A | Yes | Yes | N/A | Yes | ◼ |
| Martins, 2018 | Yes | No | No | Yes | No | No | No | Yes | Yes | No | No | N/A | N/A | N/A | No | No | N/A | Yes | ◼ |
| Meereis-Lemos, 2020 | Yes | Yes | No | No | Yes | No | No | No | Yes | N/A | No | Yes | N/A | No | No | Yes | No | Yes | ◼ |
| Montero, 2016 | No | No | No | Partial | Yes | Yes | No | Partial | N/A | Yes | No | N/A | Yes | Yes | Yes | Yes | Yes | Yes | ◼ |
| Moore, 2016 | Yes | No | No | Partial | Yes | No | No | Yes | Yes | Yes | No | N/A | N/A | N/A | Yes | Yes | N/A | No | ◼ |
| Moran, 2018 | Yes | No | No | Partial | No | No | No | Partial | Partial | No | No | Yes | N/A | No | No | No | No | Yes | ◼ |
| Nicolson, 2021 | No | Yes | No | Partial Yes | Yes | Yes | No | Partial Yes | Yes | N/A | No | Yes | N/A | Yes | Yes | Yes | Yes | Yes | ◼ |
| Plummer, 2015 | Yes | No | Yes | Partial | Yes | Yes | No | Yes | Yes | Yes | No | Yes | N/A | No | No | No | Yes | Yes | ◼ |
| Qi, 2018 | Yes | Yes | No | Partial | Yes | No | No | Yes | Yes | Yes | No | N/A | N/A | N/A | Yes | Yes | N/A | No | ◼ |
| Raymond, 2013 | Yes | No | Yes | Partial | No | Yes | No | Yes | Yes | N/A | No | Yes | N/A | No | Yes | No | No | Yes | ◼ |
| Rodrigues-Krause, 2019 | Yes | No | No | Partial | No | No | No | Yes | Yes | No | No | N/A | N/A | N/A | Yes | No | N/A | Yes | ◼ |
| Roland, 2011 | Yes | No | No | Partial | No | No | No | Yes | Yes | Yes | No | N/A | N/A | N/A | No | No | N/A | No | ◼ |
| Sivaramakrishnan, 2019 | Yes | Yes | No | Partial | Yes | Yes | No | Partial | Yes | N/A | No | Yes | N/A | No | Yes | Yes | No | Yes | ◼ |
| Stares, 2020 | Yes | No | No | Partial | No | Yes | No | Partial | Yes | N/A | No | N/A | N/A | N/A | No | No | N/A | Yes | ◼ |
| Stathokostas, 2012 | Yes | No | No | Partial | Yes | Yes | No | Yes | Yes | Yes | No | N/A | N/A | N/A | Yes | Yes | N/A | Yes | ◼ |
| Straight, 2016 | Yes | No | No | No | No | Yes | No | Partial | No | N/A | No | Yes | N/A | No | No | No | Yes | Yes | ◼ |
| Ten Haaf, 2018 | No | No | No | No | Yes | Yes | No | Partial | Yes | N/A | No | Yes | N/A | No | No | Yes | Yes | Yes | ◼ |
| Tschopp, 2011 | Yes | No | No | Partial | Yes | Yes | No | Partial | Partial | Partial | No | Yes | Yes | No | Yes | Yes | No | Yes | ◼ |
| Van Abbema, 2015 | Yes | No | No | Partial | Yes | Yes | No | Yes | Yes | N/A | No | Yes | N/A | Yes | Yes | Yes | Yes | Yes | ◼ |
| Vetrovsky, 2019 | Yes | Yes | No | Partial | Yes | Yes | No | Yes | Yes | N/A | No | N/A | N/A | N/A | Yes | No | N/A | Yes | ◼ |
| Waller, 2016 | Yes | No | No | Partial | Yes | Yes | No | Yes | Yes | N/A | No | Yes | N/A | Yes | Yes | Yes | Yes | Yes | ◼ |
| Wang, 2021 | No | Yes | No | No | Yes | Yes | No | Partial Yes | Yes | N/A | No | Yes | N/A | Yes | No | Yes | Yes | Yes | ◼ |
| Wirth, 2020 | Yes | Yes | No | Partial Yes | Yes | Yes | No | Partial Yes | Yes | N/A | No | Yes | N/A | Yes | Yes | Yes | Yes | Yes | ◼ |
| Yang, 2019 | Yes | No | No | No | Yes | Yes | No | Partial | Yes | N/A | No | Yes | N/A | No | Yes | No | No | Yes | ◼ |
| Note: ◼ = critically low quality; ◼ = low quality; ◼ = moderate quality; ◼ = high quality | | | | | | | | | | | | | | | | | | | |
