# Supplementary material for: Community-based group physical activity and/or nutrition interventions to promote mobility in older adults: an umbrella review
Source: BMC Geriatr. 2022 Jun 29;22:539. doi: 10.1186/s12877-022-03170-9 (PMC9241281; doi:10.1186/s12877-022-03170-9)
Supplement: Supplementary file 4 — Additional file 4. Aerobic Outcomes. [file 12877_2022_3170_MOESM4_ESM.docx]

**Additional file 4: Aerobic Outcomes**

| **Study** | **Intervention/Comparison Description** | **Measure & Unit** | **Meta-Analysis Results**  **(Mean difference, 95% CI)** | **Narrative Results** | **Heterogeneity** |
| --- | --- | --- | --- | --- | --- |
| **Resistance exercise** | | | | | |
| Raymond 2013 | Lower limb high intensity progressive RT with/without upper limb, or trunk strengthening. Must be land based, within defined %1RM ranges, excluding high velocity power training, or combinations of other exercise. Comparison: Low or moderate-intensity RT | VO_2_ peak (measured or predicted) | 0.93 mL/kg/min (-0.69, 2.55) | - | NR |
| **Aerobic exercise** | | | | | |
| Bouaziz 2017 | Supervised (class or small group) AT, defined as any exercise involving movement of large muscle groups for a period (i.e., treadmill walking/running, walking, cycling, rowing, or dancing). No threshold set for frequency, duration, or intensity. Comparison: NR | VO_2_ peak (graded exercise test) | - | In 13 RCTs, VO_2_ peak significantly increased in intervention vs. control; in 9 single-group studies there was a significant improvement post- intervention vs. baseline. One study found no difference. Range of change +6.5 to +46.0%. | NR |
| Bouaziz 2018 | AT, defined as any activity that uses large muscle groups, can be maintained continuously, and is rhythmic in nature. No specific threshold for frequency, duration, and intensity. Only supervised programs were included. Comparison: 8/10 control groups usual daily activities, 2/10 other exercise. | VO_2_ peak | 1.56 mL/kg/min (0.90, 2.23) | - | I^2^ = 83.5% |
|  |  | VO_2_ peak (*healthy participants)* | 1.72 mL/kg/min (0.34, 3.10) | - | I^2^ = 69.1% |
|  |  | VO_2_ peak (*unhealthy participants)* | 1.47 mL/kg/min (0.60, 2.34) | - | I^2^ = 91.4% |
| Bullo 2018 | Supervised or unsupervised Nordic walking. Comparison: sedentary group, walking, and RT. | Aerobic capacity *vs. sedentary group* | SMD = 0.92 (0.56, 1.28) | - | NR |
|  |  | Aerobic capacity *vs. walking* | SMD = -0.21 (-0.64, 0.21) | - | NR |
|  |  | Aerobic capacity *vs. RT* | SMD = 0.75 (0.03, 1.47) | - | NR |
| Montero 2016 | AT defined as dynamic exercise involving a large muscle mass (e.g., running, cycling) lasting 3 weeks or more. Comparison: Post-intervention values compared to pre-intervention values. | VO_2_max | SMD = 0.89 (0.65, 1.12) | - | I^2^ = 0% |
|  |  | Qmax | SMD = 0.61 (0.37, 0.85) | - | I^2^ = 0% |
|  |  | Ca-VO_2_max | SMD = 0.38 (0.11, 0.66) | - | I^2^ = 26% |
| **Combined aerobic and resistance exercise** | | | | | |
| Bouaziz 2016 | Multi-modal exercise including AT, RT, balance, stability, flexibility, and/or coordination training. AT defined as exercise involving movement of large muscle groups for a period (e.g., walking, cycling, or rowing). RT defined as progressive training involving an increase in load over time without a specific intensity. Balance/stability training included exercise to increase one’s ability to maintain balance in the face of a threat to stability (e.g., specific balance exercises or Tai Chi). Comparison: criteria NR | VO_2_ peak (graded treadmill walking test) | - | VO_2_ peak was significantly improved in one study compared with the control group and in 2 studies when compared with baseline. Range of improvement was +10.0% to 20.0%. | NR |
| Hurst 2019 | At least one AT and RT group. AT defined as exercise involving large muscle groups in dynamic activities to increase in heart rate and energy expenditure. RT defined as muscle-strengthening activities working against or moving an external resistance (e.g., free weights, machines, elastic bands, body weight). Comparison: either (1) no-exercise control; (2) AT only; or (3) RT only. | VO_2_ peak *vs. no exercise* | 3.6 mL/kg/min (2.8, 4.4) | - | Small |
|  |  | VO_2_ peak *vs. AT only* | 0.8 mL/kg/min (0.2, 1.8) | - | Trivial |
|  |  | VO_2_ peak (*females only) vs. no exercise* | 2.1 mL/kg/min (0.3, 3.9) | - | Small |
| **Exercise and nutritional supplements** | | | | | |
| Stares 2020 | Cr supplementation with a physical training program. Comparison: placebo. | Endurance (e.g., V0_2_ peak, 1 mile walk test, ventilatory threshold) | - | No significant improvement in two of two studies | NR |
| **Mind-body exercise** | | | | | |
| Bueno de Souza 2018 | Mat Pilates with or without accessories. Comparison: No exercise | Cardiovascular fitness (pooled 6MWT, VO_2_ max) | SMD = 1.48 (0.42, 2.54) | - | I^2^ = 85% |
| Ebner 2021 | Yoga, Qi Gong, Tai Chi, Pilates. Comparison: Active and inactive controls | Endurance performance *vs. inactive control* | SMD = 0.44 (90% CI 0.25, 0.62) | - | I^2^ = 0% |
|  |  | Endurance performance *vs. active control* | SMD = 0.06 (90% CI -0.36, 0.48) | - | I^2^ = 69% |
| Roland 2011 | Yoga. Comparison: other exercise, non-exercise, or pre/post yoga groups | Lung capacity | - | Improved in 2 studies, effect size small and not significant | NR |
|  |  | Aerobic capacity (e.g., VO_2_ max) | - | Significantly improved in one study (very small effect size); no change in another study | NR |
| **General physical activity** | | | | | |
| Grässler 2021 | Physical training intervention (endurance, resistance, coordinative, or multimodal training) with a minimum of 4 weeks and 8 training sessions. Comparison: NR | VO_2_ max or peak | - | Significantly improved in 5/5 studies, effect size NR | High |
| **Dance** | | | | | |
| Rodrigues-Krause 2019 | Regular dance classes of any style for at least 2 weeks. Dance environments included dance studios and stage and/or dance ballrooms. Comparison: Non-exercising control groups and/or groups performing other types of exercise. | Cardiorespiratory fitness | - | All interventions increased aerobic fitness within and between groups; four studies found improvements like other exercise, such as AT or AT/RT. | NR |
| **Other exercise types** | | | | | |
| Bruderer-Hotstetter 2018 | Combined cognitive training and physical exercise interventions. The exercise component was planned, structured and repetitive and include at least one aspect of physical fitness. Interventions requiring simultaneous cognitive activity, such as exergames, dancing or Tai Chi were also included. Comparison: attention-controls or no intervention. | - | - | 2 studies found positive effects on cardiorespiratory fitness, and 3 found no effects | NR |
| Waller 2016 | Exercise in an aquatic environment with no limitation on the type of exercise. Comparison: Land exercise or no exercise | Aerobic power, *vs. control* | SMD = 1.98 (0.32, 3.64) | - | I^2^ = 93% |
|  |  | Aerobic power, *vs. land exercise* | SMD = 0.85 (-0.57, 2.27) | - | I^2^ = 92% |
| Note: 1RM = one-rep max; 6MWT = six-minute walking test; AT = aerobic exercise training; Ca-vO_2_max = arteriovenous oxygen difference at maximal exercise; Cr = creatine supplementation; kg = kilogram; min = minute; mL = milliliter; NR = not reported; Qmax = maximal cardiac output; RT = resistance training; SMD = standardized mean difference; VO_2_ max = maximal oxygen consumption | | | | | |
