# Supplementary material for: Community-based group physical activity and/or nutrition interventions to promote mobility in older adults: an umbrella review
Source: BMC Geriatr. 2022 Jun 29;22:539. doi: 10.1186/s12877-022-03170-9 (PMC9241281; doi:10.1186/s12877-022-03170-9)
Supplement: Supplementary file 5 — Additional file 5. Physical Function Outcomes. [file 12877_2022_3170_MOESM5_ESM.docx]

**Additional file 5: Physical Function Outcomes**

| **Study** | **Intervention/Comparison Description** | **Measure & Unit** | **Meta-Analysis Results**  **(Mean difference, 95% CI)** | **Narrative Results** | **Heterogeneity** |
| --- | --- | --- | --- | --- | --- |
| **Resistance exercise** | | | | | |
| da Rosa Orssatto 2019 | Fast vs. moderate-velocity lower limb RT | Combined functional capacity tests | SMD: 0.41 (0.18, 0.65) | - | I^2^ = 45% |
|  |  | TUG | SMD: 0.43 (-0.07, 0.94) | - | I^2^ = 48% |
|  |  | Chair stands | SMD: 0.58 (-0.24, 1.39) | - | I^2^ = 72% |
|  |  | SPPB | SMD: 0.52 (0.10, 0.94) | - | I^2^ = 0% |
| Hortobagyi 2015 | RT, defined as systematic series of exercises that cause muscles to work or hold against an applied force or weight, compared to no exercise. | Gait speed | SMD: 0.84 (0.52, 1.16) | - | I^2^ = 84% |
| Howe 2011 | Interventions designed to improve balance, in which participants exercise against an external force, or in response to an unexpected perturbation/stimulus. Strength training included RT and power training. Comparison: usual activities (such as attending recreational or educational activities or groups) that received the same attention (number of attendances at classes or contact with the research team) as the exercise group. | TUG | -4.30 s (-7.60, 1.00) | - | I^2^ = 0% |
|  |  | Gait speed | SMD: 0.25 (0.05, 0.46) | - | I^2^ = 0% |
| Katsoulis 2019 | Low (<50% 1RM), moderate or high (>70% 1RM) intensity power training. Post-intervention values compared to pre-intervention values. | Overall function *high intensity* | - | Pre-post changes: +17% | NR |
|  |  | Overall function *moderate intensity* | - | Pre-post changes: +12% | NR |
|  |  | Overall function *low intensity* | - | Pre-post changes: +10% | NR |
| Meereis-Lemos 2020 | Supervised RT at least twice a week for a minimum of 8 weeks. Comparison: no exercise control group. | TUG | -0.48 s (-1.10, 0.15) | - | I^2^ = 63% |
|  |  | 30s chair stand | 2.67 repetitions (1.21, 4.14) | - | I^2^ = 0% |
|  |  | Chair stand (5 rep) | - 0.80 s (-1.90, 0.30) | - | I^2^ = 15% |
| Liu 2017 | Progressive RT, in which one exerts an effort against an external resistance that is increased gradually as progress is made. Comparison: No intervention or attention control without any exercise components. | Gait speed (habitual) | SMD: 0.08 (-0.11, 0.26) | - | NR |
|  |  | TUG | SMD: -0.02 (-0.19, 0.15) | - | NR |
|  |  | Overall physical function | SMD: -0.07 (-0.26, 0.13) | - | NR |
| Nicolson 2021 | Therapeutic RT (contracting muscles against external force such as weights, resistance band, or body weight). Comparison: Usual care, no treatment, other exercise, pharmacotherapy, or health education | Performance based physical function *vs. no exercise* | SMD: -0.01 (-0.39, 0.38) | - | I^2^ = 6% |
| Raymond 2013 | Lower limb high intensity progressive RT with/without upper limb, or trunk strengthening. Must be land based, within defined %1RM ranges, excluding high velocity power training, or combinations of other exercise. Comparison: Low or moderate-intensity RT | Functional performance (various measures) | - | 2 studies reported reduced stair climb time with high vs moderate RT. No improvement was found for stair climb power, or overall physical performance. High intensity RT improved TUG scores but not 6MWT. | NR |
| Tschopp 2011 | Power training (moderate resistance and an ‘as fast as possible’ movement speed for at least the concentric phase of an exercise). Comparison: Conventional RT (high or moderate resistance and slow concentric movement) | Functional outcomes (various measures) | SMD: 0.32 (0.06, 0.57) | - | I^2^ = 0% |
|  |  | Self-reported function | SMD: 0.16 (−0.17, 0.49) | - | I^2^ = 0% |
| Van Abbema 2015 | Progressive RT. Comparison: usual activity or attention control (e.g., health, wellness, or driver education classes) | Preferred gait speed, *progressive RT only* | 0.13 m/s (0.09, 0.16) | - | I^2^ = 0% |
|  |  | Preferred gait speed, *progressive RT, and balance* | 0.02 m/s (-0.05, 0.10) | - | I^2^ = 67.8% |
| **Aerobic exercise** | | | | | |
| Bouaziz 2017 | Supervised (class or small group) AT, defined as any exercise involving movement of large muscle groups for a period of time (i.e., treadmill walking/running, walking, cycling, rowing, or dancing). No threshold set for frequency, duration, or intensity. Comparison: NR | Physical performance (various measures) | - | 3 RCTs and 2 non-RCTs all reported significant improvement and gain in physical performance 7.0 and 46.0% | NR |
| Bullo 2018 | Supervised or unsupervised Nordic walking. Comparison: Sedentary group, walking, and RT. | TUG *vs sedentary* | SMD = 0.30 (-0.28 to 0.88) | - | NR |
|  |  | TUG *vs walking* | SMD = 0.30 (-0.10 to 0.70) |  |  |
|  |  | TUG *vs RT* | SMD = 0.33 (-0.08 to 0.73) |  |  |
| Elboim-Gabyzon 2021 | High-intensity exercise (90–95% peak heart rate, 90% maximal oxygen uptake, at least 75% peak work rate) separated by periods of low to moderate-intensity or rest (e.g., walking/running, cycling).  Comparison: No treatment or other exercise | TUG | - | Demonstrated significant improvement in 3/3 studies, effect size NR | High |
|  |  | 6MWT | - | Improved in 1/3 studies, effect size NR |  |
| **Combined aerobic and resistance exercise** | | | | | |
| Bouaziz 2016 | Multi-modal exercise including AT, RT, balance, stability, flexibility, and/or coordination training. AT defined as exercise involving movement of large muscle groups for a period of time (e.g., walking, cycling, or rowing). RT defined as progressive training involving an increase in load over time without a specific intensity. Balance training included exercise to increase one’s ability to maintain balance with a threat to stability (e.g., specific balance exercises or Tai Chi). Comparison: Control criteria NR | Gait ability (various measures) | - | Significant improvement in gait performance was reported by 7 RCTs and 4 non-RCTs, with a gain in walk ability ranging from 7.2% to 40.0%. | NR |
|  |  | 6MWT | - | A significant improvement was measured in all studies (ranging from 1.0% to 41.8%). | NR |
| Chase 2017 | Supervised intervention involving RT and/or AT. 18 studies used RT only, the remainder used combined RT and AT. Comparison: NR | Composite measure of physical function | SMD: 0.62 (0.40, 0.84) | - | Potential publication bias |
| Hortobagyi 2015 | Interventions including 2+ types of exercise (RT, AT, balance, and functional training). Comparison: no exercise | Gait speed | SMD: 0.86 (0.50, 1.23) | - | I^2^ = 85% |
| Hurst 2019 | At least one AT and RT group. AT defined as exercise involving large muscle groups in dynamic activities to increase in heart rate and energy expenditure. RT defined as muscle- strengthening activities working against or moving an external resistance (e.g., free weights, machines, elastic bands, body weight). Comparison: either (1) no-exercise control; (2) AT only; or (3) RT only. | 6MWT *vs. no exercise* | 29.6 m (9.1, 50.1) | Small benefit | Small |
|  |  | 6MWT *vs. RT* | 5.8 m (-14.4, 26.6) | No difference | Trivial |
|  |  | TUG *vs. no exercise* | 0.8 s (0.4, 1.2) | Moderate benefit | Small |
|  |  | TUG *vs. RT* | 0.3 s (-0.3, 0.9) | No difference | Trivial |
|  |  | 30-s chair stand *vs. no exercise* | 3.1 repetitions (1.8, 4.4) | Small-moderate benefit | Moderate |
|  |  | 30-s chair stand *vs. RT* | 1.1 repetitions (0.6, 1.6) | Small benefit | Trivial |
| Meereis-Lemos 2020 | Supervised RT combined with another training modality at least twice a week for a minimum of 8 weeks. Comparison: no exercise control group. | TUG | -1.48 s (-2.09, -0.88) | - | I² = 0% |
|  |  | 30s chair stand | 1.79 repetitions (0.76, 2.83) | - | I^2^ = 52% |
|  |  | Chair stand (5 rep) | -2.29 s (-4.19, -0.39) | - | I^2^ = 0% |
| Levin 2017 | A physical intervention or combined physical and cognitive intervention (dual task) with combined motor and cognitive outcomes as an endpoint. Comparison: either 1) passive, 2) health education classes, or 3) lesser training. | Mobility (TUG, stride length, walking speed, chair stand) | - | 7/9 studies using combined exercise training found significant gains. | NR |
| Liu 2017 | Multimodal exercise combines >2 types of exercise strengthening, balance, stretching, and endurance or AT. Comparison: No intervention or attention control without any exercise components. | Gait speed (maximal) | SMD: 0.31 (0.03 to 0.58) | - | NR |
|  |  | 30s chair stand | SMD: -0.26 (-0.50 to -0.02) | - | NR |
|  |  | TUG | SMD: -0.41 (-1.06 to -0.24) | - | NR |
| Van Abbema 2015 | Progressive RT, balance, and AT with or without some additional training components such as cognitive training. Comparison: usual activity or attention control (e.g., health, wellness, or driver education classes) | Preferred gait speed, *RT, balance, AT* | 0.05 m/s (0.00, 0.09) | - | I^2^ = 15.3% |
|  |  | Preferred gait speed, *multimodal exercise* | 0.04 m/s (-0.03, 0.11) | - | I^2^ = 53.8% |
| **General physical activity** | | | | | |
| Frost 2017 | Home- or community-based health promotion interventions (i.e., interventions that enable people to improve or increase control over their health). Comparison: either 1) usual activity, 2) usual activity + two PA and nutrition lectures, 3) monthly general health education sessions, or 4) low intensity flexibility home exercise program. | Self-reported function (various measures) | SMD: 0.19 (-0.57, 0.95) | - | I^2^ = 80% |
|  |  | Multi-domain physical functioning (various measures) | SMD: 0.37 (0.07, 0.68) | - | I^2^ = 31% |
|  |  | Gait speed | SMD: -0.06 (-0.49, 0.37) | - | I^2^ = 50% |
|  |  | TUG | SMD: 0.57 (-0.01, 1.16) | - | I^2^ = 0% |
| Garcia-Hermoso 2020 | Multi-component training (n = 47), RT (n = 24), AT (n = 19), and Tai Chi (n = 4). Most used group-based supervised exercise alone (n = 56) or combined with home-based (n = 21). Most were 1 year; frequency 1 to 7 sessions/week, 10–90 min/session. Comparison: Most control groups were instructed to maintain usual activity levels with or without an additional non-exercise intervention (e.g., health education, social visits, or telephone calls). | Gait speed | SMD: 0.13 (0.03, 0.23) | - | I^2^ = 46.60 |
|  |  | SPPB | SMD: 0.16 (0.01, 0.30) | - | I^2^ = 70.97 |
|  |  | Chair stand | SMD: -0.27 (-0.38, -0.17) | - | I^2^ = 44.42 |
|  |  | TUG | SMD: -0.20 (-0.34, -0.06) | - | I^2^ = 74.64 |
| Grässler 2021 | Physical training intervention (endurance, resistance, coordinative, or multimodal training) with a minimum of 4 weeks and 8 training sessions. Comparison: NR | 6MWT | - | Significant improvements in 2/3 studies, effect size NR | High |
| Howe 2011 | Interventions designed to improve balance, in which participants exercise against an external force, or in response to an unexpected perturbation/stimulus. Multiple exercise types included within the intervention. Comparison: attention-control activities (e.g., attending recreational or educational activities or groups) | TUG | -1.63 s (-2.28, -0.98) s | - | I^2^ = 82% |
|  |  | Gait speed | SMD: 0.04 (-0.10, 0.17) | - | I^2^ = 22% |
| Liberman 2017 | Any exercise; included RT (n = 16), AT (n = 8), AT/RT (n = 6) and other types 9n = 10). Comparison: No intervention/exercise program. | Physical function (various measures) | - | Physical function improved. large effect sizes (>0.74) seen for chair stand test, 400-m walk distance | NR |
| Loureiro 2021 | Multi-component interventions including strength and balance training, flexibility, endurance, gait, and/or functional exercises, treatment of sensory impairments, health education, medical management and/or in home falls risk assessment. Comparison: Usual care, delayed intervention, health education | Mobility/gait | - | 5/6 studies found a significant difference between groups | “Results are heterogeneous” |
| Martin 2013 | Physical therapist led or supervised group exercise. Comparison: individual physical therapy or no exercise control | Functional mobility (TUG, gait speed) | - | 3 studies found improvements in intervention and control groups; no study found between-group differences. | NR |
| Moore 2016 | Community-based PA intervention of six weeks or more (from start to follow-up). Comparison: NR | Overall physical function | - | 6 of 7 studies noted improvements in various measures of physical function. | NR |
| Nicolson 2021 | Therapeutic exercise including AT, RT, functional training, balance training, gait training, flexibility, or 3D (constant movement in a controlled, fluid, repetitive way through all three spatial dimensions, e.g., Tai Chi). Comparison: Usual care, no treatment, other exercise, pharmacotherapy, or health education | Performance based physical function *vs. no exercise* | SMD: 1.55 (-0.50, 3.59) | - | I^2^ = 96% |
|  |  | Performance based physical function *vs. RT* | SMD: -0.17 (-0.64, 0.30) | - | I^2^ = 0% |
| Plummer 2015 | Any physical exercise intervention that compared treatment effects between physical exercise intervention and control groups. Comparison: either 1) active exercise, 2) education control, or 3) inactive no treatment/delayed treatment | Single-task gait speed | 0.06 m/s (0.03, 0.10) | - | I^2^ = 33% |
|  |  | Dual-task gait speed | 0.11 m/s (0.07, 0.15) | - | I^2^ = 47% |
| Yang 2019 | Any types of intervention that were conducted in the community, delivered by any kinds of providers, and that contained multiple interventional components.  Comparison: no exercise | 30s chair stand | 1.19 repetitions (0.37, 2.01) | - | I^2^ = 46% |
|  |  | TUG | -0.55 s (-0.75, -0.35) |  | I^2^ = 60% |
|  |  | 2-minute step test | 6.69 steps (0.20, 13.18) |  | I^2^ = 65% |
|  |  | 6MWT | 33.31 m (19.52, 47.10) |  | I^2^ = 0% |
| **Exercise and nutritional supplements** | | | | | |
| Antoniak 2017 | RT and vitamin D3 supplementation with or without calcium. Comparison: Sedentary, usual care without vitamin D3 supplementation | TUG *vs. exercise only* | 0.21 s (-0.68, 0.26) | - | I^2^ = 0% |
|  |  | TUG *vs. vitamin D only* | -1.57 s (-2.50, -0.64) | - | I^2^ = 0% |
|  |  | SPPB *vs. vitamin D only* | 1.09 points (0.15, 2.03) | - | I^2^ = 0% |
| Devries 2014 | RT and Cr supplementation. Comparison: Placebo-controlled | 30-s chair stand | 1.93 repetitions (0.19, 3.67) | - | I^2^ = 62% |
| Gade 2018 | RT plus protein or essential amino acid supplementation or a modified diet with increased protein content for > 5 weeks. Comparison: RT with/without a non-protein placebo. RT could target all or specific muscle groups. | Physical function (various measures) | - | Of the 13 studies, 2 found significant effect of protein and/or essential amino acids on TUG. No studies found negative effect. | NR |
| Hanach 2019 | Dairy protein supplementation (e.g., whey protein, milk-protein concentrate, casein) or a protein-based dairy product (e.g., ricotta cheese) for ≥12 weeks with or without RT. Comparison: either 1) habitual diet, 2) placebo, or 3) regular dairy product. | SPPB | - | Two studies found improvements; two studies found no effect. | NR |
| Hou 2019 | Protein supplementations containing leucine, whey protein, casein, lean meat, low-fat milk or related mixture and RT 1-4 times/week.  Comparison: RT alone | Gait speed | SMD: -0.04 (-0.50, 0.42) | - | I^2^ = 86.2% |
|  |  | TUG | SMD: -0.66 (-1.41, 0.08) | - | I^2^ = 81.9% |
|  |  | Chair rise time | SMD: -0.06 (-0.54, 0.43) | - | I^2^ = 75.2% |
|  |  | SPPB | - | No significant differences in two trials | NR |
| Stares 2020 | A physical training program and Cr. Comparison: Placebo | - | - | All 4 studies found improvements in functional capacity, primarily through 30s chair stands | NR |
| Ten Haaf 2018 | Multi-nutrient protein or essential amino acid supplementation added to or replacing normal diet with or without RT. Supplements were consumed ≥3 times/week for at least 4 weeks. Comparison: Placebo control or RT | Gait speed *protein only* | SMD: 0.41 (−0.04, 0.85) | - | I^2^ = 76.4% |
|  |  | Gait speed *protein and RT* | SMD: 0.13 (-0.03, 0.28) | - | I^2^ = 0% |
|  |  | Chair rise *protein only* | SMD: 0.10 (−0.08, 0.28) | - | I^2^ = 0% |
|  |  | Chair rise *protein and RT* | SMD: 0.01 (-0.16, 0.17) | - | I^2^ = 0% |
| **Mind-body exercise** | | | | | |
| Bueno de Souza 2018 | Mat Pilates with or without accessories. Comparison: No exercise | 6MWT | SMD: 2.00 (1.44, 2.56) | - | I^2^ = 0% |
|  |  | TUG | SMD: 1.61 (0.45, 2.76) | - | I^2^ = 90% |
| Bullo 2015 | Pilates-identified exercise intervention. Comparison: Not specified except one study that had a non-exercise control group. | Walking and gait measures (various measures) | - | Large effect sizes (1.39) in two studies | NR |
| Ebner 2021 | Yoga, Qi Gong, Tai Chi, Pilates. Comparison: Active and inactive controls | Functional mobility performance *vs. inactive control* | SMD = 0.55 (90% CI 0.20, 0.89) | - | I^2^ = 83% |
|  |  | Functional mobility performance *vs. active control* | SMD = -0.06 (90% CI -0.31, 0.19) | - | I^2^ = 74% |
|  |  | Gait *vs. inactive control* | SMD = 0.47 (90% CI -0.16, 1.10) | - | I^2^ = 94% |
|  |  | Gait *vs. active control* | SMD = 0.05 (90% CI -0.60, 0.70) | - | I^2^ = 85% |
| Fernández-Rodríguez 2020 | At least one exercise intervention described as “Pilates” (Mat, machine, or both. Comparison: Habitual or non-exercise | Function | SMD = 0.51 (0.32, 0.71) | - | I^2^ = 47.7% |
| Howe 2011 | Interventions designed to improve balance, in which participants exercise against an external force, or in response to an unexpected perturbation/stimulus. Tai Chi, qi gong, dance and yoga were included. Comparison: attention-control activities (e.g., attending recreational or educational activities or groups) | Gait speed | SMD: 0.39 (-0.28, 1.06) | - | I^2^ = 73% |
| Liu 2010 | Tai Chi. Comparison: NR | Functional performance (various measures) | - | Improved performance (1 study), TUG (3 studies), and Tinetti gait scale (2 studies). Gait velocity improved in 1 study but not in 3 others. Maximum step length improved in 1 study. No difference in chair rise in 1 study. | NR |
| Qi 2020 | Tai Chi combined with RT. Comparison: Any control or comparison group. | Functional Mobility | - | 3/4 studies found increased mobility in the tai chi and RT vs. control | NR |
|  |  | 6MWT |  | 3 studies found Tai Chi and RT to be superior compared with no exercise or education for 6MWT |  |
| Roland 2011 | Yoga. Comparison: other exercise, non-exercise, or pre/post yoga groups | Gait (walking speed, 6MWT) | - | Improved in 2 studies (effect sizes moderate to high; 6MWT significant in one study, walking speed significance unclear) | NR |
| Sivaramakrishnan 2019 | Yoga. Comparison: Inactive or active controls | Walking speed *vs. inactive control* | SMD: 0.38 (-0.02, 0.78) | - | I^2^ = 72.69 |
|  |  | Walking speed *vs. active control* | SMD: -0.29 (-0.79, 0.22) | - | I^2^ = 57.41 |
| Wang 2021 | Traditional Chinese medicine-based exercises including but not limited to Tai Chi, Ba Duan Jin, and Qigong. Comparison: Placebo, AT, routine care, or educational programs | TUG | MD -2.13 (-2.93, -1.32) | - | I^2^ = 92% |
|  |  | Chair stands (5 reps) | MD -1.24 (-1.99, -0.49) | - | I^2^ = 81% |
|  |  | 30s chair stand | MD 4.40 (0.79, 8.00) | - | I^2^ = 93% |
| **Dance** | | | | | |
| Fernandez-Arguelles 2015 | Dance-based AT, dance and foot tapping or squatting, Turkish folk dance, low impact aerobic dance, Greek traditional dance, ballroom dance, and salsa. Comparison: Other types of exercise or PA | Gait quality (various measures) | - | In all 5 studies, statistically significant improvements were shown regardless of intervention type and period | NR |
| Hwang 2015 | Dance defined as a form of artistic expression through rhythmic movement to music, which does not include aerobic fitness classes taught to music, such as Zumba and step-aerobics. Interventions 1-4x/week for 6 weeks to 8 months, 45 min-2h per session. Comparison: Control groups engaged in other activity or no activity. | Physical function (TUG, sit-stand, gait speed) | - | 23 of 28 measurements in 12 studies (82%) showed significant positive changes | NR |
| Liu 2020 | Dance interventions of at least 6 weeks duration compared to other exercise or no intervention. Comparison: Control groups were required to keep regular daily activities. | Chair stands | -2.90 s (-5.23, -0.58) | - | I^2^ = 89% |
|  |  | TUG | -2.48 s (-4.35, -0.62) | - | I^2^ = 89% |
|  |  | 6MWT (m) | 59.71 m (39.81, 79.62) | - | I^2^ = 30% |
|  |  | Gait speed | 0.07 s (-0.02, 0.17) | - | I^2^ = 67% |
| Rodrigues-Krause 2019 | Regular dance classes of any style for at least 2 weeks. Dance environments included dance studios and stage and/or dance ballrooms. Comparison: Non-exercising control groups and/or groups performing other types of exercise. | Gait ability (gait computer systems, 6MWT, and the TUG test | - | In 17 studies, nearly all induced improvements within and between groups when comparing dancers |  |
| Van Abbema 2015 | Exercise interventions with a dance/rhythmic component. Comparison: Usual activity or attention control (e.g., health, wellness, or driver education classes) | Preferred gait speed | 0.07 m/s (0.03, 0.10) | - | I^2^ = 0% |
| **Other exercise types** | | | | | |
| Bruderer-Hotstetter 2018 | Combined cognitive training and physical exercise interventions. The exercise component was planned, structured and cognitive activity, such as exergames, dancing or TaiChi were also included. Comparison: Attention-controls or no intervention | Physical capacity (composite measure) *static & dynamic exercise* | SMD: 1.9 (-0.60, 4.4) | - | I^2^ = 4% |
|  |  | Physical capacity (composite measure) *exergames* | SMD: 1.8 (-0.66, 4.2) | - | I^2^ = 4% |
| Hortobagyi 2015 | Functional or coordination training, defined as exercise that uses one’s own bodyweight and had subjects perform balance, walking, dance, functional training, and/or AT. Comparison: No exercise | Gait speed | SMD: 0.76 (0.04, 1.4) | - | I^2^ = 90% |
| Howe 2011 | Interventions designed to improve balance, in which participants exercise against an external force, or in response to an unexpected perturbation/stimulus. Gait, coordination, and functional exercises. Comparison: attention-control activities (e.g., attending recreational or educational activities or groups) | TUG | -0.82 s (-1.56, -0.08) | - | I^2^ = 29% |
|  |  | Gait speed | SMD: 0.43 (0.11, 0.75) | - | I^2^ = 0% |
| King 2016 | Exercise programs in water (excluding swimming), with no restriction on depth or temperature of the aquatic environment. Comparison: Land exercise or no-exercise control group | Physical function (walking, 30s chair stand, speed/stamina, TUG, BBS, step test, functional reach) | - | Aquatic exercise group scored better than those in the control group on measures of physical function, but there was little evidence aquatic exercise was better than land-based exercise. | NR |
| Stathokostas 2012 | Flexibility training (excluding tai chi or yoga) as an intervention or control. | Functional outcomes (various measures) | - | 7/22 found positive outcomes, 6 reported no improvement, 10 did not report outcomes | NR |
|  |  | Functional outcomes (various measures) very old | - | In 8 studies of > 80 years, improvements in functional reach, chair stands, 30m walk times, no change in PPT and mixed results for flexibility, strength, balance, TUG | NR |
| Van Abbema 2015 | Stretching exercises. Comparison: usual activity or attention control (e.g., health, wellness, or driver education classes) | Preferred gait speed | 0.06 m/s (-0.01, 0.13) | - | I^2^ = 51.3% |
| Vetrovsky 2019 | Plyometric training (eccentric followed by concentric contraction, e.g., repetitive jumping, hopping, bounding, and skipping) or multicomponent training with plyometric component. Comparison: Either a non-exercising control group or another exercising group | Physical performance (various measures) | - | Mostly positive effects: plyometrics improves physical performance and may be superior to other types of training | NR |
| Waller 2016 | Exercise in an aquatic environment with no limitation on the type of exercise. Comparison: Land exercise or no exercise | Physical function | SMD: 0.70 (0.48, 0.92) | - | I^2^ = 75% |
|  |  | Self-reported function | SMD: 0.88 (0.30, 1.47) | - | I^2^ = 0% |
| 1RM = one-rep max; 6MWT = six-minute walking test; AT = aerobic exercise training; BBS = Berg Balance Scale; Cr = creatine supplementation; m/s = meters per second; NR = not reported; PA = physical activity; PPT = physical performance test; RCT = randomized controlled trial; RT = resistance training; s = seconds; SMD = standardized mean difference; SPPB = Short Physical Performance Battery; TUG = Timed Up and Go test | | | | | |
