# Supplementary material for: Community-based group physical activity and/or nutrition interventions to promote mobility in older adults: an umbrella review
Source: BMC Geriatr. 2022 Jun 29;22:539. doi: 10.1186/s12877-022-03170-9 (PMC9241281; doi:10.1186/s12877-022-03170-9)
Supplement: Supplementary file 6 — Additional file 6. Balance Outcomes. [file 12877_2022_3170_MOESM6_ESM.docx]

**Additional file 6: Balance Outcomes**

| **Study** | | **Intervention/Comparison Description** | **Measure & Unit** | **Meta-Analysis Results**  **(Mean difference, 95% CI)** | **Narrative Results** | **Heterogeneity** |
| --- | --- | --- | --- | --- | --- | --- |
| **Resistance exercise** | | | | | | |
| Howe 2011 | | RT and power training interventions designed to improve balance, in which participants exercise against an external force, or in response to an unexpected perturbation/stimulus. Comparison: attention-control usual activities (e.g., attending recreational or educational activities or groups) | Single leg stance, eyes open | 3.88 s (-0.52, 8.28) | - | I^2^ = 66% |
|  |  |  | Single leg stance, eyes closed | 1.64 s (0.97, 2.31) |  | I^2^ = 0% |
| Liu 2017 | | Progressive RT, in which one exerts an effort against an external resistance that is increased gradually as progress is made. Comparison: No intervention or attention control without any exercise components. | Static standing balance (tandem stance) | 0.74 s (0.00, 1.48) | **-** | NR |
| Tschopp 2011 | | Power training (moderate resistance and an ‘as fast as possible’ movement speed for at least the concentric phase of an exercise). Comparison: Conventional RT (high or moderate resistance and slow concentric movement) | Balance | SMD: 0.91 (-0.17, 1.99) | - | I^2^ = 81% |
| **Aerobic exercise** | | | | | | |
| Bullo 2018 | Supervised or unsupervised Nordic walking. Comparison: Sedentary group, walking, and RT. | | Static balance *vs. no exercise* | SMD: -0.72 (-1.70, 0.26) | - | NR |
|  |  |  | Static balance *vs. walking* | SMD: -0.08 (-0.31, 0.15) |  |  |
|  |  |  | Static balance, composite measure *vs. RT* | SMD: -0.07 (-1.16, 1.03) |  |  |
|  |  |  | Functional balance *vs. no exercise* | SMD: 0.62 (0.18, 1.07) |  | NR |
|  |  |  | Functional balance *vs. walking* | SMD: 0.00 (-0.77, 0.77) |  |  |
| Elboim-Gabyzon 2021 | High-intensity exercise (90–95% peak heart rate, 90% maximal oxygen uptake, at least 75% peak work rate) separated by periods of low to moderate-intensity or rest (e.g., walking/running, cycling). Comparison: No treatment or other exercise | | Static balance | - | Unclear effectiveness in 2/2 studies | High |
| **Combined aerobic and resistance exercise** | | | | | | |
| Bouaziz 2016 | | Multi-modal exercise including AT, RT, balance, stability, flexibility, and/or coordination training. AT defined as exercise involving movement of large muscle groups for a period (e.g., walking, cycling, or rowing). RT defined as progressive training involving an increase in load over time without a specific intensity. Balance training included exercise to increase one’s ability to maintain balance with a threat to stability (e.g., specific balance exercises or Tai Chi). Comparison: Control criteria NR | Balance (various measures) | - | From 14 studies, a significant improvement was found in 7 RCTs and 5 single-group studies; improvements ranged between 5.3% and 88.9%. | NR |
| Martins 2018 | | Modified Otago Exercise program (RT, balance, and walking). Comparison: either 1) original Otago program, 2) non-intervention, or 3) different type of exercise. | Balance (TUG, BBS, 6MWT, OLS, SPPB, chair stand, gait) | - | All 5 RCTs and 2 quasi-experimental studies reported improvements in balance. | 0 |
| Meereis-Lemos 2020I | | Supervised RT combined with another training modality at least twice a week for a minimum of 8 weeks, Comparison: No exercise | BBS | 3.17 points (0.77, 5.57 points) | - | I^2^ = 0% |
| Levin 2017 | | A physical intervention or combined physical and cognitive intervention (dual task) with combined motor and cognitive outcomes as an endpoint. Comparison: either 1) passive, 2) health education classes, or 3) lesser training. | Balance | - | All 3 studies reported significant pre-to-post improvements in balance. | 0 |
| Liu 2017 | | Multimodal exercise combines >2 types of exercise strengthening, balance, stretching, and endurance or AT. Comparison: No intervention or attention control without any exercise components. | Static standing (tandem stance) | 2.60 s (-0.51, 5.70) | **-** | NR |
|  |  |  | Static standing (OLS) | 2.85 s (-0.37, 6.07) | **-** | NR |
|  |  |  | Dynamic standing | SMD: 0.46 (0.22, 0.70) | **-** | NR |
| **General physical activity** | | | | | | |
| Frost 2017 | | Home- or community-based health promotion interventions (i.e., interventions that enable people to improve or increase control over their health). Comparison: either 1) usual activity, 2) usual activity + lectures, 3) monthly general health education sessions, or 4) low intensity flexibility home exercise program. | Balance (various measures) | SMD: 0.33 (0.08, 0.57) | - | I^2^ = 0% |
| Garcia-Hermoso 2020 | | Multi-component training (n = 47), RT (n = 24), AT (n = 19), and Tai Chi (n = 4). Most studies used group-based supervised exercise alone (n = 56) or combined with home-based unsupervised training (n = 21). Most interventions were 1 year; frequency from 1 to 7 sessions/week, 10–90 min/session. Comparison: Most control groups instructed to maintain usual activity with or without non-exercise intervention (e.g., health education, social visits, or calls). | Balance | SMD: 0.31 (0.21, 0.42) | - | I^2^ = 55% |
| Howe 2011 | | Interventions designed to improve balance, in which participants exercise against an external force, or in response to an unexpected perturbation/stimulus. Multiple exercise types included within the intervention. Comparison: attention-control usual activities (e.g., attending recreational or educational activities or groups) | BBS | 1.84 points (0.71, 2.97) | - | I^2^ = 0% |
|  |  |  | Single leg, eyes open | 5.03 s (1.19, 8.87) | - | I^2^ = 82% |
|  |  |  | Single leg, eyes closed | 1.60 s (-0.01, 3.20) | - | I^2^ = 0% |
| Martin 2013 | | Physical therapist led or supervised group exercise. Comparison: Individual physical therapy or no exercise control | Coordinated stability test | - | 2/4 studies found significant improvements vs. control | NR |
| **Mind-body exercise** | | | | | | |
| Bueno de Souza 2018 | | Mat Pilates with or without accessories. Comparison: No exercise training activities. | Static balance (OLS, force platform total sway area) | SMD: 0.48 (0.11, 0.85) | - | I^2^ = 22% |
|  |  |  | Dynamic balance (BBS) | SMD: 0.24 (-0.14, 0.62) | - | I^2^ = 0% |
| Bullo 2015 | | Pilates-identified exercise intervention. Comparison: Not specified except one study that had a non-exercise control group. | Static balance (various measures) | SMD: 0.35 (0.10, 0.60) | - | NR |
|  |  |  | Dynamic balance (various measures) | SMD: 0.77 (0.45, 1.09) | - | NR |
| Howe 2011 | | Interventions designed to improve balance, in which participants exercise against an external force, or in response to an unexpected perturbation/stimulus. Tai Chi, qi gong, dance and yoga were included. Comparison: attention-control usual activities (e.g., attending recreational or educational activities or groups) | BBS | 1.06 points (0.37, 1.76) | - | I^2^ = 34% |
| Ebner 2021 | | Yoga, Qi Gong, Tai Chi, Pilates. Comparison: Active and inactive controls | Static balance *vs. inactive control* | SMD: 0.37 (90% CI 0.10, 0.63) | - | I^2^ = 79% |
|  |  |  | Static balance *vs. active control* | SMD: 0.46 (90% CI 0.16, 0.76) | - | I^2^ = 73% |
| Fernández-Rodríguez 2020 | | At least one exercise intervention described as “Pilates” (Mat, machine, or both. Comparison: Habitual or non-exercise | Balance | SMD: 0.36 (0.21, 0.50) | - | I^2^ = 73.4% |
| Leung 2011 | | Various styles of Tai Chi. Comparison: Either 1) no treatment, 2) education, or 3) physiotherapy exercise | Balance (OLS) | 1.76 s (-7.00, 10.52) | - | NR |
| Liu 2010 | | Tai Chi. Comparison: NR | Static balance (various measures) | - | Results unclear. Some studies found significant improvements in static balance however others did not. | NR |
|  |  |  | Dynamic balance (various measures) | - | All studies showed improvement compared to health education or stretching, but not compared to RT, balance, or walking. | NR |
| Loureiro 2021 | | Multi-component interventions including strength and balance training, flexibility, endurance, gait, and/or functional exercises, treatment of sensory impairments, health education, medical management and/or in home falls risk assessment. Comparison: Usual care, delayed intervention, health education | Balance | - | 5/5 studies found significant difference between groups | “Results are heterogeneous” |
| Qi 2020 | | Tai Chi combined with RT. Comparison: Any control or comparison group. | Dynamic balance (TUG or others) | - | 3 studies found improved dynamic balance compared with no exercise | NR |
| Roland 2011 | | Yoga. Comparison: other exercise, non-exercise, or pre/post yoga groups | Balance (OLS) | - | Improved in 3 studies with effect sizes ranging from <0.10-0.88, significance unclear in 2/3 studies | NR |
| Sivaramakrishnan 2019 | | Yoga. Comparison: Inactive or active controls | Balance *vs inactive control* | SMD: 0.7 (0.19, 1.22) | - | I^2^ = 72% |
|  |  |  | Balance *vs active control* | SMD: 0.32 (-0.02, 0.66) | - | I^2^ = 35% |
| Wang 2021 | | Traditional Chinese medicine-based exercises including but not limited to Tai Chi, Ba Duan Jin, and Qigong. Comparison: Placebo, AT, routine care, or educational programs | Dynamic balance (functional reach) | MD 1.57 (1.22, 1.93) | - | I^2^ = 0% |
|  |  |  | Dynamic balance (BBS) | MD 1.58 (0.31, 2.85) | - | I^2^ = 82% |
|  |  |  | Single leg stance, eyes closed | MD 2.63 (1.94, 3.33) | - | I^2^ = 86% |
|  |  |  | Single leg stance, eyes open | MD 6.13 (4.22, 8.03) | - | I^2^ = 49% |
| **Dance** | | | | | | |
| Fernandez-Arguelles 2015 | Dance-based AT, dance and foot tapping or squatting, Turkish folk dance, low impact aerobic dance, Greek traditional dance, ballroom dance, and salsa dancing. Comparison: Other types of exercise/PA | | Balance (various measures) | - | 5/6 studies showed statistically significant differences between groups. Results given for single studies but not synthesized. | “Measures very heterogeneous” |
| Hwang 2015 | Dance defined as a form of artistic expression through rhythmic movement to music, which does not include aerobic classes taught to music, such as Zumba and step-aerobics. Interventions ranged from 1-4x/week for 6 weeks to 8 months, 45 min-2h per session. Comparison: Control groups engaged in other or no activity | | Balance (sway test or centre of pressure displacement) | - | 8 of 9 measurements in 7 studies showed significant positive changes | NR |
| Liu 2020 | Dance interventions of at least 6 weeks duration compared to other exercise or no intervention. Comparison: Control groups were required to keep regular daily activities. | | Balance (BBS) | - | Improved in the dance intervention groups compared to the control groups in 2 of 4 studies | NR |
|  |  |  | Balance (OLS, center, or pressure) | - | No significant differences in one-leg stance in two studies | NR |
| Rodrigues-Krause 2019 | Regular dance classes of any style for at least 2 weeks. Dance environments included dance studios and stage and/or dance ballrooms. Comparison: Non-exercising control groups and/or groups performing other types of exercise. | | Balance (various measures) | - | All interventions showed within- and between-group improvements, except for 1 which did not detect changes after aerobic dance. | NR |
| **Other** | | | | | | |
| Howe 2011 | | Interventions designed to improve balance, in which participants exercise against an external force, or in response to an unexpected perturbation/stimulus. Gait, coordination, and functional exercises. Comparison: attention-control usual activities (e.g., attending recreational or educational activities or groups) | BBS | 3.48 points (2.01, 4.95) | - | I^2^ = 27% |
|  |  |  | Single leg stance, eyes open | 3.13 s (0.26, 6.01) | - | I^2^ = 28% |
| Lesinski 2015 | | Balance training protocol comprising static/dynamic postural stabilization exercises (combined training was excluded). Comparison: No intervention | Static steady-state balance (various measures) | SMD: 0.51 (0.06, 0.96) | - | I^2^ = 83% |
|  |  |  | Dynamic steady-state balance (e.g., gait speed) | SMD: 0.44 (-0.24, 1.13) | - | I^2^ = 88 % |
|  |  |  | Balance test batteries (e.g., BBS) | SMD: 1.52 (0.65, 2.39) | - | I^2^ = 76 % |
| Vetrovsky 2019 | | Plyometric training (eccentric followed by concentric contraction, e.g., repetitive jumping, hopping, bounding, and skipping) or multicomponent training with plyometric component. Comparison: Either a non-exercising control group or another exercising group | Postural stability (various measures) | - | Plyometric training improved postural stability provided the training program has sufficient volume and intensity. | NR |
| Waller 2016 | | Exercise in an aquatic environment with no limitation on the type of exercise. Comparison: Land exercise or no exercise | Postural stability, *vs. control* | SMD: -0.34 (-0.90, 0.22) | - | I^2^ = 0% |
| Note: 6MWT = six-minute walking test; AT = aerobic exercise training; BBS = Berg Balance Scale; NR = not reported; OLS = one-legged stand test; PA = physical activity; RCT = randomized controlled trial; RT = resistance training; s = seconds; SPPB = Short Physical Performance Battery; SMD = standardized mean difference; TUG = Timed Up and Go test | | | | | | |
