# Supplementary material for: Community-based group physical activity and/or nutrition interventions to promote mobility in older adults: an umbrella review
Source: BMC Geriatr. 2022 Jun 29;22:539. doi: 10.1186/s12877-022-03170-9 (PMC9241281; doi:10.1186/s12877-022-03170-9)
Supplement: Supplementary file 7 — Additional file 7. Falls and Safety Outcomes. [file 12877_2022_3170_MOESM7_ESM.docx]

**Additional file 7: Falls and Safety Outcomes**

| **Study** | **Intervention/Comparison Description** | **Measure & Unit** | **Meta-Analysis Results**  **(Mean difference, 95% CI)** | **Narrative Results** | | **Heterogeneity** |
| --- | --- | --- | --- | --- | --- | --- |
| **Resistance exercise** | | | | | | |
| Raymond 2013 | Lower limb high intensity progressive RT with/without upper limb, or trunk strengthening. Must be land based, within defined %1RM ranges, excluding high velocity power training, or combinations of other exercise. Comparison: Low or moderate-intensity RT | Falls | - | Reported in 1 study in an older population with depression, difference in falls not significant | | NR |
| **Aerobic exercise** | | | | | | |
| Bouaziz 2017 | Supervised (class or small group) AT, defined as any exercise involving movement of large muscle groups for a period (i.e., treadmill walking/running, walking, cycling, rowing, or dancing). No threshold set for frequency, duration, or intensity. Comparison: NR | Adverse events | - | 23/53 studies reported adverse events, 9 reported no events. Adverse events were mostly related to acute medical condition (e.g., stroke, HF, acute respiratory tract infection, unstable angina, and even death). | | NR |
|  |  | Risk of falling | - | One RCT found significant reduction in risk of falls with AT. | | NR |
| Elboim-Gabyzon 2021 | High-intensity exercise (90–95% peak heart rate, 90% maximal oxygen uptake, at least 75% peak work rate) separated by periods of low to moderate-intensity or rest (e.g., walking/running, cycling). Comparison: No treatment or other exercise | Adverse events | - | 7 studies reported no adverse effects related to intervention, 4 studies NR | | High |
| **Combined aerobic and resistance exercise** | | | | | | |
| Bouaziz 2016 | Multi-modal exercise including AT, RT, balance, stability, flexibility, and/or coordination training. AT defined as exercise involving movement of large muscle groups for a period (e.g., walking, cycling, or rowing). RT defined as progressive training involving an increase in load over time without a specific intensity. Balance training included exercise to increase one’s ability to maintain balance with a threat to stability (e.g., specific balance exercises or Tai Chi). Comparison: Control criteria NR | Risk of falling, incidence of falls, global risk of falls | - | 4 of 6 studies reported reduced incidence of falls. 2 studies found a reduction in global risk of falls (22% and 40%). One RCT showed 24.4% reduction in the risk of falling and 6% reduction of the risk of fall related injuries. | | NR |
| Liu 2017 | Multimodal exercise combines >2 types of exercise strengthening, balance, stretching, and endurance or AT. Comparison: No intervention or attention control without any exercise components. | Falls rate | RR = 0.63 (0.49, 0.80) | - | | I^2^ = 0% |
| **General physical activity** | | | | | | |
| Garcia-Hermoso 2020 | Multi-component training (n = 47), RT (n = 24), AT (n = 19), and Tai Chi (n = 4). Most studies used group-based supervised exercise alone (n = 56) or combined with home-based unsupervised training (n = 21). Most interventions were 1 year; frequency from 1 to 7 sessions/week, 10–90 min/session. Comparison: Most control groups were instructed to maintain usual activity levels with or without an additional non-exercise intervention (e.g., health education, social visits, or telephone calls). | Number of falls | RR = 0.89 (0.83, 0.96) | - | I^2^ = 70% | |
|  |  | Fall associated injuries | RR = 0.78 (0.67, 0.92) | - | I^2^ = 47% | |
| Martin 2013 | Physical therapist led or supervised group exercise. Comparison: Individual physical therapy or no exercise control | Falls rate | - | All studies reported a significant decrease in the number of falls compared to the control group, (RR range: 0.60-0.82), effect sizes trivial to small (0.19-0.25). | NR | |
| Nicolson 2021 | Therapeutic exercise including AT, RT, functional training, balance training, gait training, flexibility, or 3D (constant movement in a controlled, fluid, repetitive way through all three spatial dimensions, e.g., Tai Chi). Comparison: Usual care, no treatment, other exercise, pharmacotherapy, or health education | Non-serious adverse events *vs. RT* | RR: 0.79 (0.40, 1.57) | - | I^2^ = 0% | |
|  |  | Risk of falling *vs. no exercise* | - | 2/4 studies reported no difference in risk of falls between groups, 2/4 study reported significant reductions. | High | |
| **Mind-body exercise** | | | | | | |
| Fernández-Rodríguez 2020 | At least one exercise intervention described as “Pilates” (Mat, machine, or both. Comparison: Habitual or non-exercise | Risk of falling | SMD: 0.90 (0.41, 1.38) | - | | I^2^ = 73.3% |
| Leung 2011 | Various styles of Tai Chi. Comparison: Either 1) no treatment, 2) education, or 3) physiotherapy exercise | Falls *vs. no exercise* | OR = 0.85 (0.63 to 1.17) | - | | NR |
|  |  | Falls *vs. other exercise* | OR = 0.43 (0.12 to 1.54) | - | | NR |
| Liu 2010 | Tai Chi. Comparison: NR | Rate of falling or fear of falling (various measures) | - | Improvements in subjective fear of falling, number of falls, time to first fall in some studies. | | NR |
| Loureiro 2021 | Multi-component interventions including strength and balance training, flexibility, endurance, gait, and/or functional exercises, treatment of sensory impairments, health education, medical management and/or in home falls risk assessment. Comparison: Usual care, delayed intervention, health education | Rate of falls | - | 3/6 studies found significant difference between groups | | “Results are heterogeneous” |
| **Dance** | | | | | | |
| Rodrigues-Krause 2019 | Regular dance classes of any style for at least 2 weeks. Dance environments included dance studios and stage and/or dance ballrooms. Comparison: Non-exercising control groups and/or groups performing other types of exercise. | Fall prevention | - | Dancing was suggested as a useful intervention for fall prevention in 13 studies. | | NR |
| **Other** | | | | | | |
| Vetrovsky 2019 | Plyometric training (eccentric loading followed by a concentric contraction, e.g., repetitive jumping, hopping, bounding, and skipping) or multicomponent training with plyometric component. Comparison: Either a non-exercising control group or another exercising group | Fractures and injurious falls during 5-year follow up period | - | Authors note an overall positive effect | | NR |
|  |  | Safety | - | 5 studies reported no injuries or adverse events, 4 studies did not report on adverse events at all, 1 study reported 4 falls during supervised sessions, 14 physician consultations for injuries, 10 reports of overuse symptoms, one hospital visit; 1 study reported a maximum of 1.4% incurred an injury that resulted in study dropout | | NR |
| Note: 1RM = one-rep max; AT = aerobic exercise training; NR = not reported; OR = odds ratio; RCT = randomized controlled trial; RR = relative risk; RT = resistance training | | | | | | |
