# Supplementary material for: Community-based group physical activity and/or nutrition interventions to promote mobility in older adults: an umbrella review
Source: BMC Geriatr. 2022 Jun 29;22:539. doi: 10.1186/s12877-022-03170-9 (PMC9241281; doi:10.1186/s12877-022-03170-9)
Supplement: Supplementary file 8 — Additional file 8. Muscle Strength Outcomes. [file 12877_2022_3170_MOESM8_ESM.docx]

**Additional file 8: Muscle Strength Outcomes**

| **Study** | **Intervention/Comparison Description** | **Measure & Unit** | **Meta-Analysis Results**  **(Mean difference, 95% CI)** | **Narrative Results** | **Heterogeneity** |
| --- | --- | --- | --- | --- | --- |
| **Resistance exercise** | | | | | |
| Borde 2015 | Machine-based RT of low, moderate or high intensity. Comparison: inactive control | Muscle strength (upper and lower) | SMD: 1.57 (1.20, 1.94) | - | I^2^ = 80% |
|  |  | Muscle morphology | SMD: 0.42 (0.18, 0.66) | - | I^2^ = 0% |
| Katsoulis 2019 | Low (<50% 1RM), moderate or high (>70% 1RM) intensity power training. Post-intervention values compared to pre-intervention values. | Muscle power *low intensity* | - | Average Pre-Post Change: +21.5% | NR |
|  |  | Muscle power *moderate intensity* | - | Average Pre-Post Change: +33.4% |  |
|  |  | Muscle power *high intensity* | - | Average Pre-Post Change: +26.8% |  |
|  |  | Muscle strength *low intensity* | - | Average Pre-Post Change: +24.3% |  |
|  |  | Muscle strength *moderate intensity* | - | Average Pre-Post Change: +27.2% |  |
|  |  | Muscle strength *high intensity* | - | Average Pre-Post Change: +22.4% |  |
| Liu 2017 | Progressive RT, in which one exerts an effort against an external resistance that is increased gradually as progress is made. Comparison: No intervention or attention control without any exercise components. | Lower extremity muscle strength | SMD: 0.33 (0.10, 0.56) | - | I^2^ = 46% |
| Raymond 2013 | Lower limb high intensity progressive RT with/without upper limb, or trunk strengthening. Must be land based, within defined %1RM ranges, excluding high velocity power training, or combinations of other exercise. Comparison: Low or moderate-intensity RT | Lower-limb strength, *vs low intensity* | SMD: 0.83 (-0.02, 1.68) | - | I^2^ = 66% |
|  |  | Lower-limb strength, *vs moderate intensity* | SMD: 0.79 (0.40, 1.17) | - | I^2^ = 50% |
|  |  | Lower-limb strength, *vs maximal intensity* | - | 7.3-11.2% increase for men and 2.3-15.2% increase for women across all intensities | NR |
|  |  | Power and torque | - | High, maximal and moderate RT improved peak torque, no difference between high and moderate | NR |
|  |  | Lower body endurance | - | No difference found in healthy older populations | NR |
| Straight 2016 | RT, defined as muscle-strengthening activities that use major muscle groups and could include free weights, machines, and resistance bands. Comparison: Usual activity or any sham exercise group (e.g., balance and flexibility) | Lower extremity muscle power (watts) | SMD: 0.34 (0.21, 0.47) | - | I^2^ = 45.9 |
| Tschopp 2011 | Power training (moderate resistance and an ‘as fast as possible’ movement speed for at least the concentric phase of an exercise). Comparison: Conventional RT (high or moderate resistance and slow concentric movement) | Strength | SMD: 0.14 (−0.10, 0.38) | - | I^2^ = 0% |
|  |  | Power | SMD: 0.42 (−0.02, 0.85) | - | I^2^ = 59.2% |
|  |  | Muscle volume and muscle mass | SMD: 0.22 (−0.37, 0.82) | - | I^2^ = 0% |
| **Aerobic exercise** | | | | | |
| Bouaziz 2017 | Supervised (class or small group) AT, defined as any exercise involving movement of large muscle groups for a period of time (i.e., treadmill walking/running, walking, cycling, rowing, or dancing). No threshold set for frequency, duration, or intensity. Comparison: NR | Muscle strength (various measures) | - | Improvements in strength found in 8 RCTs and 3 non RCTs; one non-RCT of cycle ergometry training found no effect | Heterogeneity in outcome measurements |
| Bullo 2018 | Supervised or unsupervised Nordic walking. Comparison: Sedentary group, walking training, and RT. | Upper limb strength (bicep curls,) *vs. no exercise* | SMD: 0.66 (0.43, 0.90) | - | NR |
|  |  | Upper limb strength (bicep curls, handgrip) v*s. walking* | SMD: 0.16 (-0.21, 0.52) | - | NR |
|  |  | Upper limb strength (bicep curls, handgrip) *vs. RT* | SMD: -0.18 (-1.64, 1.27) | - | NR |
|  |  | Lower limb strength *vs. no exercise* | SMD: 0.43 (0.20, 0.66) | - | NR |
|  |  | Lower limb strength vs. *walking* | SMD: -0.10 (-0.75, 0.55) | - | NR |
|  |  | Lower limb strength *vs. RT* | SMD: 0.39 (-1.03, 1.8) | - | NR |
| Elboim-Gabyzon 2021 | High-intensity exercise (90–95% peak heart rate, 90% maximal oxygen uptake, at least 75% peak work rate) separated by periods of low to moderate-intensity or rest (e.g., walking/running, cycling).  Comparison: No treatment or other exercise | Upper limb strength | - | Improved in 2/3 studies | High |
|  |  | Lower limb strength | - | Improved in 3/7 studies compared to non-exercise controls but no beneficial effect observed over moderate-intensity training, effect size small. Contrasting or no effect in the remaining 4 studies. |  |
| **Aerobic and resistance** | | | | | |
| Bouaziz 2016 | Multi-modal exercise including AT, RT, balance, stability, flexibility, and/or coordination training. AT defined as exercise involving movement of large muscle groups for a period of time (e.g., walking, cycling, or rowing). RT defined as progressive training involving an increase in load over time without a specific intensity. Balance training included exercise to increase one’s ability to maintain balance with a threat to stability (e.g., specific balance exercises or Tai Chi). Comparison: criteria NR | Upper and lower extremity muscle strength (various measures) | - | Significant improvement was found in 5 RCTs and 1 non-RCT; and in 5 RCTs and 6 non-RCTs when post training values were compared with baseline ones (muscle strength gain ranging from 1.4% to 95.0%) | NR |
| Levin 2017 | A physical intervention or combined physical and cognitive intervention (dual-task) with combined motor and cognitive outcomes as an endpoint. Comparison: either 1) passive, 2) health education classes, or 3) lesser training. | Muscle strength outcomes | - | Only 1 of 3 studies noted a significant improvement in muscular strength. | NR |
| Liu 2017 | Multimodal exercise combines >2 types of exercise RT, balance, stretching, and/orAT. Comparison: No intervention or attention control without any exercise components. | Lower extremity muscle strength | SMD: 0.16 (0.02, 0.31) | - | I^2^ = 8% |
| **General physical activity** | | | | | |
| Frost 2017 | Home- or community-based health promotion interventions (i.e., interventions that enable people to improve or increase control over their health). Comparison: either 1) usual activity, 2) usual activity + two PA and nutrition lectures, 3) monthly general health education sessions, or 4) low intensity flexibility home exercise programme. | Muscle strength (various measures) | SMD: 0.44 (0.11, 0.77) | - | I^2^ = 47% |
| Garcia-Hermoso 2020 | Multi-component training (n = 47), RT (n = 24), AT (n = 19), and Tai Chi (n = 4). Most studies used group-based supervised exercise alone (n = 56) or combined with home-based unsupervised training (n = 21). Most interventions were 1 year; frequency from 1 to 7 sessions/week, 10–90 min/session. Comparison: Most control groups were instructed to maintain usual activity levels with or without an additional non-exercise intervention (e.g., health education, social visits, or telephone). | Knee extension strength | SMD: 0.28 (0.13, 0.44) | - | I^2^ = 72.89% |
| Labott 2019 | Any RCT that could include aquatic exercise, walking, flexibility, TRX-training, home-trainer exercise, RT in different forms, vibration platform, dance, Tai Chi, exergames, balance training, calisthenics, and multi-dimensional training. Comparison: Control criteria NR | Handgrip strength | SMD: 0.28 (0.13, 0.44) | - | I^2^ = 56% |
| Liberman 2017 | Any exercise; included RT (n = 16), AT (n = 8), AT/RT (n = 6) and other types 9n = 10). Comparison: No intervention/exercise program | Lower body muscle strength | - | One study found increase in strength vs. a control, another found no difference | NR |
|  |  | Muscle mass | - | Improvements found after training. | NR |
| Loureiro 2021 | Multi-component interventions including strength and balance training, flexibility, endurance, gait, and/or functional exercises, treatment of sensory impairments, health education, medical management and/or in home falls risk assessment. Comparison: Usual care, delayed intervention, health education | Strength | - | 3/4 studies found a significant difference between groups | “Results are heterogeneous” |
| Yang 2019 | Any types of intervention that were conducted in the community, delivered by any kinds of providers, that contained multiple components. Comparison: No exercise | Arm curls | 2.70 repetitions (2.00, 3.41) | - | I^2^ = 74% |
| **Exercise + nutrition** | | | | | |
| Antoniak 2017 | RT and vitamin D3 supplementation with or without calcium. Comparison: Sedentary, usual care without vitamin D3 supplementation | Lower Limb Muscle Strength *vs. exercise only* | SMD: 0.98 (0.73, 1.24) | - | I^2^ = 70% |
|  |  | Lower Limb Muscle Strength *vs. vitamin D3 only* | 2.69 (0.95, 4.42) (units NR) | - | I^2^ = 63% |
|  |  | Hand Grip Strength *vs. vitamin D3 only* | 0.85 Nm (-1.93, 3.63) | - | I^2^ = 0% |
| Devries 2014 | RT and Cr supplementation. Comparison: Placebo-controlled | Leg press 1RM | 3.25 kg (0.47, 6.03) | - | I^2^ = 42% |
|  |  | Chest press 1RM | 1.74 kg (0.56, 2.91) | - | I^2^ = 54% |
|  |  | Knee extension 1RM | -0.44 kg, (-2.62, 1.75) | - | I^2^ = 57% |
|  |  | Bicep curl 1RM | 0.47 kg (-1.48, 2.43) | - | I^2^ = 74% |
|  |  | Isokinetic strength | -1.03 Nm (-4.97, 2.91) | - | NR |
|  |  | Isometric strength | SMD: 0.43 (-0.31, 1.16) | - | I^2^ = 84% |
| Finger 2015 | RT and protein for ≥6 weeks. Protein supplements ranged from 0.3 to 0.8 g/kg/day (mean = 0.46 g/kg/day) or 6 to 40 g/day (mean = 20.7 g/day) or high protein diet. Comparison: RT alone (without supplementation) or exercise combined with non-protein placebo supplementation. | Muscle mass | SMD: 0.14 (-0.05, 0.32) | - | I^2^ = 0% |
|  |  | Muscle strength | SMD: 0.13 (-0.06, 0.32) | - | I^2^ = 0% |
| Gade 2018 | RT and protein or essential amino acid supplementation or a modified diet with increased protein content for > 5 weeks. Comparison: RT with/without a non-protein placebo. RT could target all or specific muscle groups. | Upper and lower body strength (various measures) | - | 3/15 studies found a positive effect of protein and/or essential amino acid; one found a trend toward increase strength; one study found a decrease in handgrip strength | NR |
| Hanach 2019 | Dairy protein supplementation (e.g., whey protein, milk-protein concentrate, casein) or a protein-based dairy product (e.g., ricotta cheese) for ≥12 weeks with or without RT. Comparison: either 1) habitual diet, 2) placebo, or 3) regular dairy product. | Hand grip strength | 0.84 kg (−0.24, 1.93) | - | I^2^ = 82% |
|  |  | Leg press strength | 0.37 kg (−4.79, 5.53) | - | I^2^ = 18% |
|  |  | Appendicular muscle mass | 0.13 kg (0.01, 0.26) | - | I^2^ = 14% |
| Hou 2019 | Protein supplementations containing leucine, whey protein, casein, lean meat, low-fat milk or related mixture and RT 1-4 times/week.  Comparison: RT alone | Handgrip strength | 0.29 kg (0.08, 0.50) | - | I^2^ = 0% |
|  |  | Knee extension strength | 0.27 kg (0.06, 0.47) | - | I^2^ = 37.6% |
|  |  | Leg press strength | 0.33 kg (0.01, 0.64) |  | I^2^ = 19% |
| Stares 2020 | A physical training program and Cr. Comparison: Placebo | Strength (1-RM bench press or 1-RM leg press) | - | In females, RT + Cr, vs. RT alone resulted in strength improvements in all three studies. In males, 2 of 7 studies noted improvements in RT + Cr vs. RT alone. In studies including both men and women, 1 of 4 studies found an improvement from RT + Cr vs. Cr alone; 1 only noted an improvement with a specific dosing schedule. | NR |
| Ten Haaf 2018 | Multi-nutrient protein or essential amino acid supplementation added to or replacing normal diet with or without RT. Supplements were consumed ≥3 times/week for at least 4 weeks compared with a (different) control group. | Handgrip *protein vs. control* | SMD: 0.58 (-0.08, 1.24) | - | I^2^ = 89.6% |
|  |  | Lower extremity strength *protein vs. control* | SMD: 0.03 (−0.20, 0.27) | - | I^2^ = 0% |
|  |  | Thigh muscle cross-sectional area *Protein and RT vs. RT alone* | SMD: 0.09 (-0.23, 0.42) | - | I^2^ = 0% |
|  |  | Upper body strength *Protein and RT vs. RT alone* | SMD: 0.11 (-0.07, 0.29) | - | I^2^ = 0% |
|  |  | Lower body strength *Protein and RT vs. RT alone* | SMD: 0.10 (-0.06, 0.27) | - | I^2^ = 23.4% |
| **Mind-body exercise** | | | | | |
| Bueno de Souza 2018 | Mat Pilates with or without accessories. Comparison: No exercise training activities. | Lower limb muscle strength (pooled 5 rep and 30 sec chair stand, manual muscle test) | SMD: 1.13 (0.30, 1.96) | - | I^2^ = 87% |
| Bullo 2015 | Pilates-identified exercise training intervention. Comparison: Not specified except one study that had a non-exercise control group. | Muscle strength | ES = 1.23 (0.844, 1.625) | Large effect size | NR |
| Ebner 2021 | Yoga, Qi Gong, Tai Chi, Pilates. Comparison: Active and inactive controls | Upper body strength *vs. inactive control* | SMD: 0.79 (90% CI 0.39, 1.19) | - | I^2^ = 82% |
|  |  | Upper body strength *vs. active control* | SMD: -0.17 (90% CI -0.51, 0.16) | - | I^2^ = 48% |
|  |  | Lower body strength *vs. inactive control* | SMD: 0.91 (90% CI 0.18, 1.64) | - | I^2^ = 86% |
|  |  | Lower body strength *vs. active control* | SMD: -0.27 (90% CI -0.57, 0.03) | - | I^2^ = 61% |
|  |  | Overall strength (upper and lower body pooled) *vs. inactive control* | SMD: 0.87 (90% CI 0.43, 1.30) | - | I^2^ = 94% |
|  |  | Overall strength (upper and lower body pooled) *vs. active control* | SMD: -0.22 (90% CI -0.43, -0.01) | - | I^2^ = 52% |
| Fernández-Rodríguez 2020 | At least one exercise intervention described as “Pilates” (Mat, machine, or both. Comparison: Habitual or non-exercise | Strength | SMD: 0.63 (0.44, 0.81) | - | I^2^ = 67.3% |
| Liu 2010 | Tai Chi. Comparison: NR | Grip strength | - | No difference between groups | NR |
|  |  | Lower extremity strength |  | Improvements in knee and ankle flexors and extensors; mixed findings for trunk flexibility |  |
| Qi 2020 | Tai Chi combined with RT. Comparison: Any control or comparison group. | Lower limb strength | - | Two trials found improvements in muscle strength; one found improvements only in those with the weakest lower limb strength; one study improved left hip flexion and extension but not right | NR |
| Roland 2011 | Yoga. Comparison: other exercise, non-exercise, or pre/post yoga groups | Muscle strength | - | Three studies found improvements in measures of muscle strength; effect sizes were small and not clinically meaningful. | NR |
| Sivaramakrishnan 2019 | Yoga. Comparison: Inactive or active controls | Lower limb strength *vs. inactive control* | SMD: 0.45 (0.22, 0.68) | - | I^2^ = 32.70% |
|  |  | Lower limb strength *vs. active control* | SMD: 0.49 (0.1, 0.88) | - | I^2^ = 47.44% |
| Wang 2021 | Traditional Chinese medicine-based exercises including but not limited to Tai Chi, Ba Duan Jin,and Qigong. Comparison: Placebo, AT, routine care, or educational programs | Handgrip strength | SMD: 0.56 (0.36, 0.76) | - | I^2^ = 38% |
|  |  | Knee extension strength | SMD: 0.56 (0.26, 0.86) | - | I^2^ = 52% |
| **Dance** | | | | | |
| Fernandez-Arguelles 2015 | Dance-based AT, dance and foot tapping or squatting, Turkish folk dance, low impact aerobic dance, Greek traditional dance, ballroom dance, and salsa dancing compared to other types of exercise or PA | Muscle strength | - | 2/5 studies found improvements, mixed findings across studies | NR |
| Rodrigues-Krause 2019 | Regular dance classes of any style for at least 2 weeks. Dance environments included dance studios and stage and/or dance ballrooms. Comparison: Non-exercising control groups and/or groups performing other types of exercise. | Muscle strength (seat-and stand test, performed either 5 or 10 times or for 30 s) | - | All but 2 of 15 studies found a significant between-group difference. | NR |
| **Other** | | | | | |
| Moran 2018 | Jump training program, defined as lower body unilateral and bilateral bounds, jumps and hops compared to a not-specified control group | Muscular power | SMD: 0.66 (0.33, 0.98) | - | I^2^ = 51% |
| Vetrovsky 2019 | Plyometric training (eccentric loading followed by a concentric contraction, e.g., repetitive jumping, hopping, bounding, and skipping) or multicomponent training with plyometric component. Comparison: Either a non-exercising control group or another exercising group | Handgrip strength | - | Most studies found an improvement in muscle strength when comparing a plyometric or combined training with a control group, comparisons between different training modalities yielded mixed results. | NR |
| Waller 2016 | Exercise in an aquatic environment with no limitation on the type of exercise. Comparison: Land exercise or no exercise | Maximum strength *vs control* | SMD: 0.46 (0.20, 0.72) | - | I^2^ = 13% |
|  |  | Muscular power *vs control* | SMD: 0.35 (-0.08, 0.79) | - | I^2^ = 39% |
|  |  | Muscular endurance *vs control* | SMD: 2.40 (0.63, 4.18) | - | I^2^ = 95% |
|  |  | Respiratory muscle function, *vs control* | SMD: 0.42 (-0.05, 0.90) | - | I^2^ = 0% |
|  |  | Maximum strength *vs land exercise* | SMD: 0.09 (-0.31, 0.48) | - | I^2^ = 39% |
|  |  | Muscular power *vs land exercise* | SMD: 1.31 (0.60, 2.01) | - | NR |
|  |  | Muscular endurance *vs land exercise* | SMD: -0.31 (-0.99, 0.37) |  | I^2^ = 43% |
|  |  | Respiratory muscle function *vs land exercise* | SMD: 0.38 (-0.25, 1.00) | - | NR |
| Wirth 2020 | I: Oral protein intake, 2wk minimal duration, including energy-restriction or not, and including exercise or not  C: Low-protein diet, no protein supplementation, or non-protein placebo | Handgrip strength | MD: 0.26 kg (-0.51, 1.04) | - | I^2^ = 56% |
|  |  | Leg press strength | MD: 1.97 kg (-2.78, 6.72) | - | I^2^ = 0% |
| Note: 1RM = one-rep max; AT = aerobic exercise training; Cr = creatine supplementation; ES = effect size; kg = kilogram; Nm = Newton-meter; NR = not reported; PA = physical activity; RCT = randomized controlled trial; RT = resistance training; s = seconds; SMD = standardized mean difference | | | | | |
