# Supplementary material for: Community-based group physical activity and/or nutrition interventions to promote mobility in older adults: an umbrella review
Source: BMC Geriatr. 2022 Jun 29;22:539. doi: 10.1186/s12877-022-03170-9 (PMC9241281; doi:10.1186/s12877-022-03170-9)
Supplement: Supplementary file 9 — Additional file 9. Health-Related Quality of Life and Wellbeing Outcomes. [file 12877_2022_3170_MOESM9_ESM.docx]

**Additional file 9: Health-Related Quality of Life and Self-reported Wellbeing Outcomes**

| **Study** | **Intervention/Comparison Description** | **Measure & Unit** | **Meta-Analysis Results**  **(Mean difference, 95% CI)** | **Narrative Results** | **Heterogeneity** |
| --- | --- | --- | --- | --- | --- |
| **Resistance exercise** | | | | | |
| Liu 2017 | Progressive RT, in which one exerts an effort against an external resistance that is increased gradually as progress is made. Comparison: No intervention or attention control without any exercise components. | ADL | SMD: 0.13 (-0.04, 0.29) | - | I^2^ = 1% |
| Raymond 2013 | Lower limb high intensity progressive RT with/without upper limb, or trunk strengthening. Must be land based, within defined %1RM ranges, excluding high velocity power training, or combinations of other exercise. Comparison: Low or moderate-intensity RT | Disability (Health Assessment Questionnaire Disability Index) | - | One study found reduced disability with both high and low intensities with no difference between groups. | NR |
|  |  | Quality of life (SF-36) | - | No improvement with moderate or high intensity in one study, another showed moderate improvements in vitality for high vs. low intensity | NR |
| **Aerobic exercise** | | | | | |
| Bouaziz 2017 | Supervised (class or small group) AT, defined as any exercise involving movement of large muscle groups for a period of time (i.e., treadmill walking/running, walking, cycling, rowing, or dancing). No threshold set for frequency, duration, or intensity. Comparison: NR | Quality of life (SF-36, MacNew global score) | - | 3 RCTs found improvements in quality of life ranging from 17.1 to 178% | NR |
| Bullo 2018 | Supervised or unsupervised Nordic walking. Comparison: Sedentary group, walking training, and RT. | Quality of life *vs walking* | SMD: 0.53 (-0.11, 1.17) | - | NR |
|  |  | Quality of life *vs RT* | SMD: 0.93 (0.23, 1.64) |  |  |
| Elboim-Gabyzon 2021 | High-intensity exercise (90–95% peak heart rate, 90% maximal oxygen uptake, at least 75% peak work rate) separated by periods of low to moderate-intensity or rest (e.g., walking/running, cycling). Comparison: No treatment or other exercise | Quality of life (SF-36) | - | Improved in 2/2 studies | High |
| **Combined aerobic and resistance exercise** | | | | | |
| Bouaziz 2016 | Multi-modal exercise including AT, RT, balance, stability, flexibility, and/or coordination training. AT defined as exercise involving movement of large muscle groups for a period (e.g., walking, cycling, or rowing). RT defined as progressive training involving an increase in load over time without a specific intensity. Balance training included exercise to increase one’s ability to maintain balance with a threat to stability (e.g., specific balance exercises or Tai Chi). Comparison: Control criteria NR | Quality of life (SF-36) | - | Improved in three studies ranging from 18.0% to 35.9%. | NR |
| Garcia-Hermoso 2020 | Multi-component training (n = 47), RT (n = 24), AT (n = 19), and Tai Chi (n = 4). Most studies used group-based supervised exercise alone (n = 56) or combined with home-based unsupervised training (n = 21). Most interventions were 1 year; frequency from 1 to 7 sessions/week, 10–90 min/session. Comparison: Most control groups were instructed to maintain usual activity levels with or without an additional non-exercise intervention (e.g., health education, social visits, or telephone calls). | Health-related quality of life, physical functioning (SF-36 or SF-12) | SMD: 0.03 (-0.03, 0.10) | - | I^2^ = 46% |
|  |  | Health-related quality of life, mental health (SF-36 or SF-12) | SMD: 0.05 (−0.01, 0.11) |  | I^2^ = 28% |
| Liu 2017 | Progressive RT, in which one exerts an effort against an external resistance that is increased gradually as progress is made. Comparison: No intervention or attention control without any exercise components. | ADL | SMD: 0.37 (-0.07, 0.80) | - | I^2^ = 57% |
| **General physical activity** | | | | | |
| Frost 2017 | Home- or community-based health promotion (i.e., interventions that enable people to improve or increase control over their health). Comparator groups were a) usual activity, b) usual activity + two PA and nutrition lectures, c) monthly general health education sessions, or d) low intensity flexibility home exercise program. | Quality of life | - | 2 studies found no significant differences in overall quality of life in exercise and exercise and nutrition interventions | NR |
| Martin 2013 | Physical therapist led or supervised group exercise. Comparison: Individual physical therapy or no exercise control | Health-related quality of life (SF-36) | - | Measured in two studies, no statistical significance for one study, significant improvements in the other with trivial effect size | NR |
| **Mind-body exercise** | | | | | |
| Bullo 2015 | Pilates-identified exercise training intervention. Comparison: Not specified except one study that had a non-exercise control group. | ADL, mood states and quality of life (variety of tools) | SMD: 0.943 (0.631, 1.255) | Large effect size | NR |
| Sivaramakrishnan 2019 | Yoga. Comparison: Inactive or active controls | Perceived mental health *vs inactive control* | SMD: 0.6 (0.33, 0.87) | - | I^2^ = 55% |
|  |  | Perceived mental health *vs active control* | SMD: 0.26 (−0.03, 0.55) |  | I^2^ = 0% |
|  |  | Perceived physical health *vs inactive control* | SMD: 0.61 (0.29, 0.94) |  | I^2^ = 59% |
|  |  | Social health *vs inactive control* | SMD: 0.27 (−0.15, 0.69) |  | I^2^ = 52% |
|  |  | Vitality *vs inactive control* | SMD: 0.31 (0.03, 0.59) |  | I^2^ = 0% |
| **Dance** | | | | | |
| Liu 2020 | Dance interventions of at least 6 weeks duration compared to other exercise or no intervention. Comparison: Control groups were required to keep regular daily activities. | General health (SF-36) | - | Measured in two studies; significantly improved after exercise | NR |
| Rodrigues-Krause 2019 | Regular dance classes of any style for at least 2 weeks. Dance environments included dance studios and stage and/or dance ballrooms. Comparison: Non-exercising control groups and/or groups performing other types of exercise. | Quality of life | - | Assessed in 7 studies with all the studies showing dancing-related improvements | NR |
| **Other exercise types** | | | | | |
| Vetrovsky 2019 | Plyometric training (eccentric loading followed by a concentric contraction, e.g., repetitive jumping, hopping, bounding, and skipping) or multicomponent training with plyometric component. Comparison: Either a non-exercising control group or another exercising group | Quality of life and/or daily function (questionnaires) | - | Results were more or less positive, but did not show any superiority of plyometrics over other types of training. | NR |
| Note: 1RM = one-rep max; ADL = activities of daily living; AT = aerobic exercise training; NR = not reported; RCT = randomized controlled trial; RT = resistance training; SF-12 = 12-Item Short Form Survey; SF-36 = 36-Item Short Form Survey; SMD = standardized mean difference | | | | | |
